# Supplementary material for: Beyond volume: Unraveling the genetics of human brain geometry
Source: Sci Adv. 2025 Jun 13;11(24):eadr1644. doi: 10.1126/sciadv.adr1644 (PMC12165004; doi:10.1126/sciadv.adr1644)
Supplement: Supplementary file 1 — Supplementary Text Figs. S1 to S39 Legends for tables S1 to S22 References [file sciadv.adr1644_sm.pdf]

Science Advances
AAAS

Supplementary Materials for

Beyond volume: Unraveling the genetics of human brain geometry

Sabrina A. Primus *et al.*

Corresponding author: Konrad Oexle, konrad.oexle@helmholtz-munich.de; Kaustubh R. Patil, k.patil@fz-juelich.de

Sci. Adv. **11**, eadr1644 (2025)
DOI: 10.1126/sciadv.adr1644

The PDF file includes:

Supplementary Text
Figs. S1 to S39
Legends for tables S1 to S22
References

Other Supplementary Material for this manuscript includes the following:

Tables S1 to S22

Supplementary Text

Examination of SNPs Associated with Amygdala Volume

Neither for the left nor for the right amygdala did the GWAS on the Laplace-Beltrami spectrum (LBS) reveal genome-wide significant signals. We hypothesized that this is due to the simple shape of the amygdala, which is already represented by volume and surface area, both being controlled for in our study. Therefore, we reran MOSTest on non-normalized eigenvalues and did not include surface area as a covariate. We compared the results to the 12 loci that were reported by a recent study on whole amygdala volume (*98*) (Table S20). We replicated the same 4 loci in left and right amygdala and an additional one only in the right hemisphere at 0.05 significance level after FDR correction while our original GWAS on normalized and corrected LBS replicated only 2 loci each at the same threshold, thus supporting our hypothesis.

Outlier Removal

We performed a GWAS re-analysis after removing LBS-outliers using the same procedure as described in the main text (Methods – Multivariate Genome-wide Association Analysis). We defined an individual as an outlier if at least one eigenvalue was more extreme than mean$\pm$4SD across individuals. If we found more than 0.1% of the sample size as outliers (20 out of 19871 individuals including samples without data of total brain volume) in at least one eigenvalue, we removed these individuals and ran the GWAS for this brain structure (Table S7, Fig. S11). CCA was also repeated in the same way (Methods – Canonical Correlation Analysis with PRS) after outlier removal (Fig. S12).

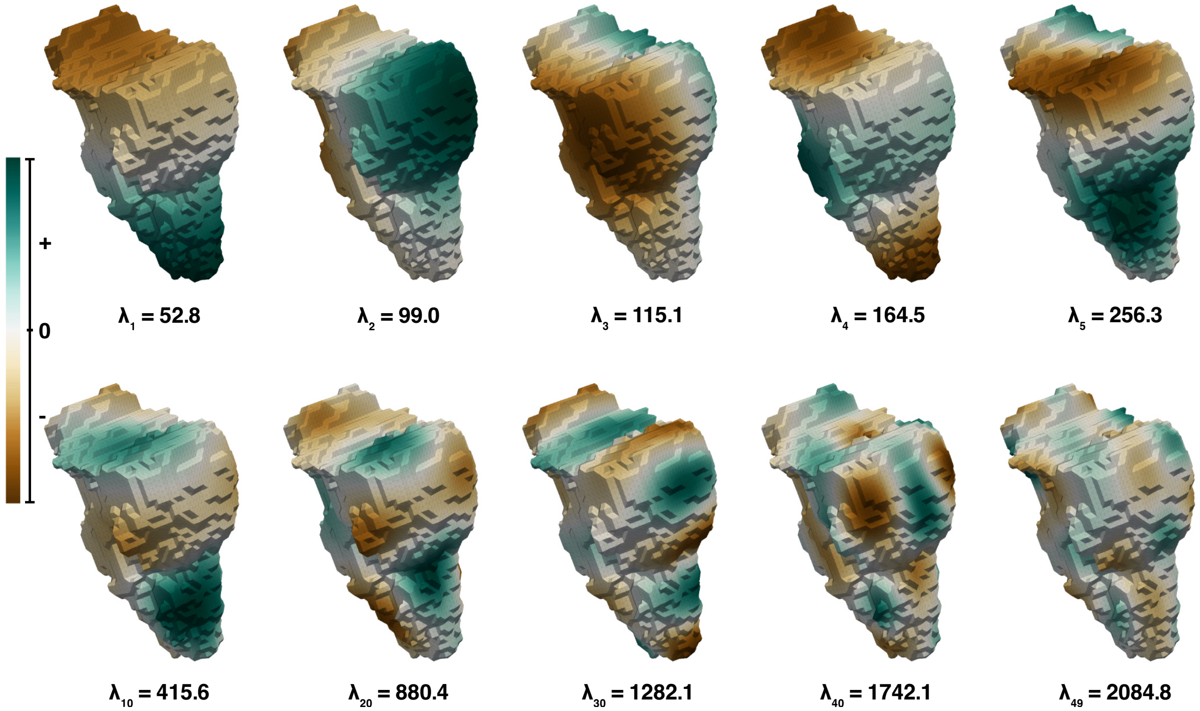

Fig. S1. Eigenfunctions of Brain Stem. Brain stem of an individual in UK Biobank colored by eigenfunctions f of corresponding eigenvalues λ which are stated underneath and were scaled to unit volume. Blue colors represent positive values of eigenfunctions while brown colors represent negative ones. Images of brain meshes were reproduced by kind permission of UK Biobank.

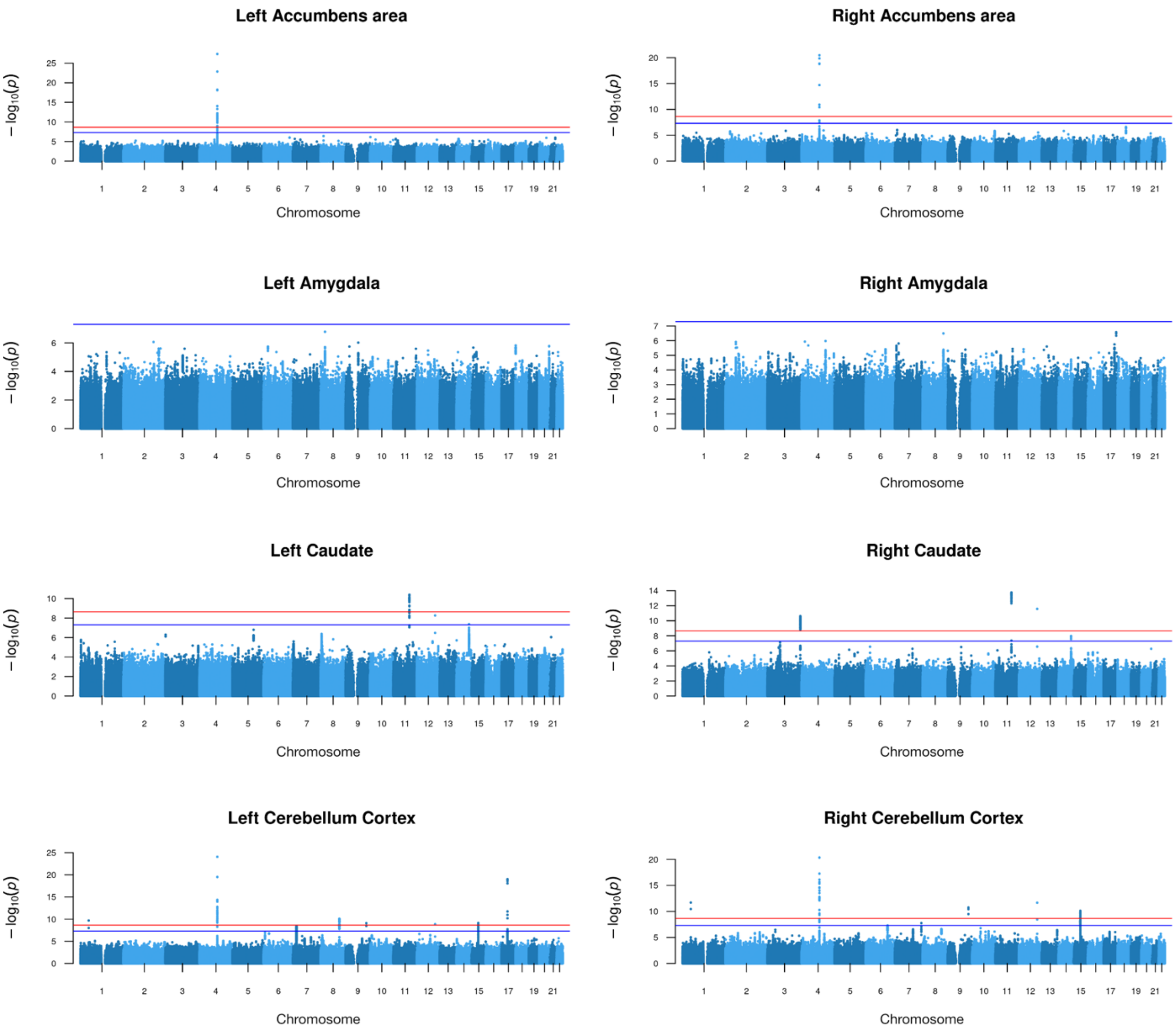

Fig. S2. MOSTest Results. This figure together with Fig. S3 and S4 display the Manhattan plots for all brain structures, each showing the results of the corresponding LBS MOSTest analysis. The chromosomal positions of the SNPs are indicated on the x-axis, and $-log_{10}$-scaled p-values on the y-axis. Blue and red lines indicate standard genome-wide significance (p = 5E-8) and genome-wide significance after Bonferroni correction for 22 brain structures (p = 2.27E-9), respectively. The Manhattan plot for brain stem analysis is shown in the main text.

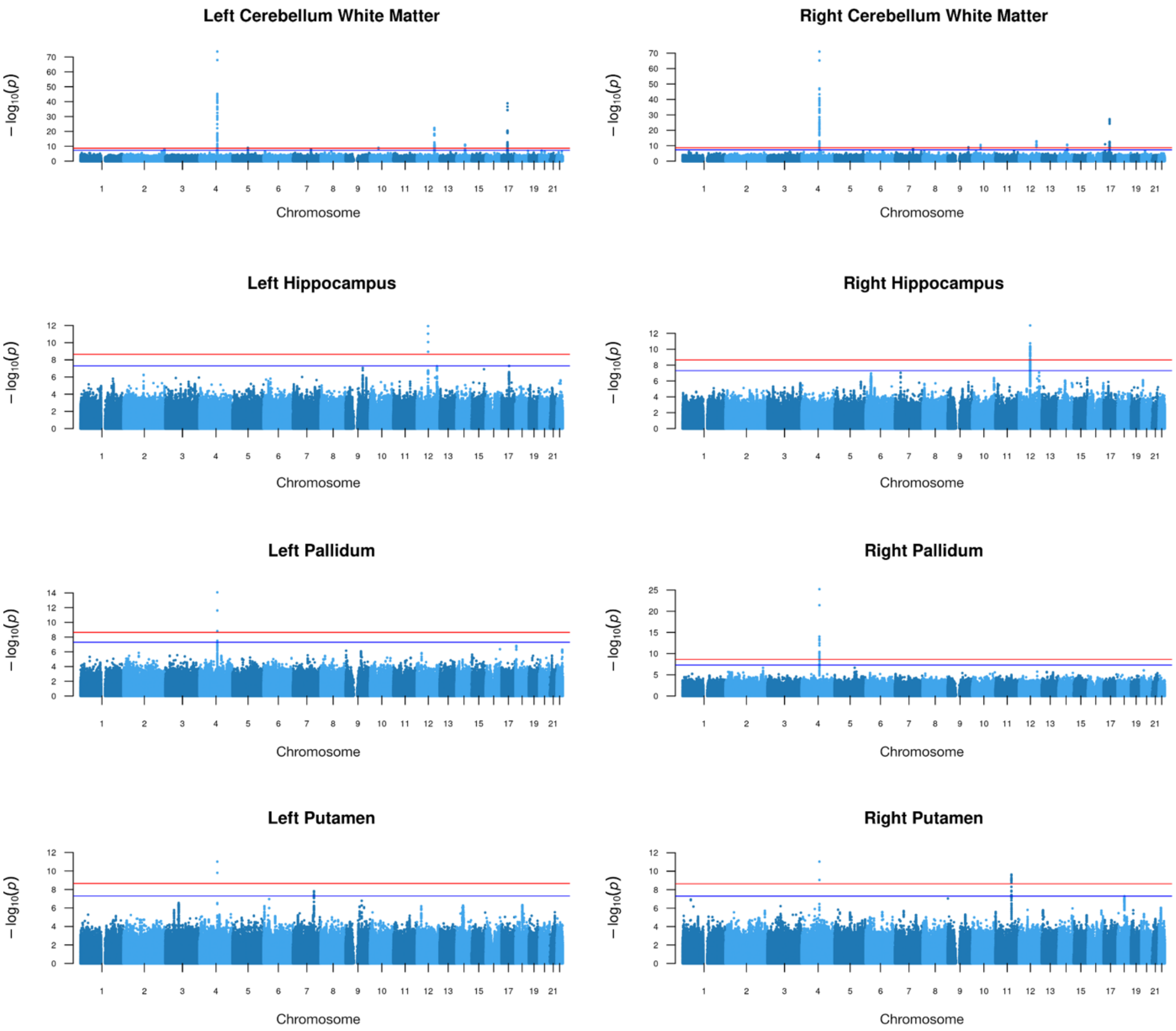

Fig. S3. MOSTest Results. See description of Fig. S2.

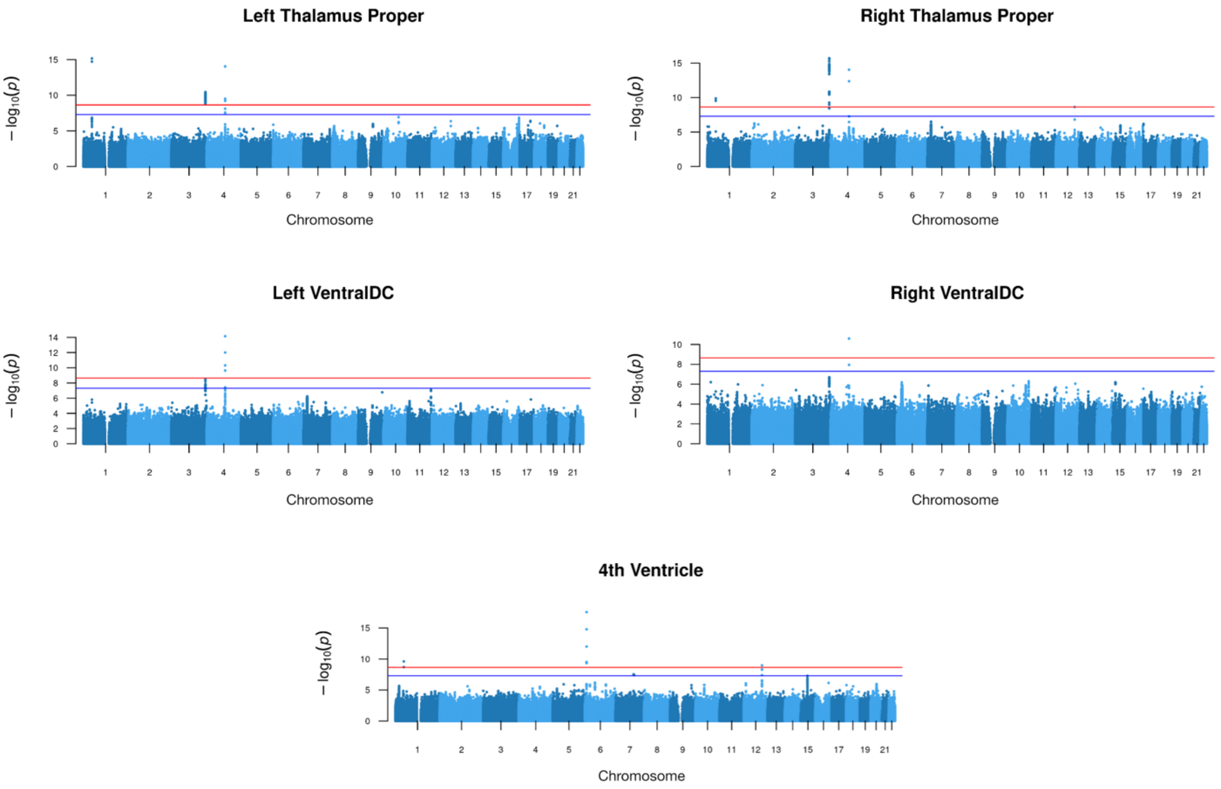

Fig. S4. MOSTest Results. See description of Fig. S2.

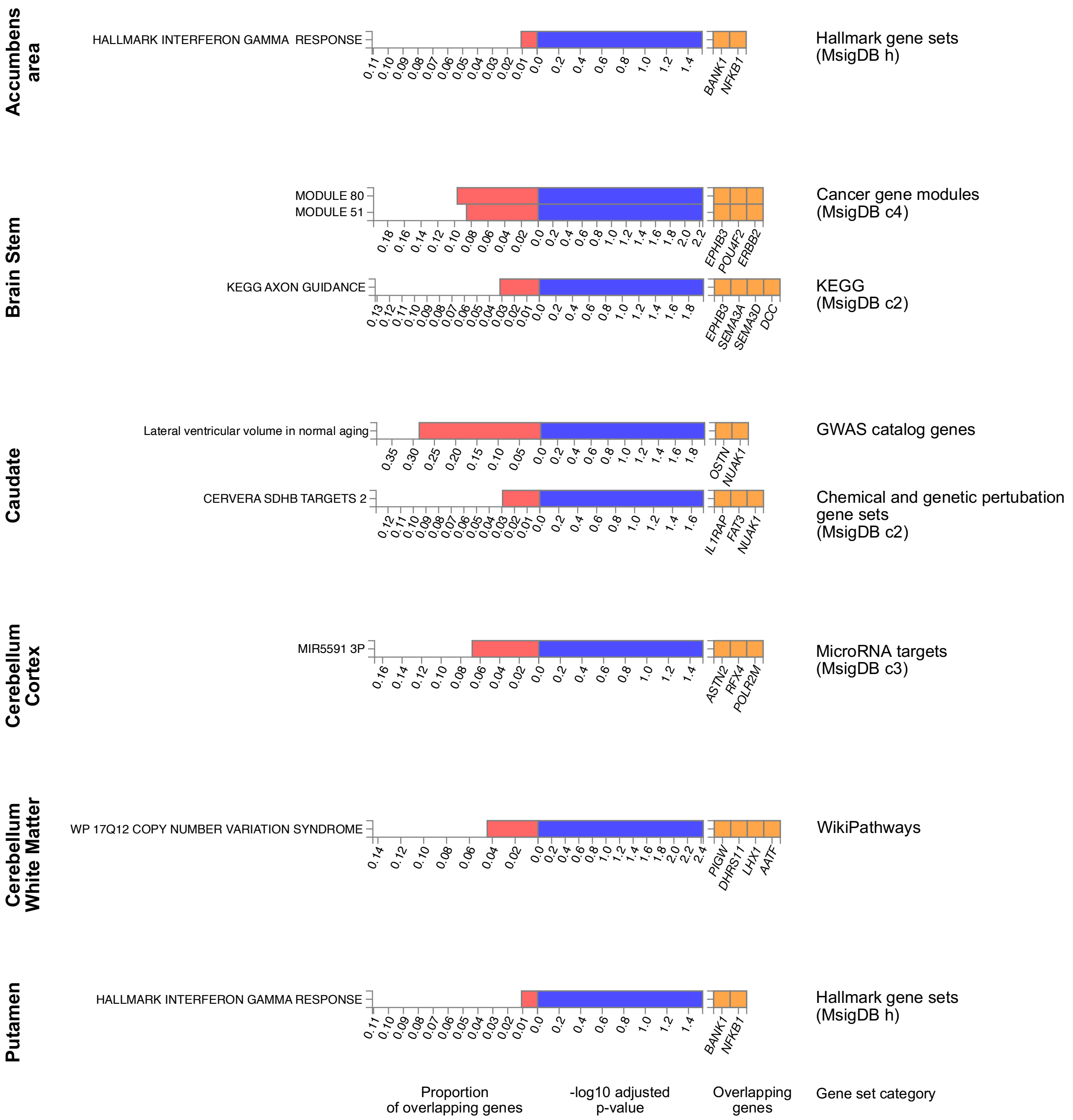

Fig. S5. Gene Set Enrichment. Significance of overrepresentation and overlap of prioritized genes with trait-associated and pathway-related gene sets using hypergeometric tests from FUMA's Gene2func method. P-values were corrected for multiple testing in each category by the Benjamini-Hochberg method.

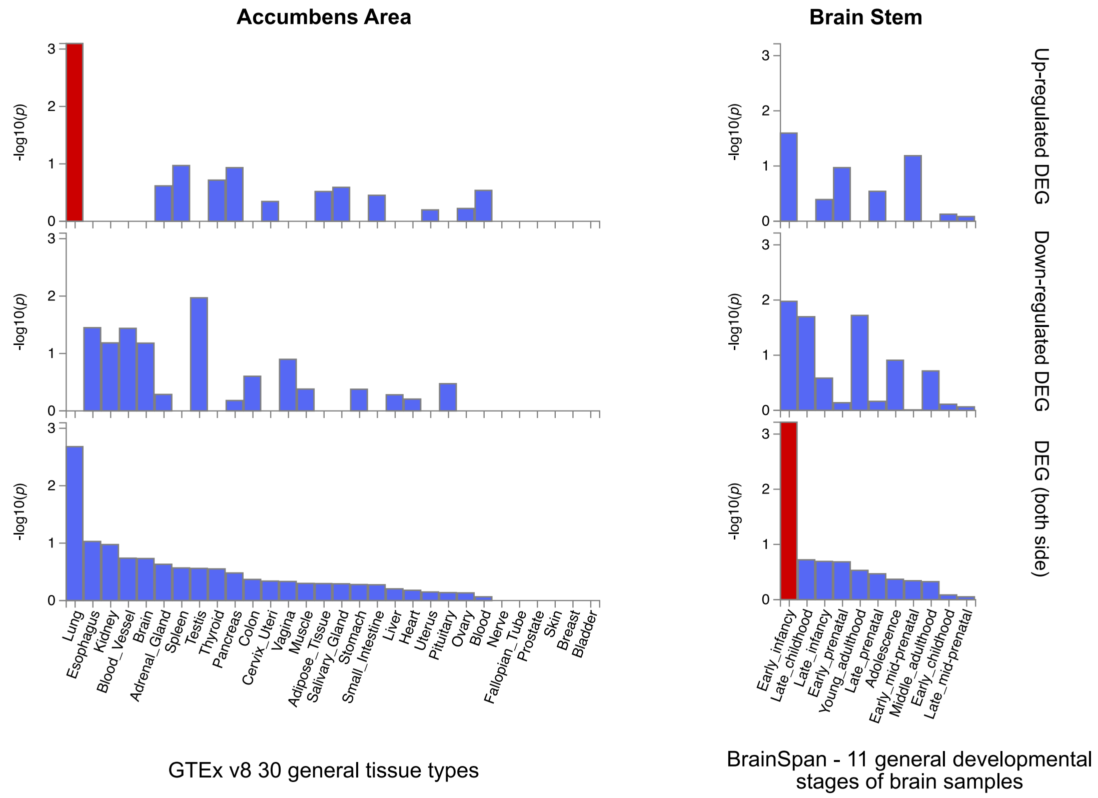

Fig. S6. Tissue Specificity of Prioritized Genes. Prioritized genes as resulting from MOSTest on LBS of accumbens area and brain stem were tested for enrichment in differentially expressed gene sets (x-axes) from GTEx v8 and BrainSpan using FUMA's Gene2func method. Y-axes indicate $-log_{10}$-scaled p-values. Significant enrichments (FDR-corrected) are indicated in red.

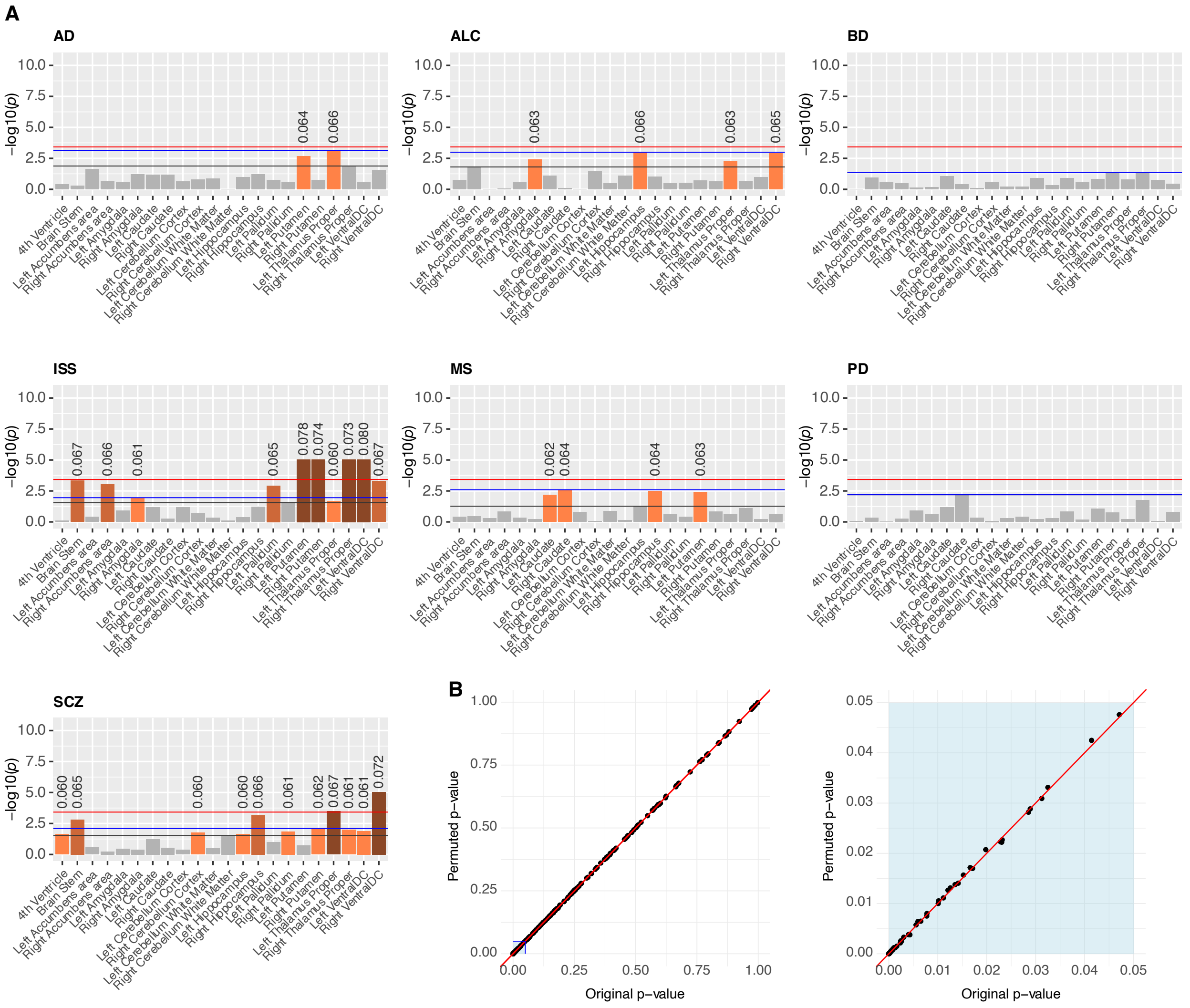

Fig. S7. Permutation Testing of CCA of Various PRS with Different Brain Structures. Permutation testing was performed for each polygenic risk score (PRS) and brain structure combination by shuffling the sample IDs in our eigenvalue matrix and re-calculating CCA 100,000 times. For combinations with a resulting permutation p-value of 0, we set the p-value to 9E-6 for plotting purposes. **A:** Bar charts show the $-log_{10}$-scaled p-values of the correlations, obtained by permutation testing, between PRS of Alzheimer's disease (AD), bipolar disorder (BD), ischemic stroke (ISS), multiple sclerosis (MS), Parkinson's disease (PD), schizophrenia (SCZ), and alcohol use disorder (ALC) and LBS of different brain structures at different significance levels: Bonferroni-corrected for 22 brain structures and 6 independent PRS (red line), FDR-corrected within each PRS (black line), FDR-corrected within each PRS + Bonferroni-corrected for 6 independent PRS (blue line). Above significant p-values, the correlation value is stated. **B:** Plots show p-values calculated by the chi-squared statistics (x-axis), as done in the main paper, and by permutation testing (y-axis). Right plot shows a zoomed area for values in [0,0.05]. The red line in both plots represents the identity line (x=y).

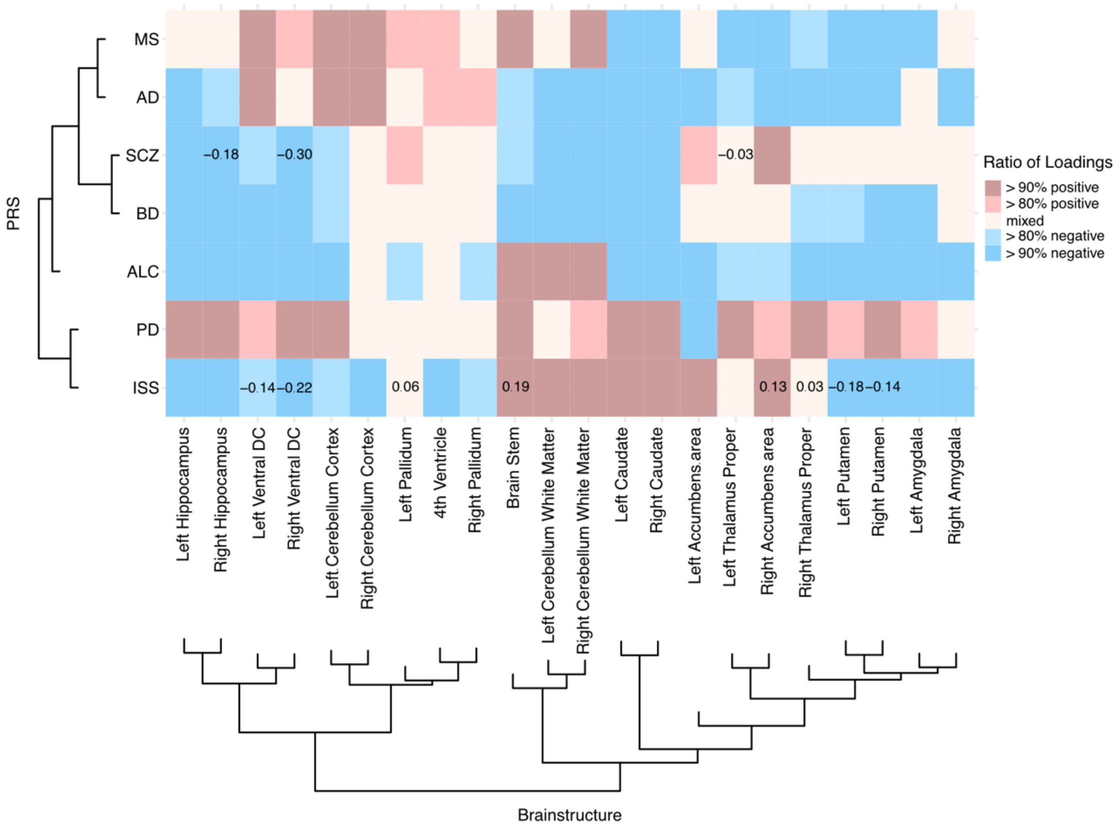

Fig. S8. Clustered Mean Loadings of CCA between PRS and the LBS. PRS (y-axis) were compared with the LBS of brain structures (x-axis) by canonical correlation analysis (CCA). For each pair of disease and brain structure the mean of the eigenvalue loadings was calculated. These means were used for hierarchical clustering. The heatmap shows for each PRS-LBS pair the proportion of positive (or negative) loadings in the CCA, with red color indicating the predominance of positive loadings and blue color the predominance of negative loadings. For significant CCA results the mean loading is stated in the respective cell. Abbreviations of PRS as in Fig. S7.

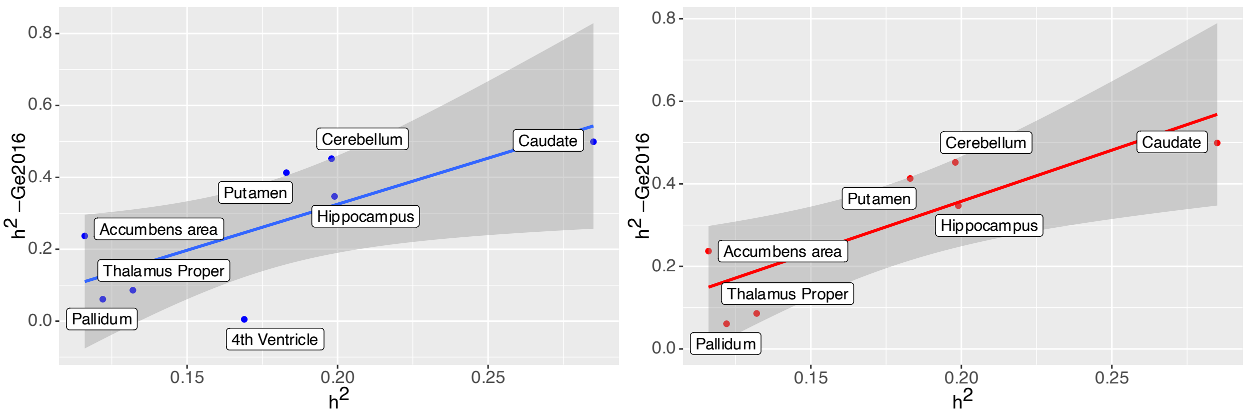

Fig. S9. Comparison of Heritability Estimates. The heritabilities of brain structures, as determined by (*6*), were compared to the heritabilities derived in the present study. For bilateral structures and combined regions, such as cerebellum, a combined heritability measure was calculated (see Methods of main text). The left plot shows all structures for which heritabilities are available from both studies and a linear fit with a 95% confidence interval. Since the result for the 4th ventricle might be an outlier in (*6*), the comparison was repeated without that structure as shown in the right plot.

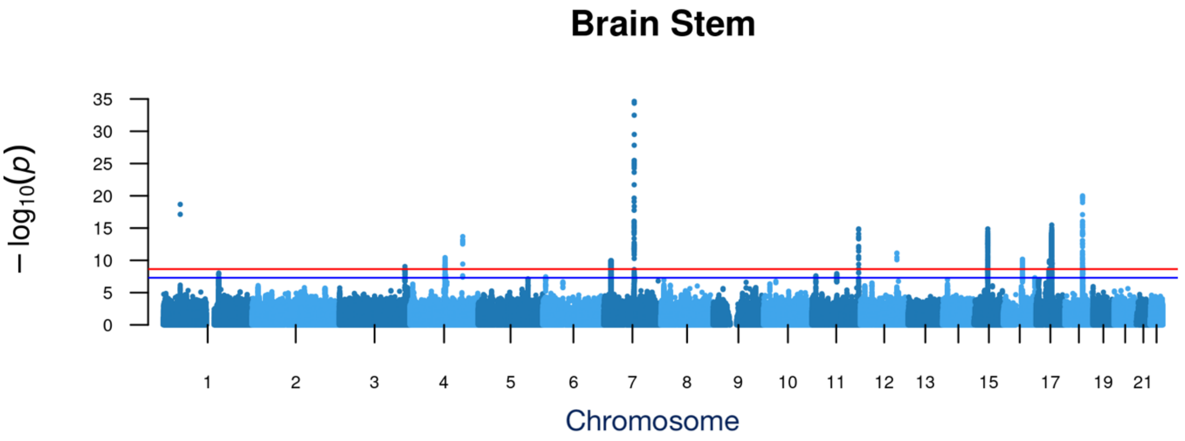

Fig. S10. GWAS of Brain Stem with Height as Additional Covariate. Manhattan plot of MOSTest results for brain stem LBS with height as additional covariate. Chromosomal locations of the SNPs are indicated on the x-axis and $-log_{10}$-scaled p-values on the y-axis. Blue and red lines indicate standard genome-wide significance (p = 5E-8) and genome-wide significance after Bonferroni correction for 22 brain structures (p = 2.27E-9), respectively.

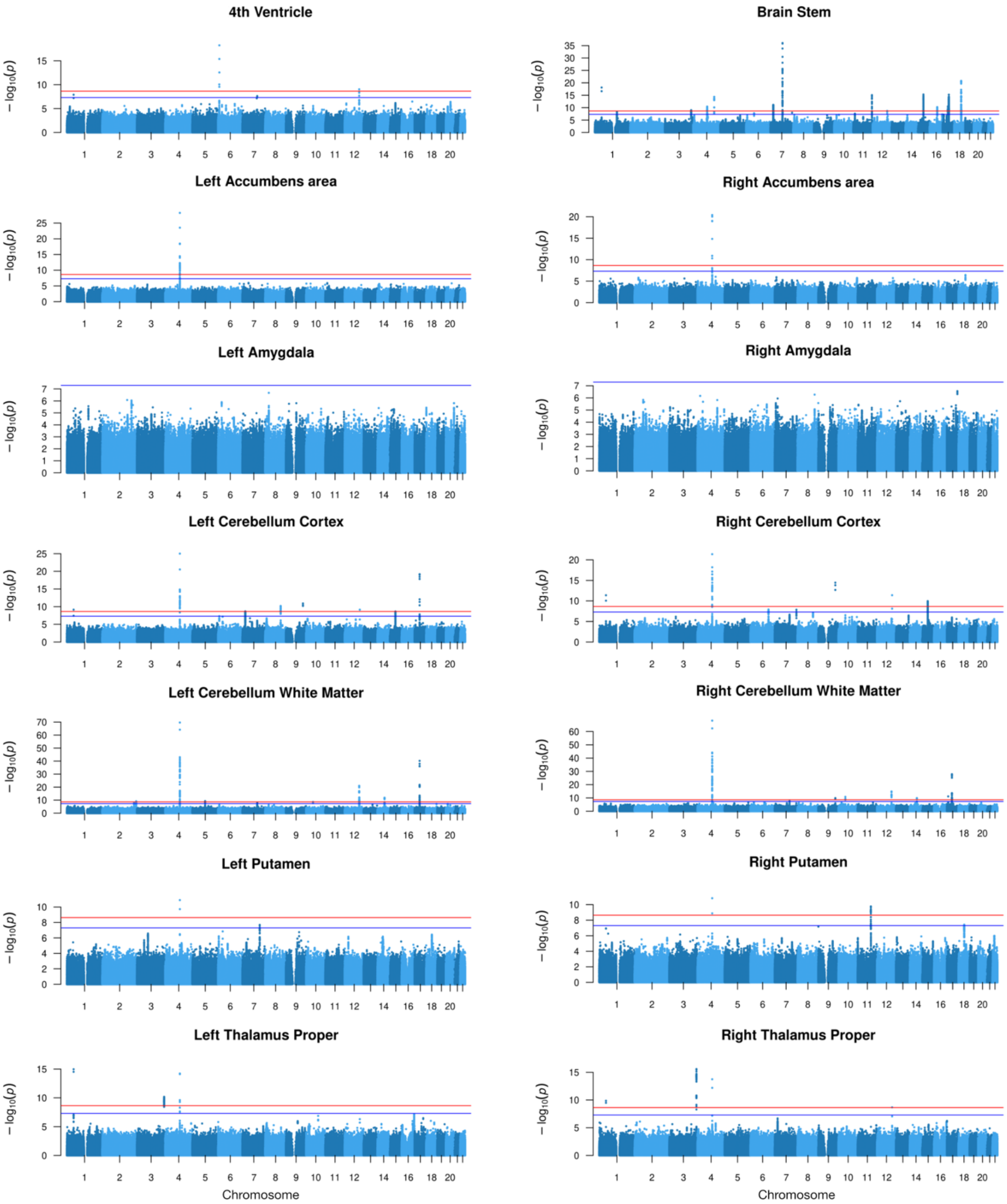

Fig. S11. MOSTest Results After Outlier Removal. Figure shows Manhattan plots of the brain structures which have been reanalyzed: 4th ventricle (124 removed outliers), brain stem (90), left accumbens area (163), left amygdala (85), left cerebellum cortex (119), left cerebellum white matter (116), left putamen (20), left thalamus proper (22), right accumbens area (43), right amygdala (54), right cerebellum cortex (106), right cerebellum white matter (100), right putamen (21), and right thalamus proper (27). Further information, see description of Fig. S2.

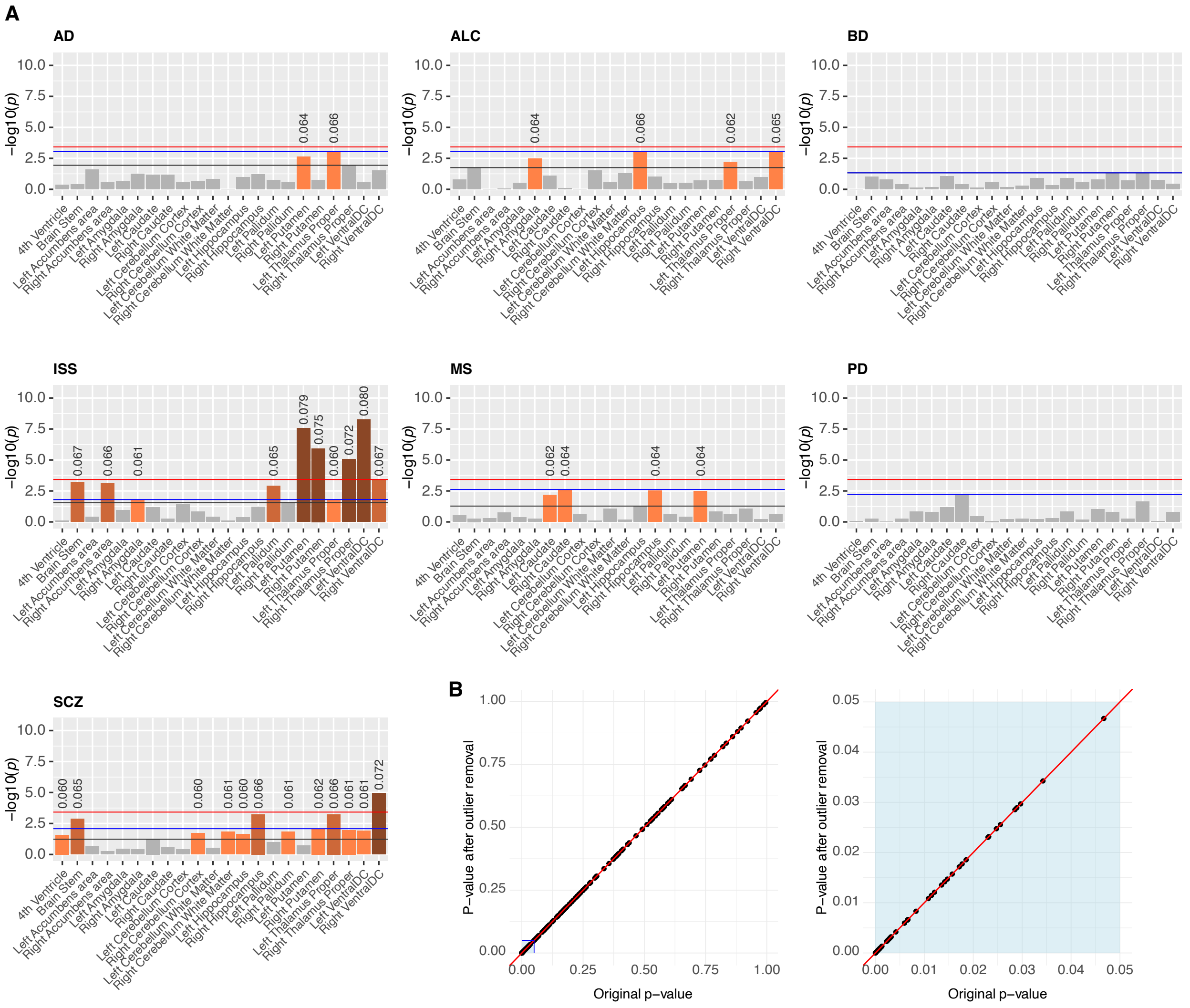

Fig. S12. Reanalysis of CCA of Various PRS with Different Brain Structures After Outlier Removal. A: Bar charts show the $-log_{10}$-scaled p-values of the correlations between PRS and LBS of different brain structures at different significance levels after outlier removal: Bonferroni-corrected for 22 brain structures and 6 independent PRS (red line), FDR-corrected within each PRS (black line), FDR-corrected within each PRS + Bonferroni-corrected for 6 independent PRS (blue line). Above significant p-values, the correlation value is stated. Abbreviations of PRS as in Fig. S7. **B:** Plots show p-values calculated before (x-axis) and after outlier removal (y-axis). Right plot shows a zoomed area for values in [0,0.05]. The red line in both plots represents the identity line (x=y).

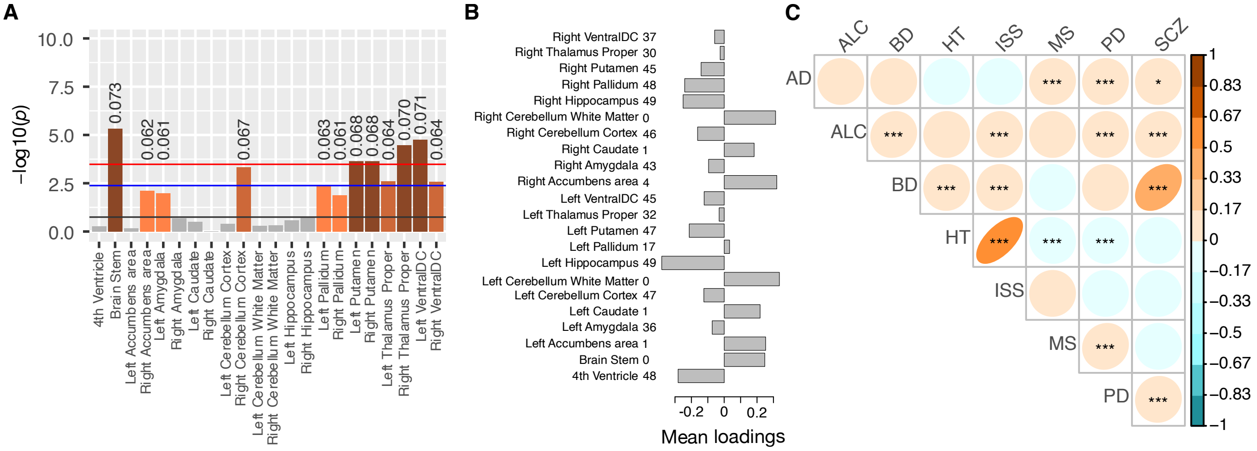

Fig. S13. CCA Results for Hypertension PRS. The PRS of hypertension (HT, extracted from data field 26244 of UKB) had a Pearson correlation coefficient of 0.64 ($-log(p) = 5210.3$) with the PRS of ischemic stroke (ISS). Therefore, the results of the canonical correlation analysis (CCA) between HT PRS and the LBS of different brain structures were quite similar to those of the analogous CCA results for ISS PRS which are shown in the main text. **A:** Bar chart showing the p-values that resulted from CCA of HT PRS and brain structure LBS. Significance thresholds of increasing stringency are analogous to those in Fig. 7 of the main text, i.e., FDR-corrected for the set of brain structures (black line), FDR-corrected for the set of brain structures combined with Bonferroni-correction for 7 independent PRS (blue line), and Bonferroni-correction for 22 brain structures and 7 independent PRS (red line). **B**: Means of the loadings of the eigenvalues which were produced by CCA of HT PRS and the LBS of different brain structures. The number of negative loadings is stated after each structure's name. **C**: Coefficients of Pearson correlations among different disease PRS (abbreviations see Fig. S7) and their significances with $p < 0.05$ indicated by "*", $p < 0.01$ by "**", and Bonferroni-adjusted $p < 0.05/28$ by "***".

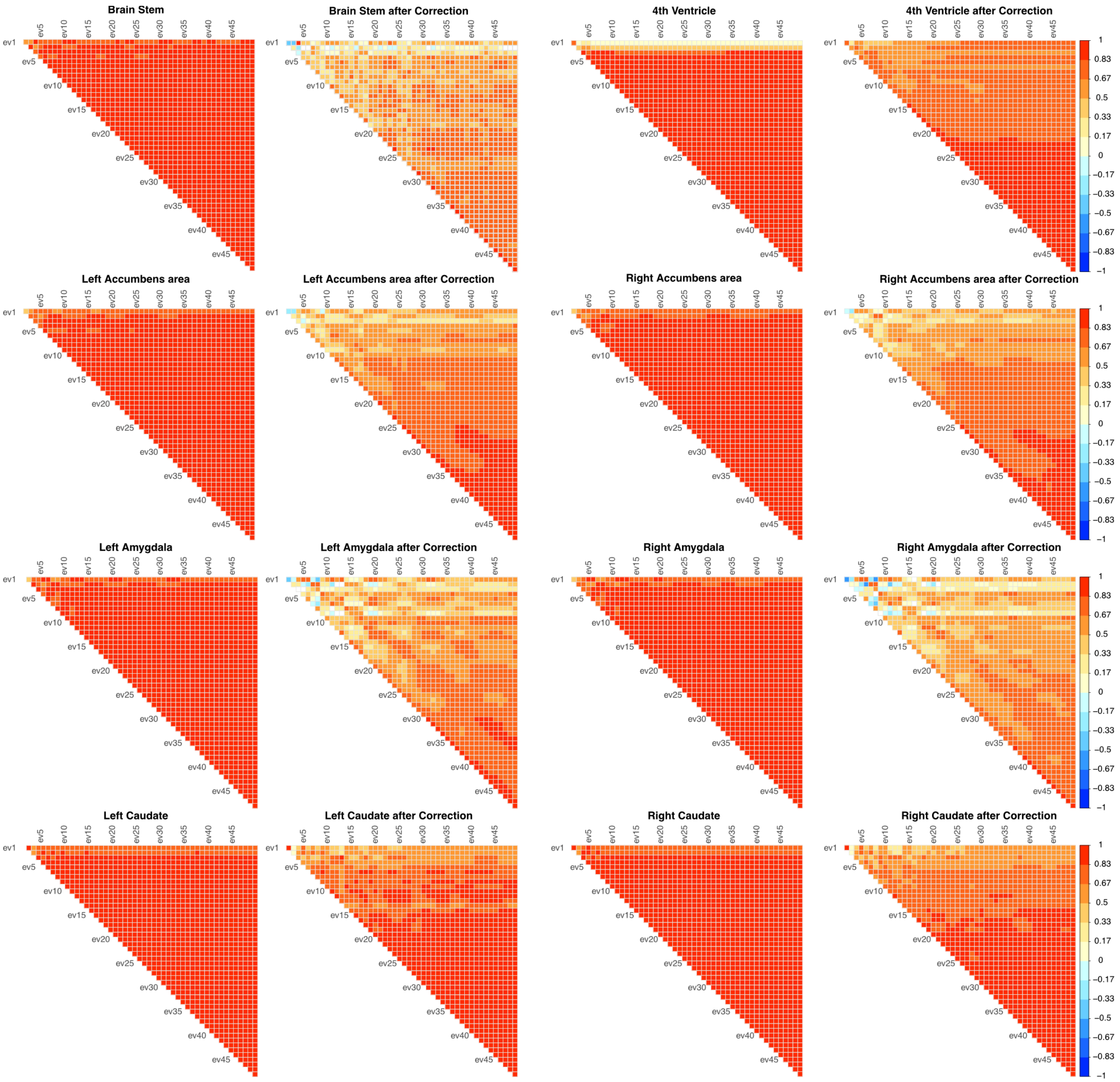

Fig. S14. Phenotype Correlation. This figure, as well as Fig. S15 and S16, show for each brain structure the Pearson correlation coefficients between eigenvalues before (raw) and after volume normalization and regressing on covariates. White cells indicate non-significant ($p \geq 0.05/49$) correlations.

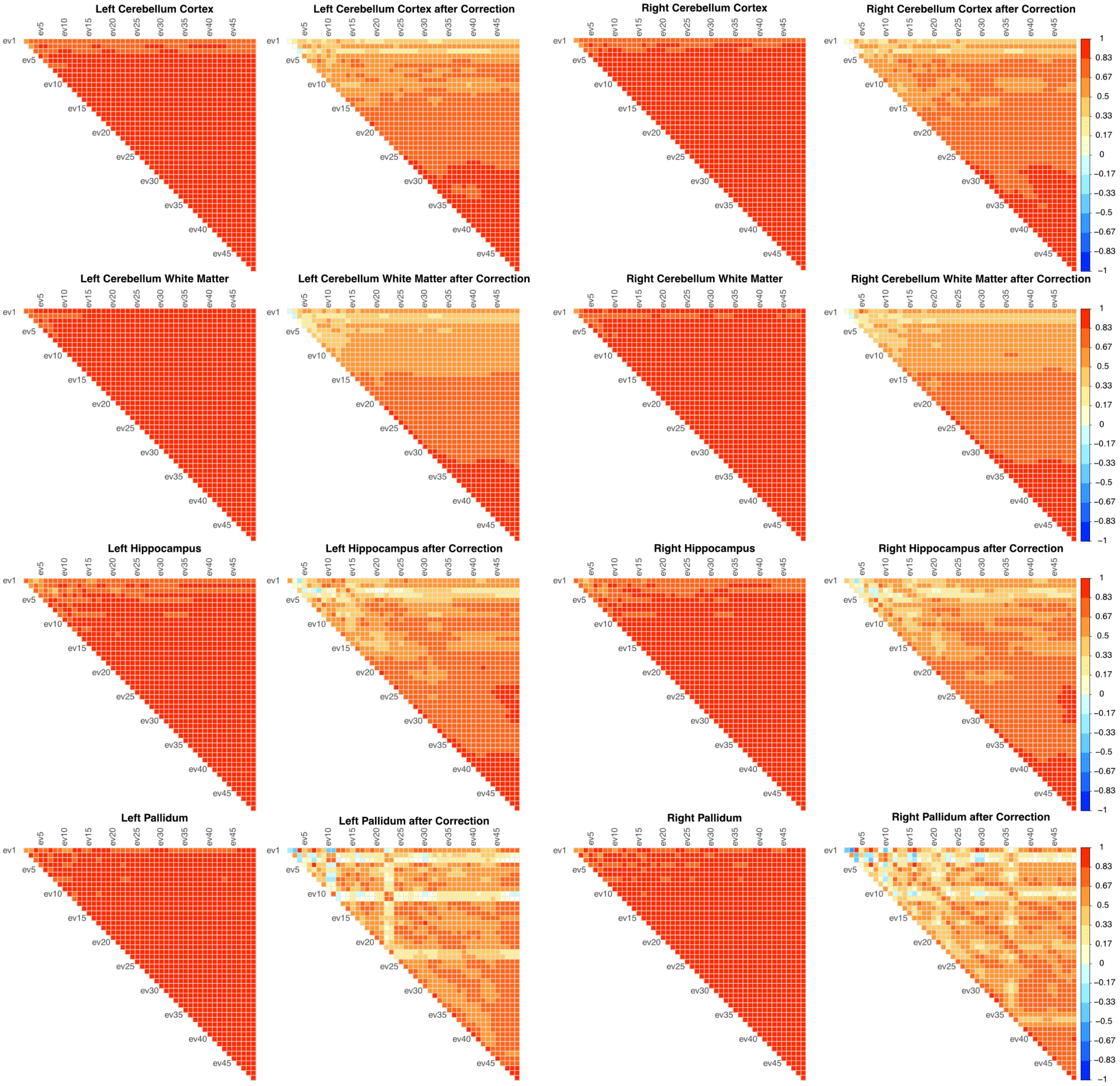

Fig. S15. Phenotype Correlation. See description of Fig. S14.

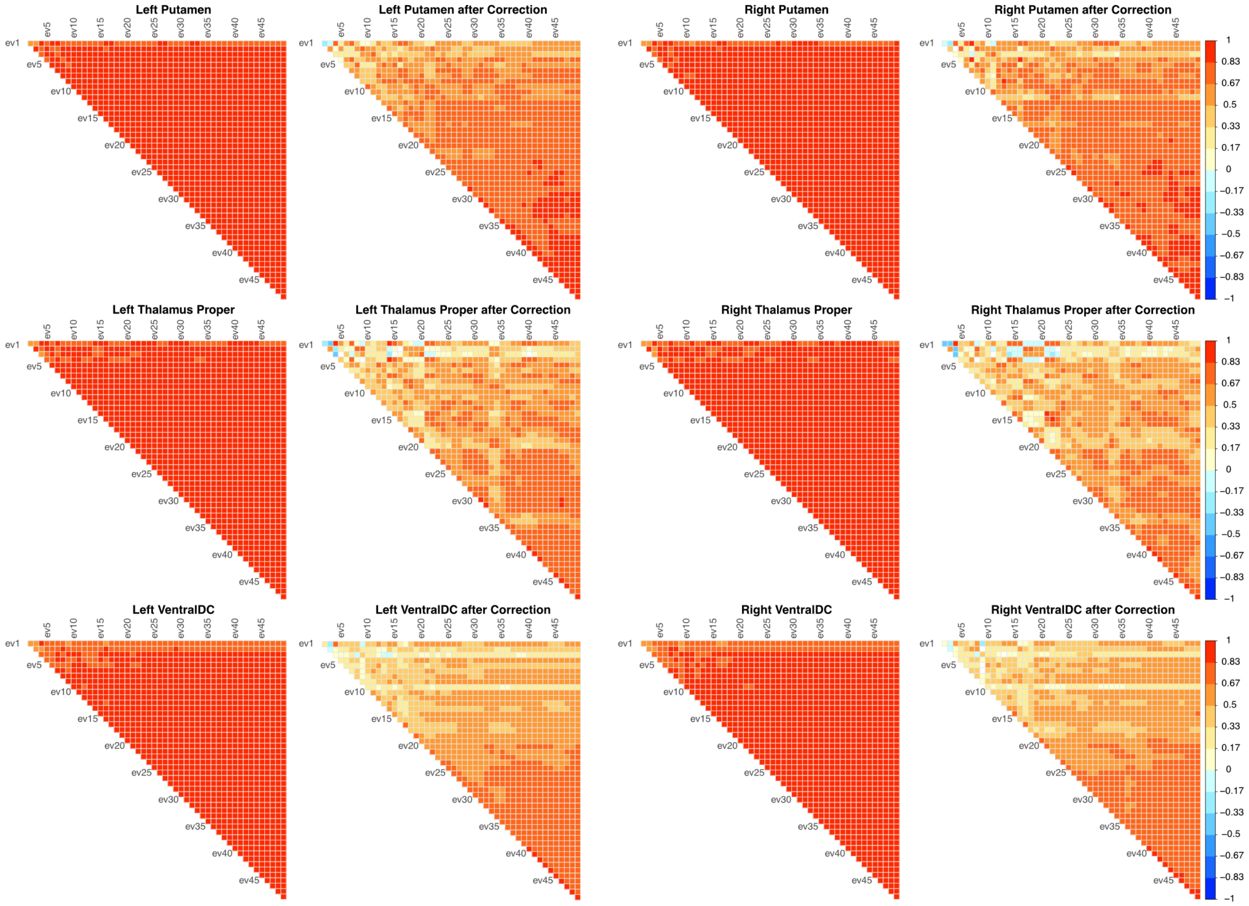

Fig. S16. Phenotype Correlation. See description of Fig. S14.

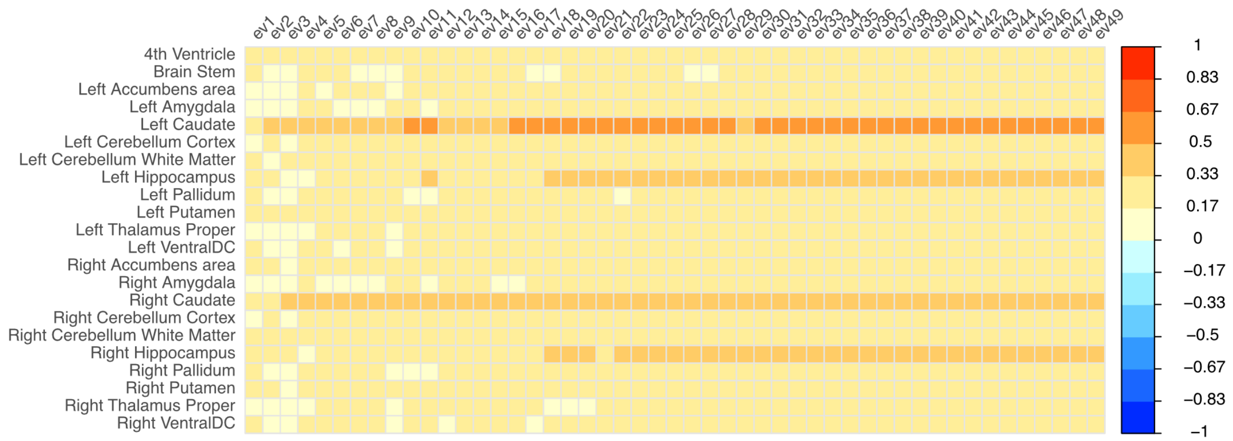

Fig. S17. Correlation Between LBS and Volume. Coefficients of Pearson correlation between the volumes of brain structures (y-axis) and their normalized and residualized Laplace-Beltrami eigenvalues.

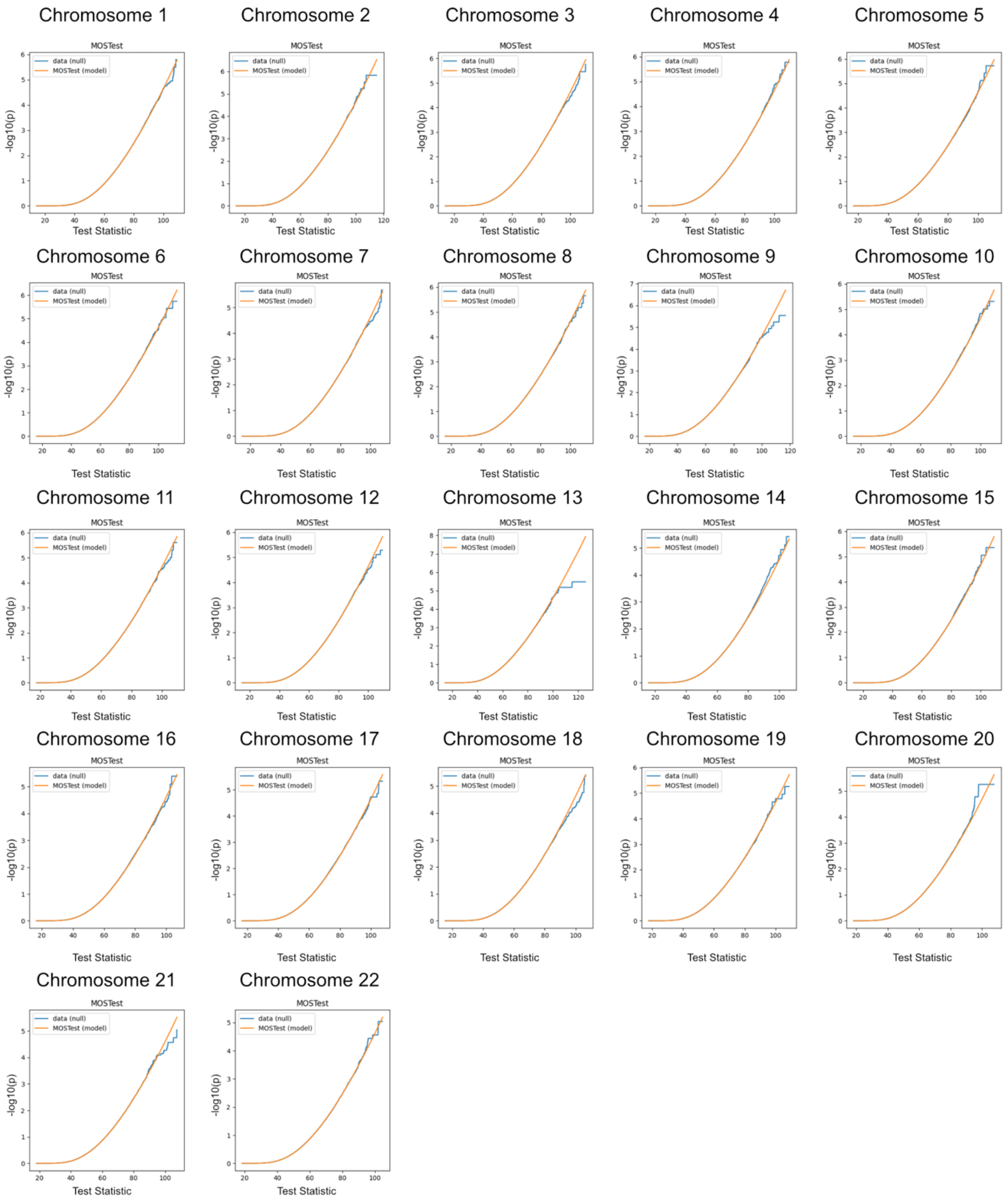

Fig. S18. Chromosome-wise Comparison of Empirical and Analytical MOSTest Results Under the Null Hypothesis for Brain Stem. Each subplot covers one chromosome and shows the value of MOSTest test statistic on the x-axis. The MOSTest model curve shows theoretical p-values calculated from a gamma function fitted to the test statistic (orange). The blue curve shows the empirical distribution of the test statistic. Coincidence indicates a uniform distribution of p-values under the null and a well-controlled type I error.

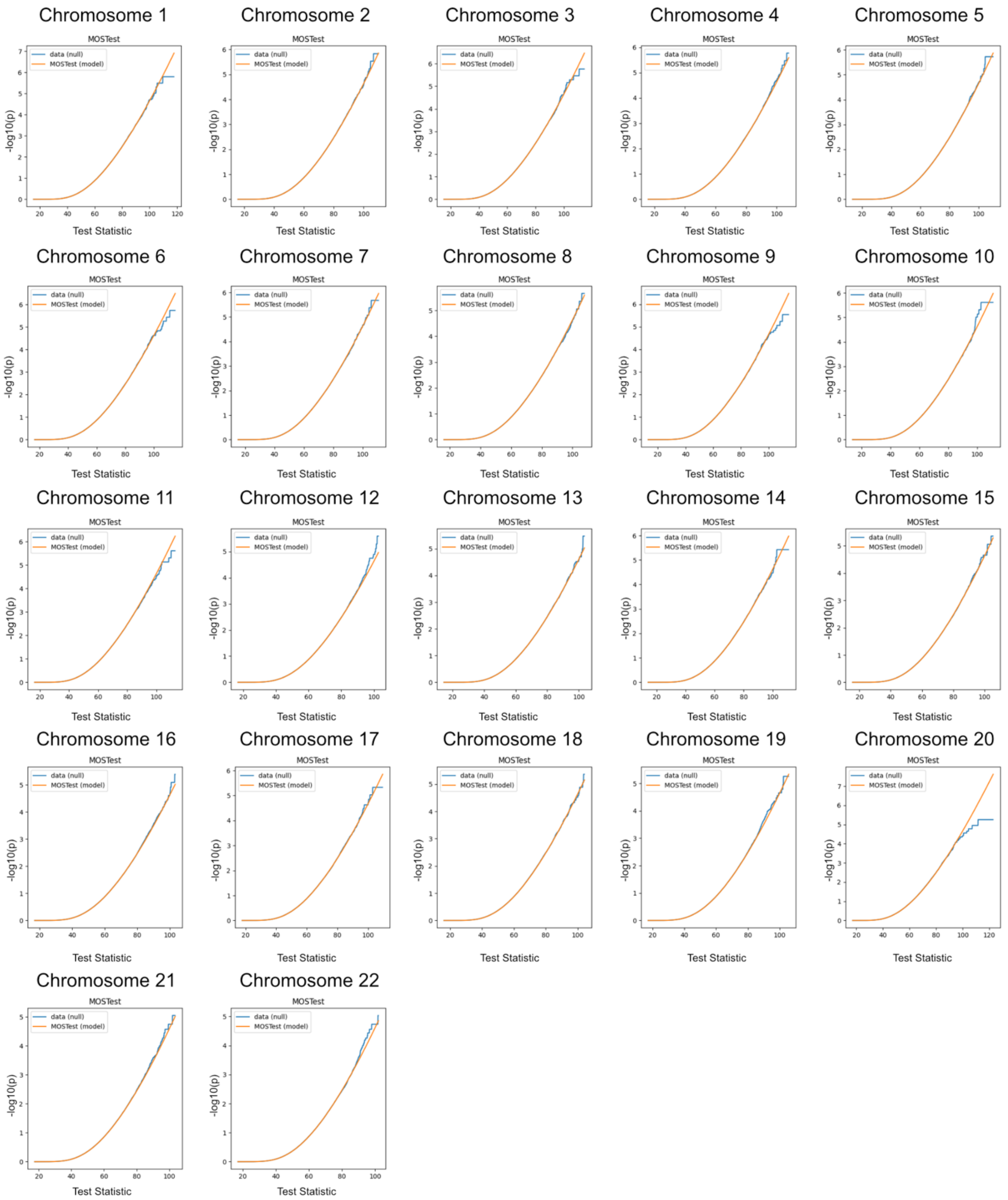

Fig. S19. Chromosome-wise Comparison of Empirical and Analytical MOSTest Results Under the Null Hypothesis for left Accumbens Area. See description of Fig. S18.

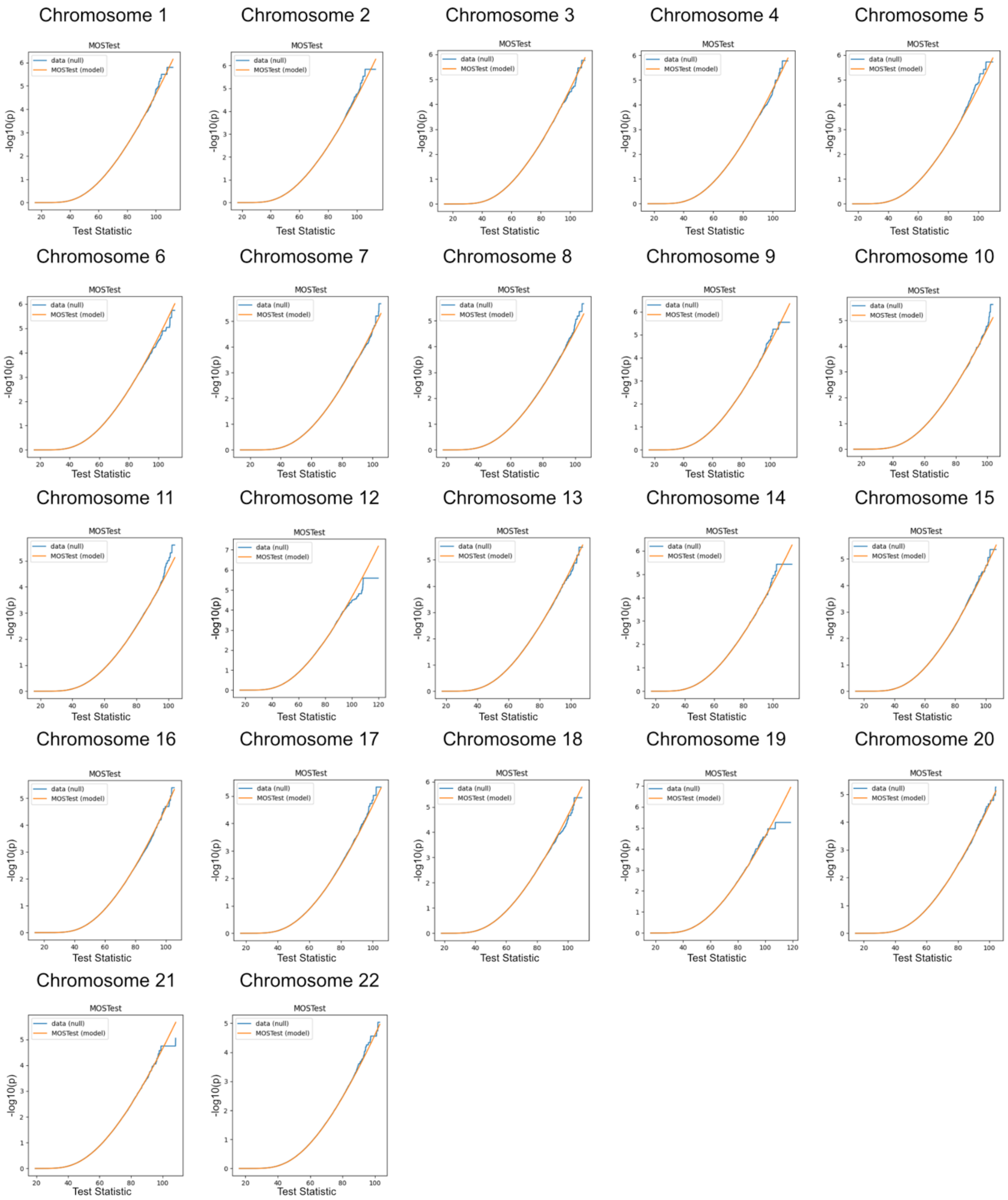

Fig. S20. Chromosome-wise Comparison of Empirical and Analytical MOSTest Results Under the Null Hypothesis for right Accumbens Area. See description of Fig. S18.

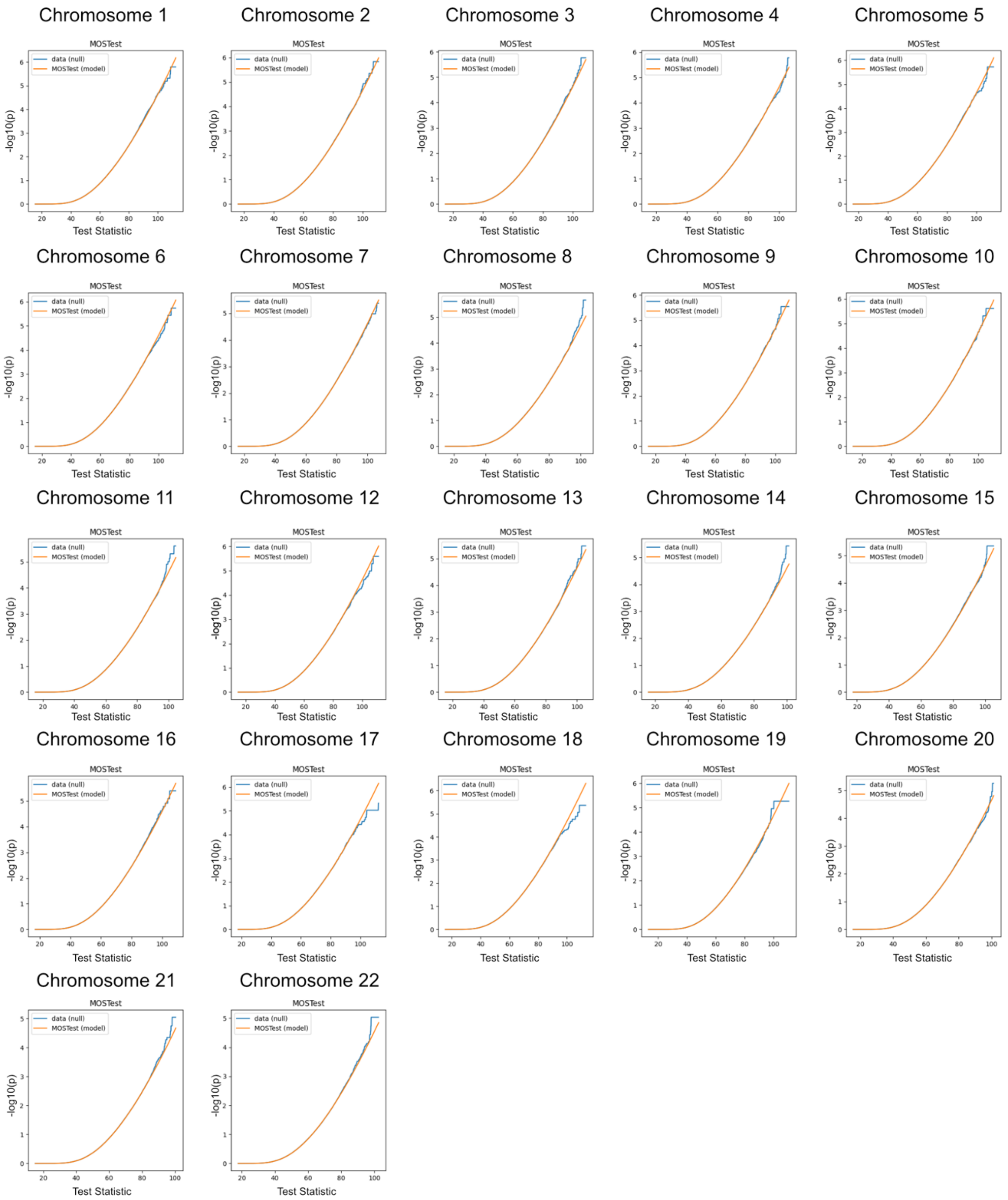

Fig. S21. Chromosome-wise Comparison of Empirical and Analytical MOSTest Results Under the Null Hypothesis for left Amygdala. See description of Fig. S18.

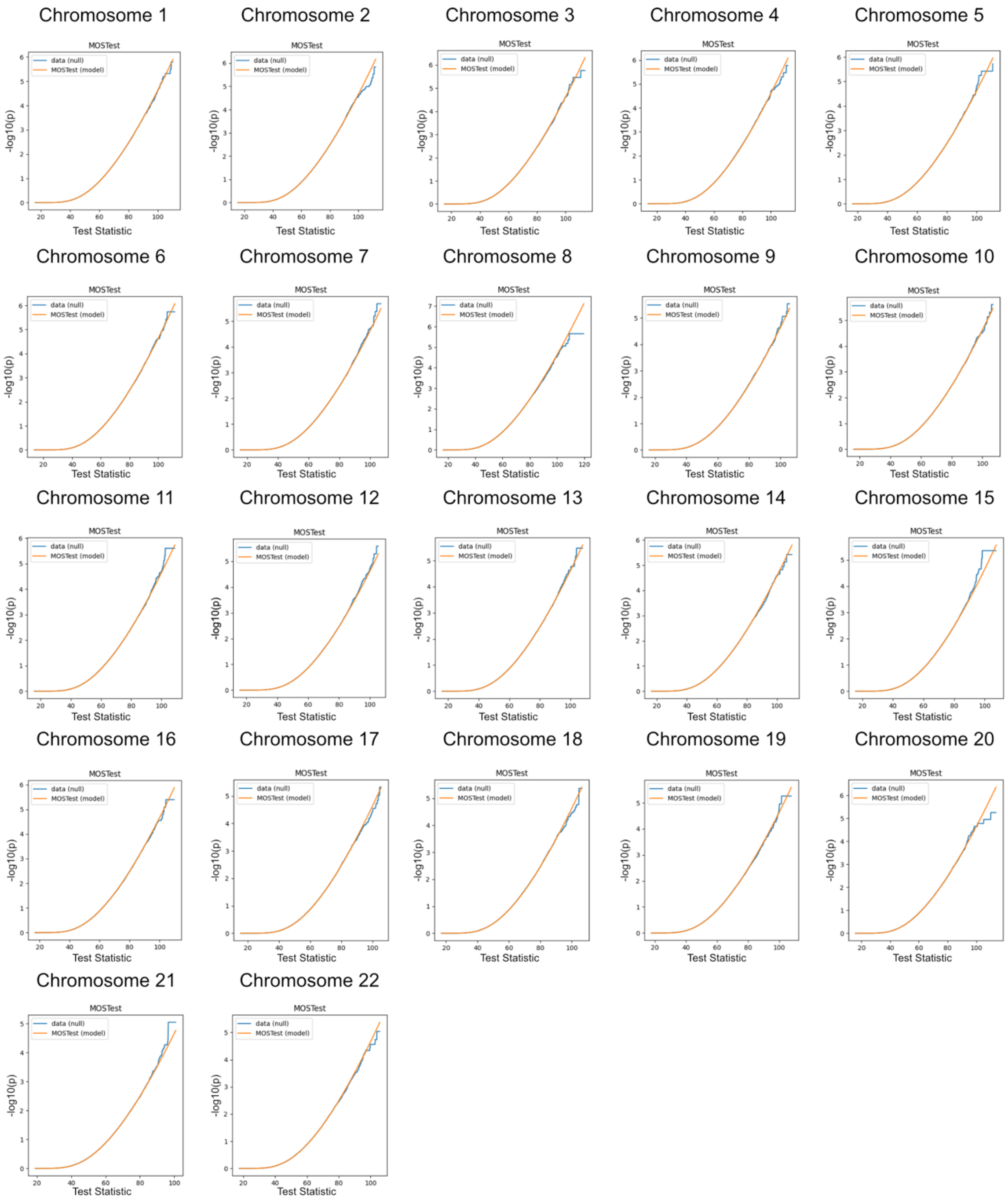

Fig. S22. Chromosome-wise Comparison of Empirical and Analytical MOSTest Results Under the Null Hypothesis for right Amygdala. See description of Fig. S18.

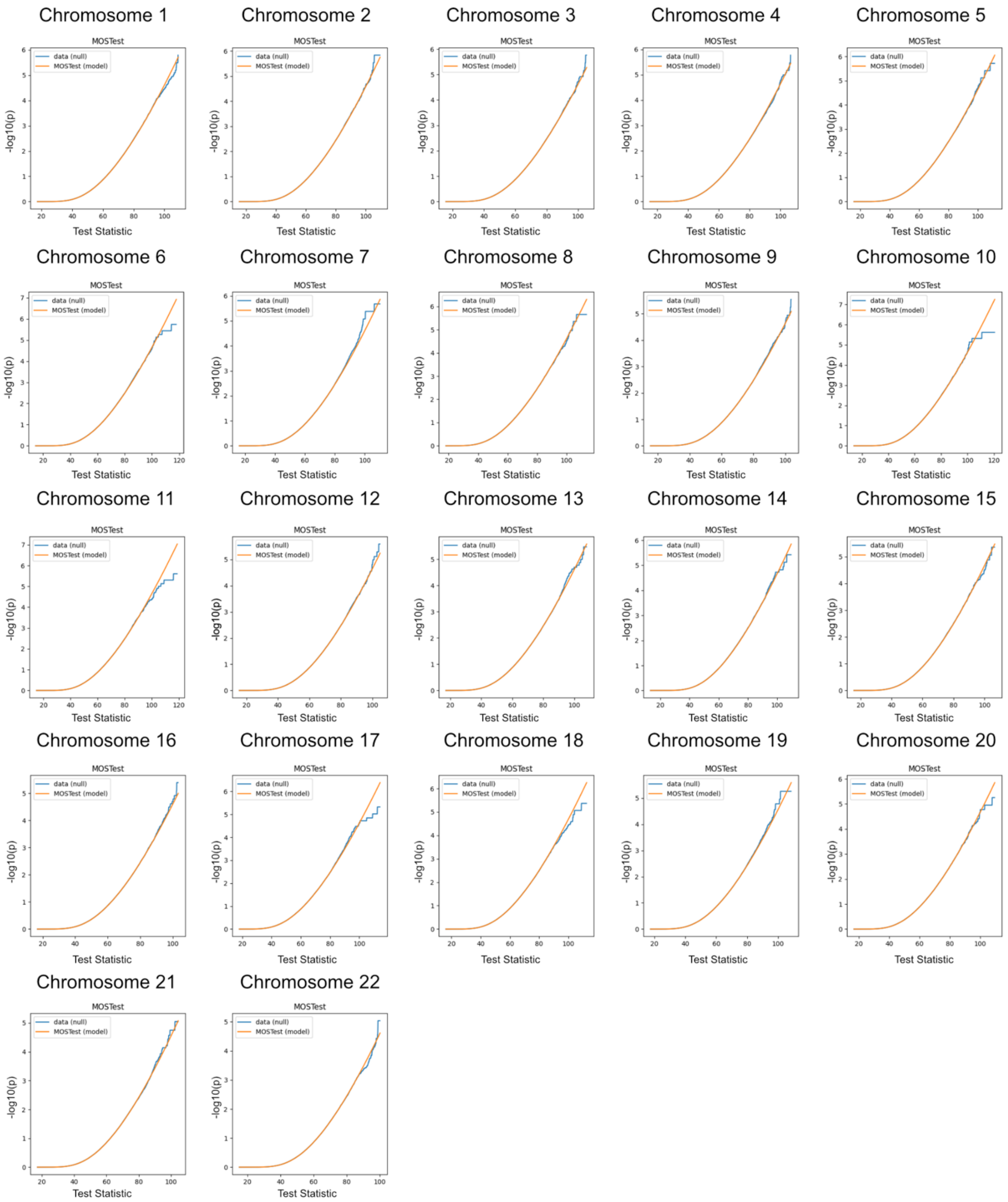

Fig. S23. Chromosome-wise Comparison of Empirical and Analytical MOSTest Results Under the Null Hypothesis for left Caudate. See description of Fig. S18.

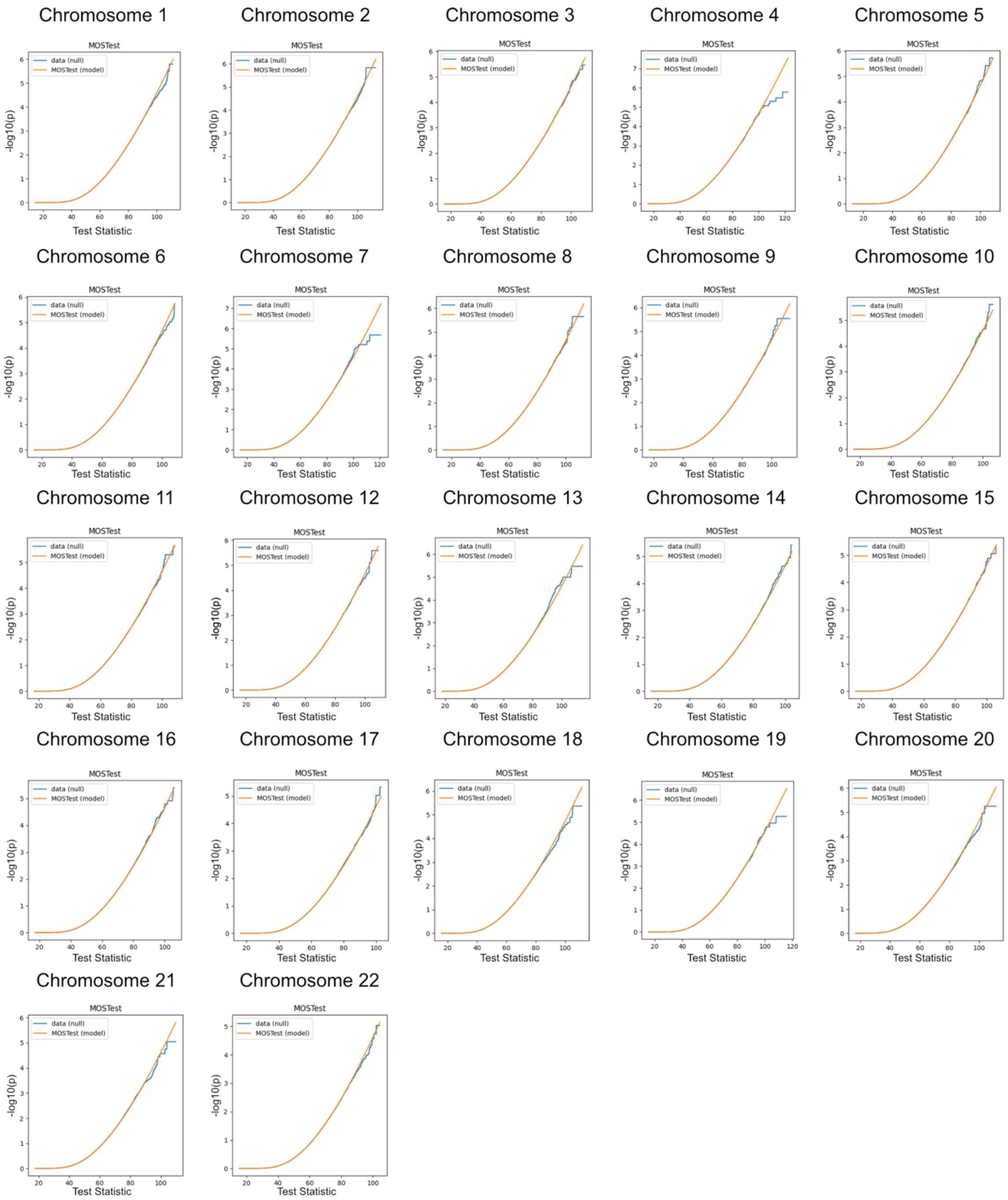

Fig. S24. Chromosome-wise Comparison of Empirical and Analytical MOSTest Results Under the Null Hypothesis for right Caudate. See description of Fig. S18.

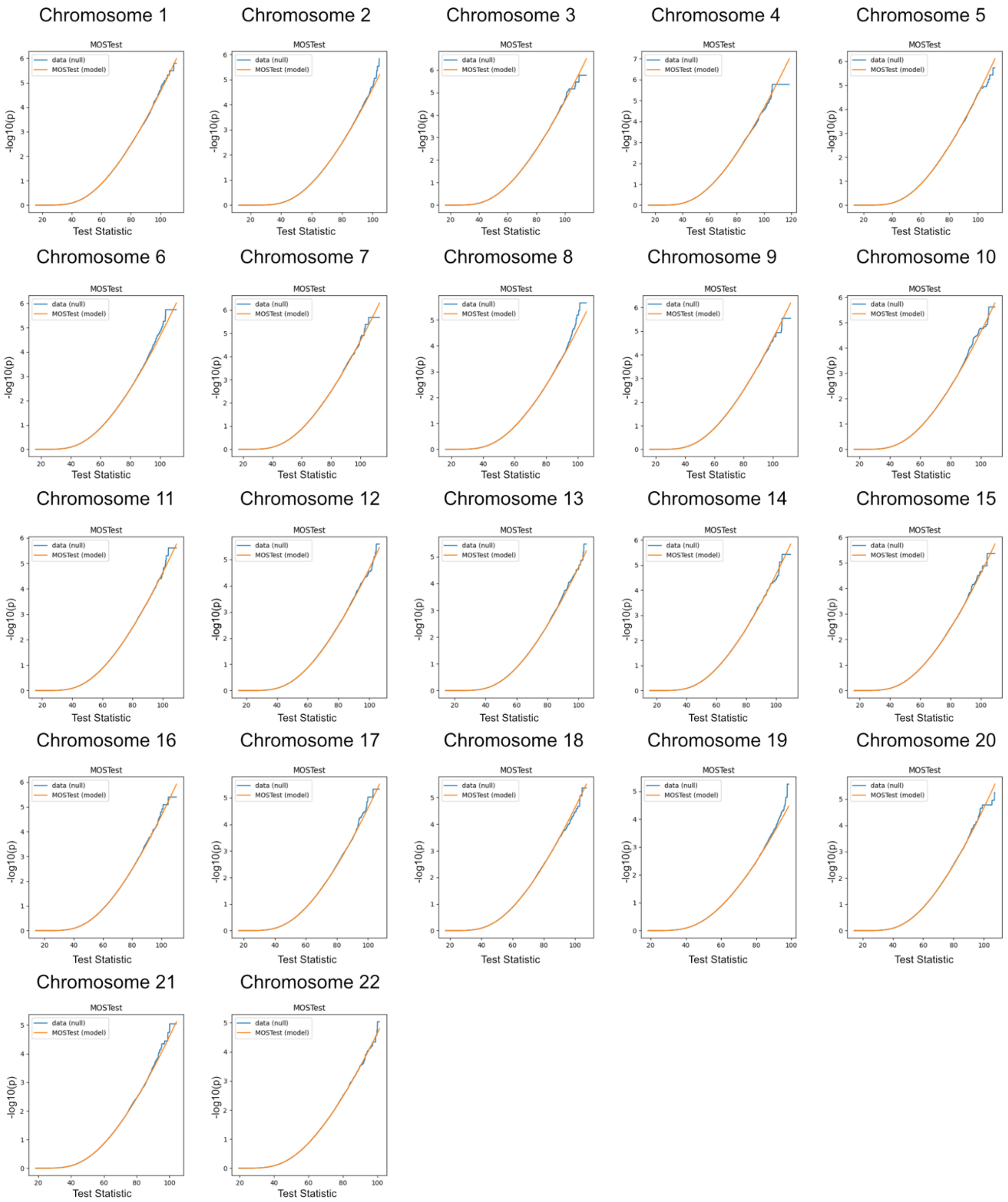

Fig. S25. Chromosome-wise Comparison of Empirical and Analytical MOSTest Results Under the Null Hypothesis for left Cerebellum Cortex. See description of Fig. S18.

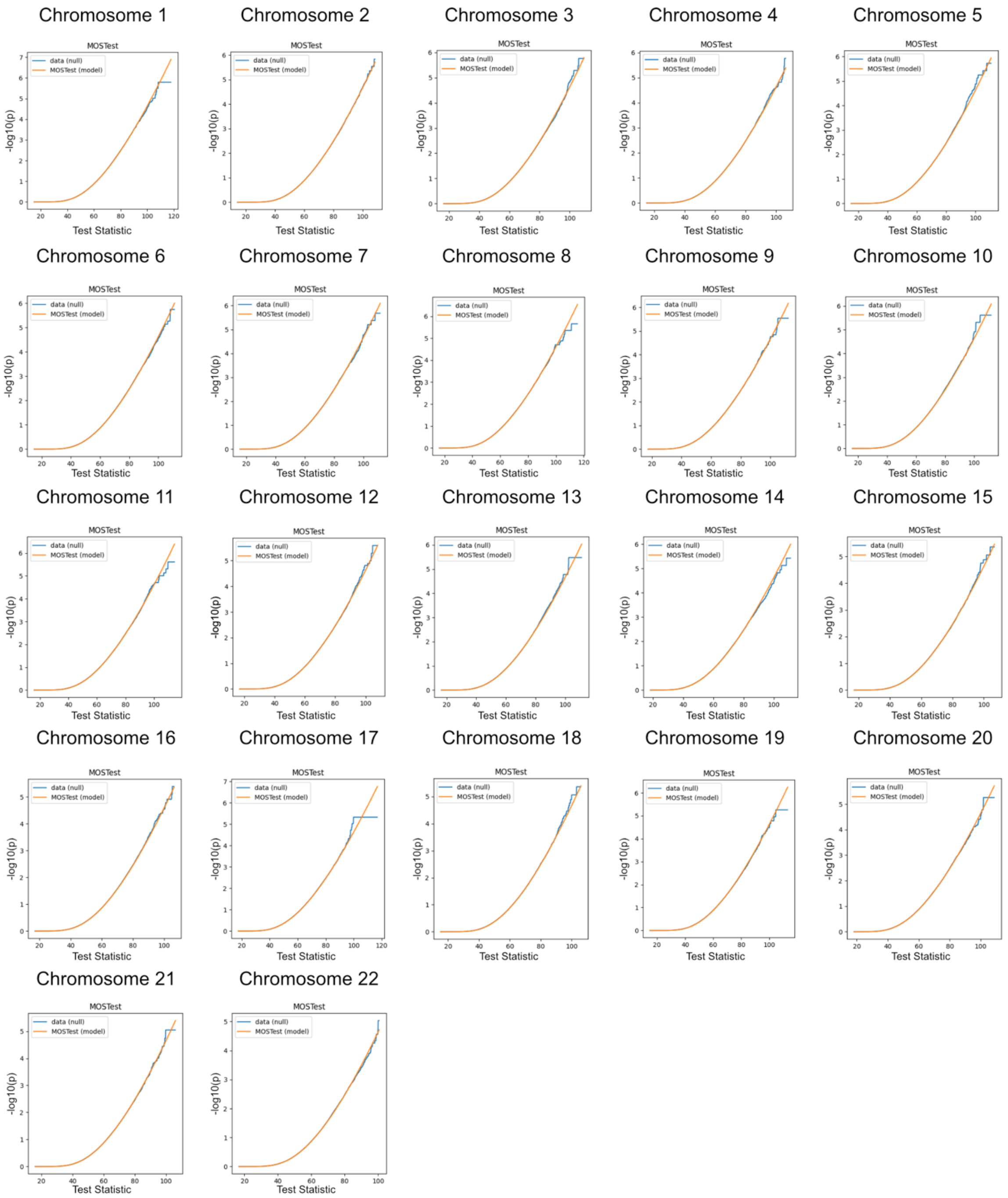

Fig. S26. Chromosome-wise Comparison of Empirical and Analytical MOSTest Results Under the Null Hypothesis for right Cerebellum Cortex. See description of Fig. S18.

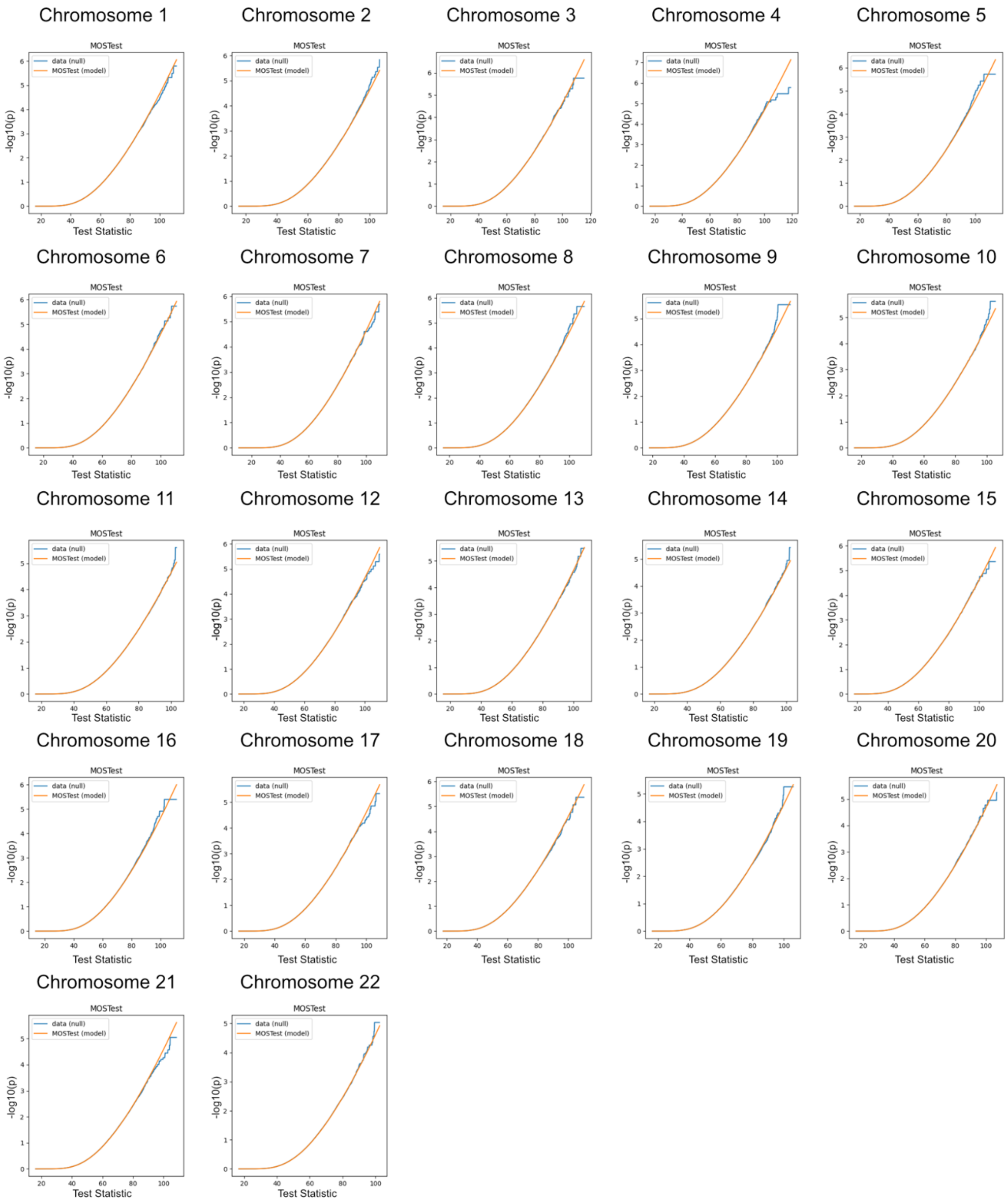

Fig. S27. Chromosome-wise Comparison of Empirical and Analytical MOSTest Results Under the Null Hypothesis for left Cerebellum White Matter. See description of Fig. S18.

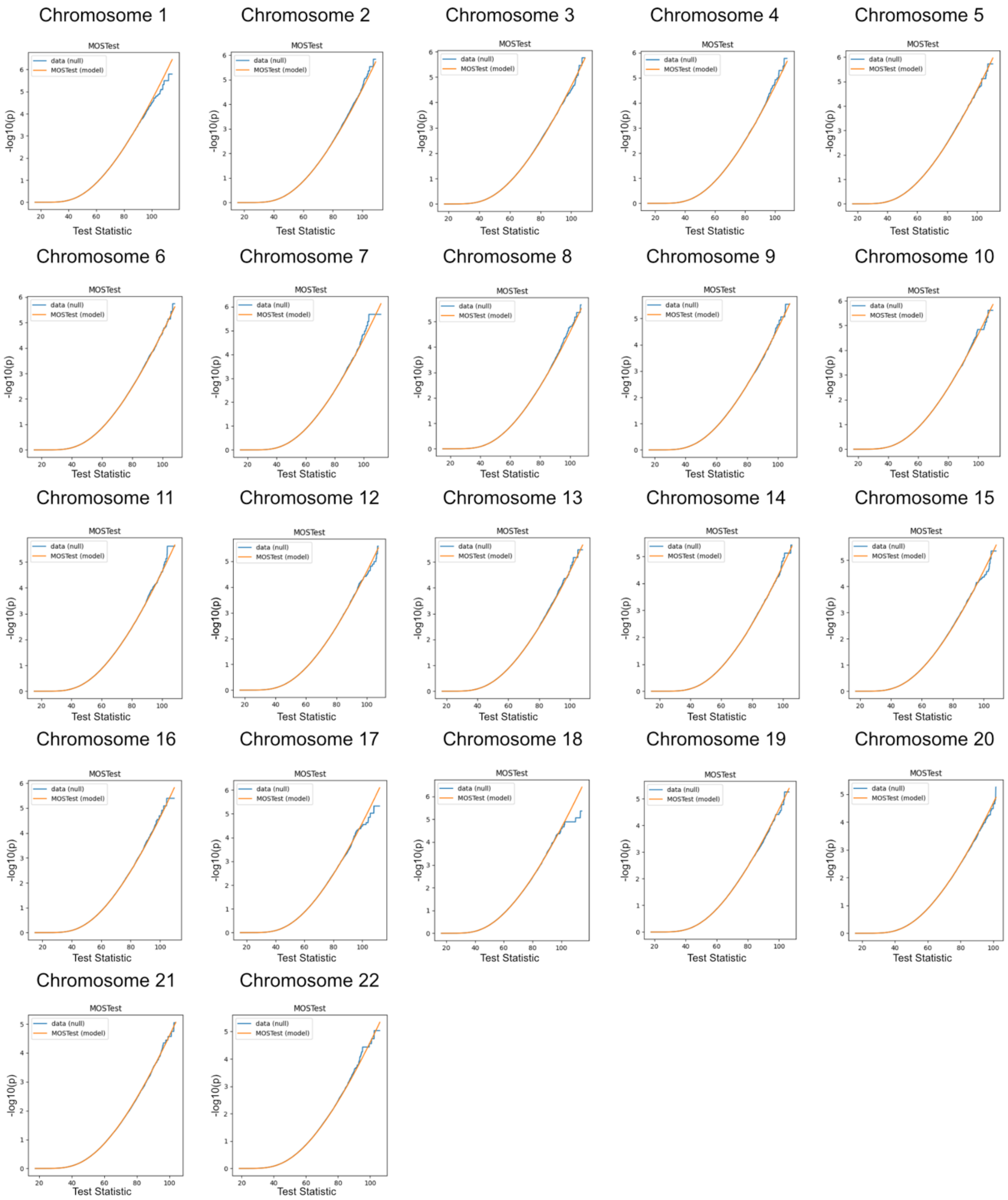

Fig. S28. Chromosome-wise Comparison of Empirical and Analytical MOSTest Results Under the Null Hypothesis for right Cerebellum White Matter. See description of Fig. S18.

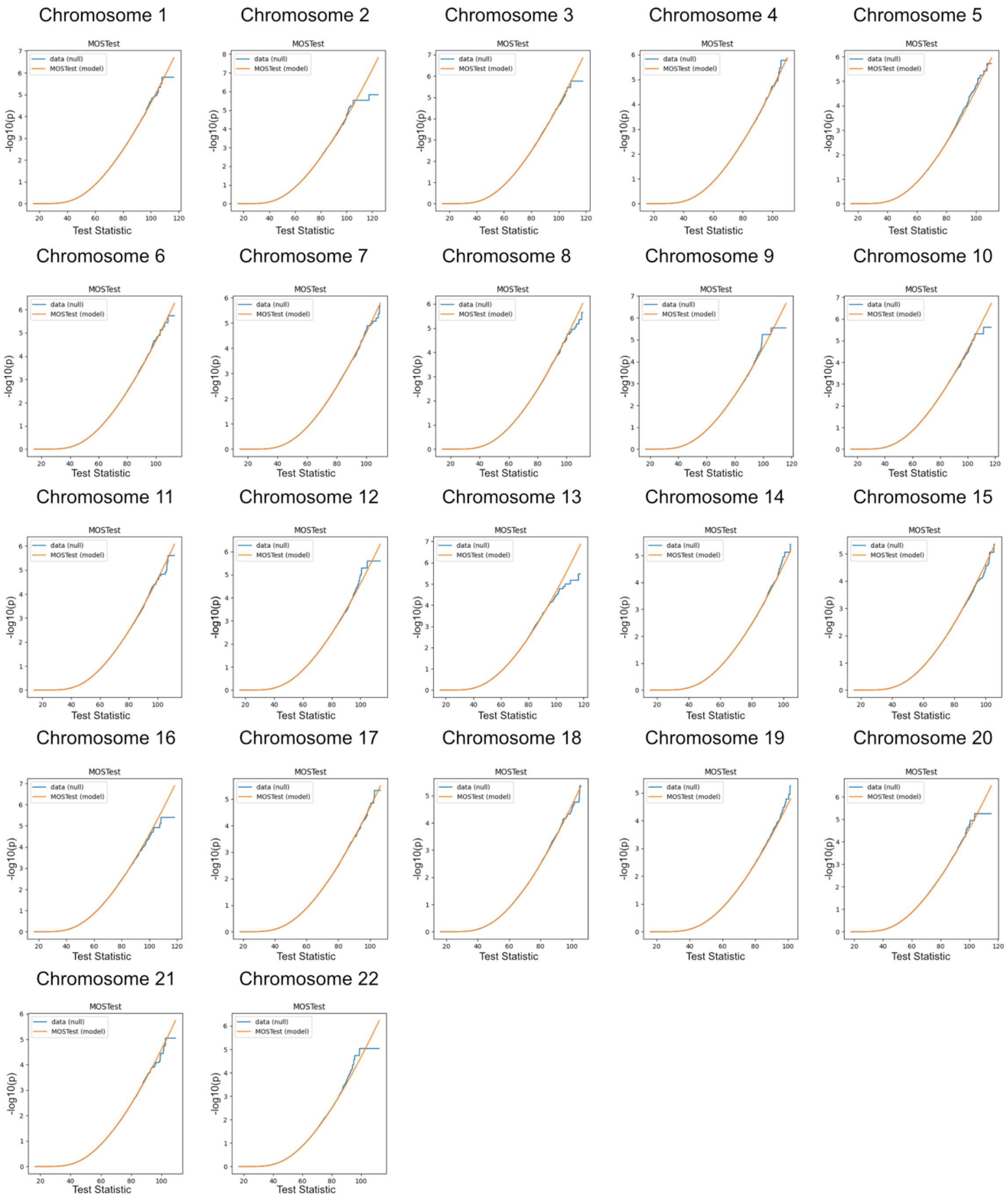

Fig. S29. Chromosome-wise Comparison of Empirical and Analytical MOSTest Results Under the Null Hypothesis for left Hippocampus. See description of Fig. S18.

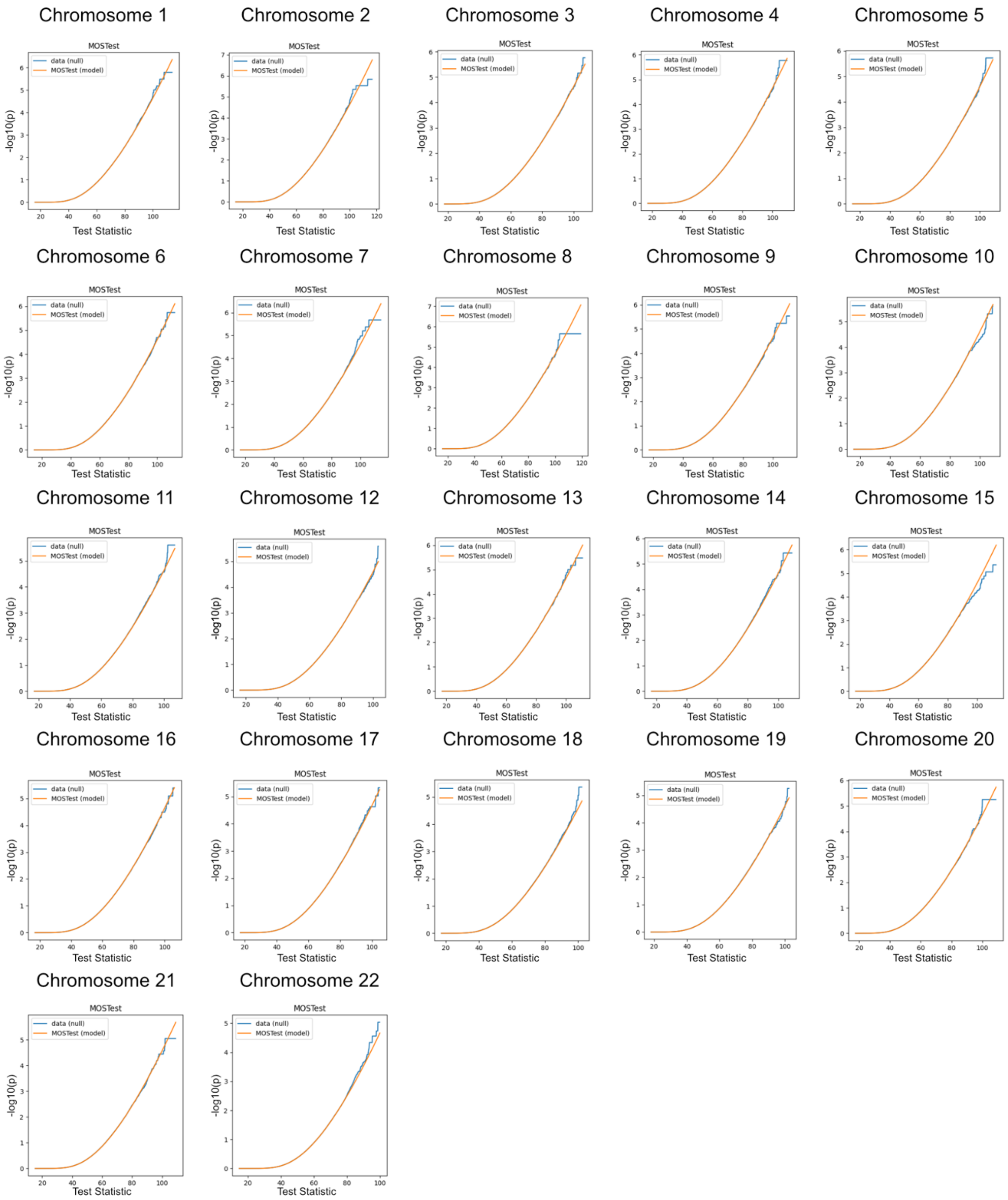

Fig. S30. Chromosome-wise Comparison of Empirical and Analytical MOSTest Results Under the Null Hypothesis for right Hippocampus. See description of Fig. S18.

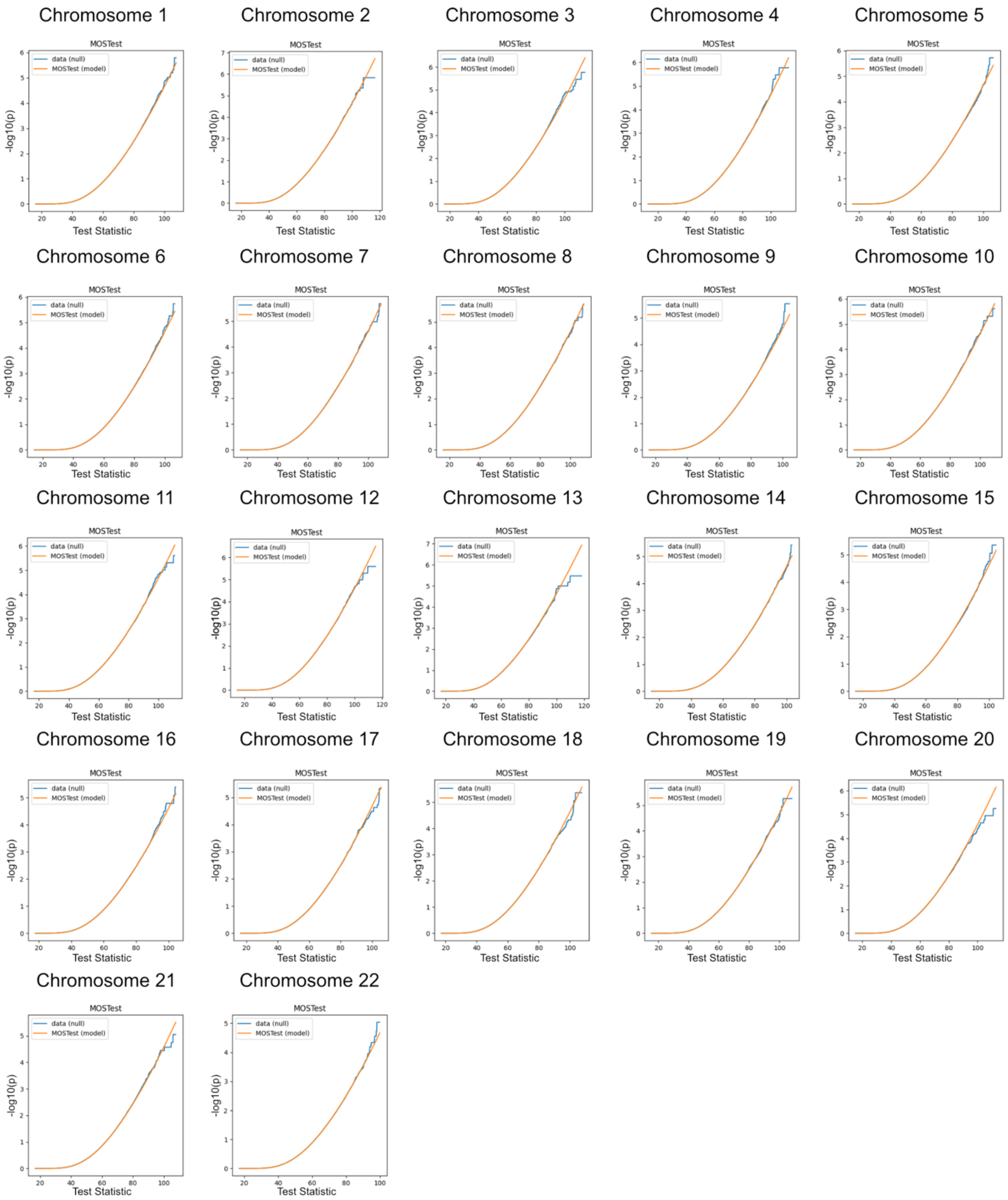

Fig. S31. Chromosome-wise Comparison of Empirical and Analytical MOSTest Results Under the Null Hypothesis for left Pallidum. See description of Fig. S18.

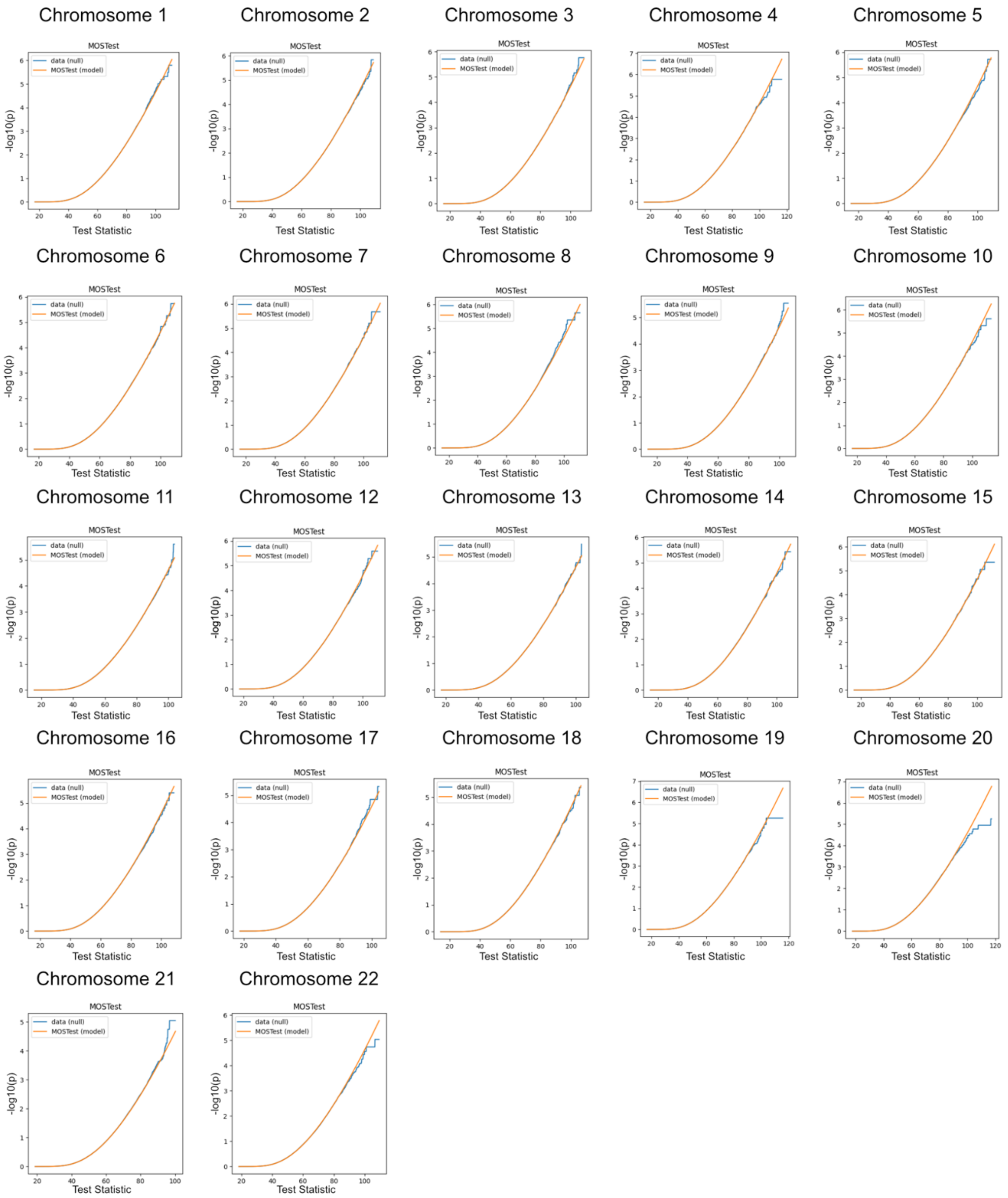

Fig. S32. Chromosome-wise Comparison of Empirical and Analytical MOSTest Results Under the Null Hypothesis for right Pallidum. See description of Fig. S18.

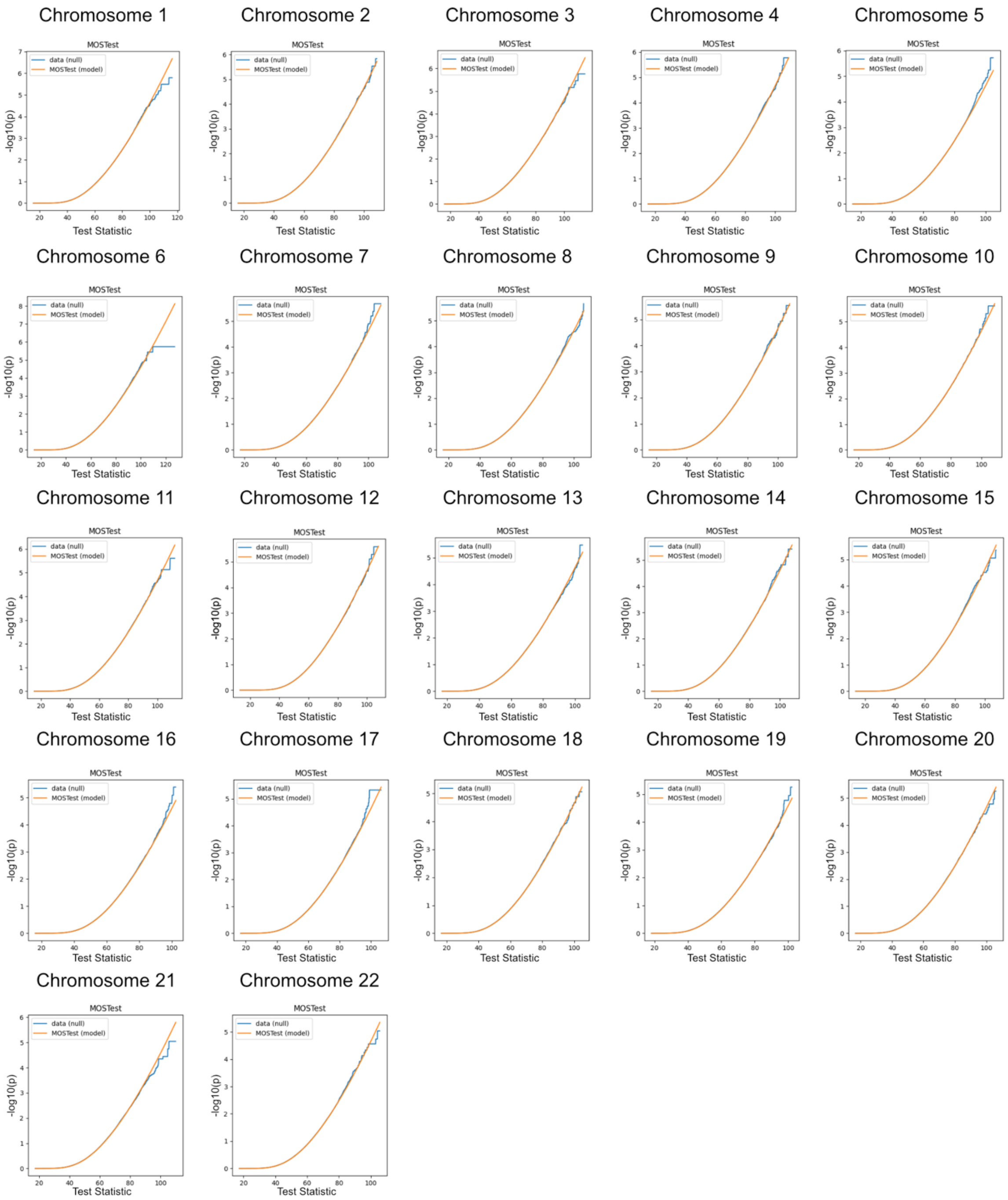

Fig. S33. Chromosome-wise Comparison of Empirical and Analytical MOSTest Results Under the Null Hypothesis for left Putamen. See description of Fig. S18.

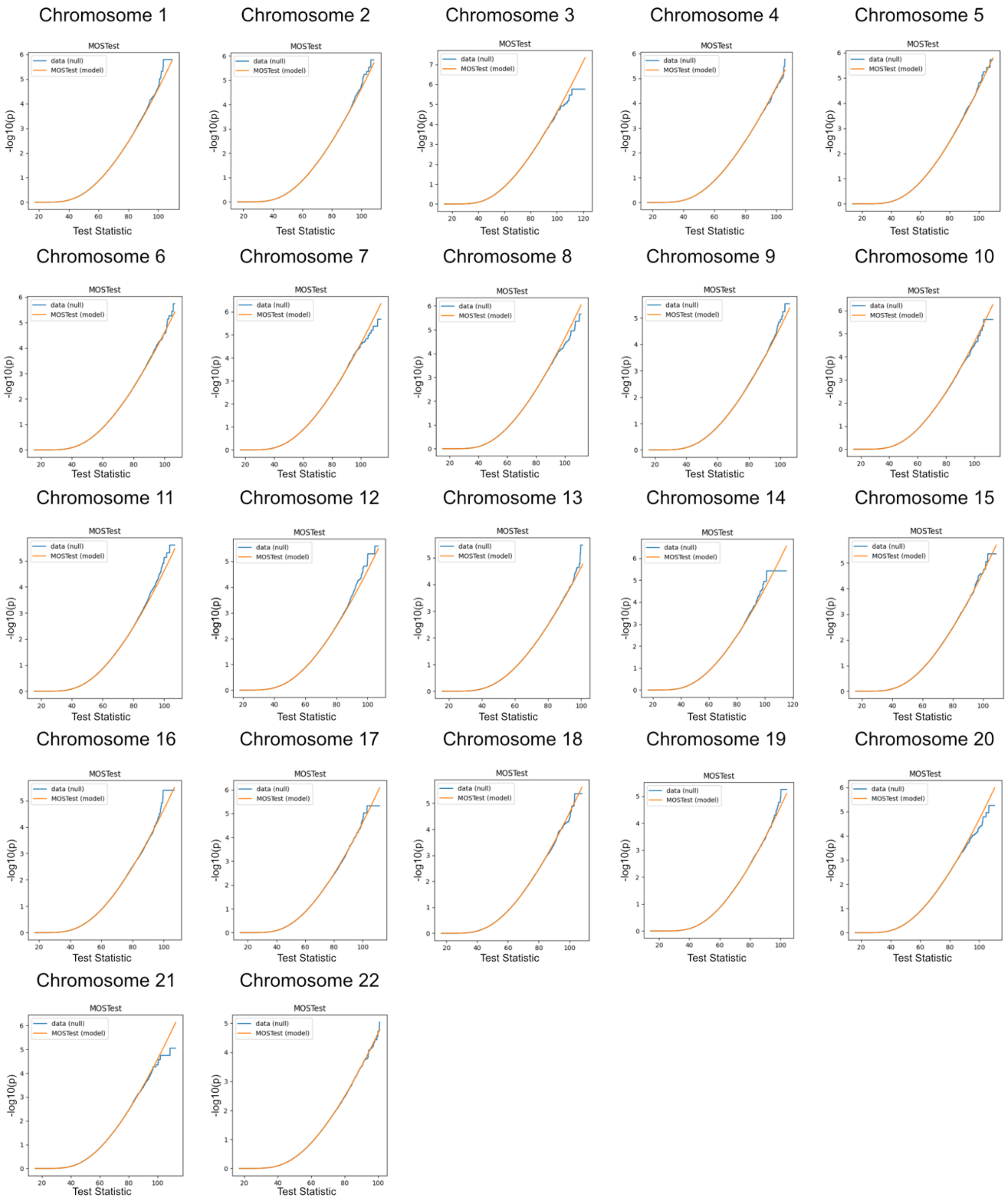

Fig. S34. Chromosome-wise Comparison of Empirical and Analytical MOSTest Results Under the Null Hypothesis for right Putamen. See description of Fig. S18.

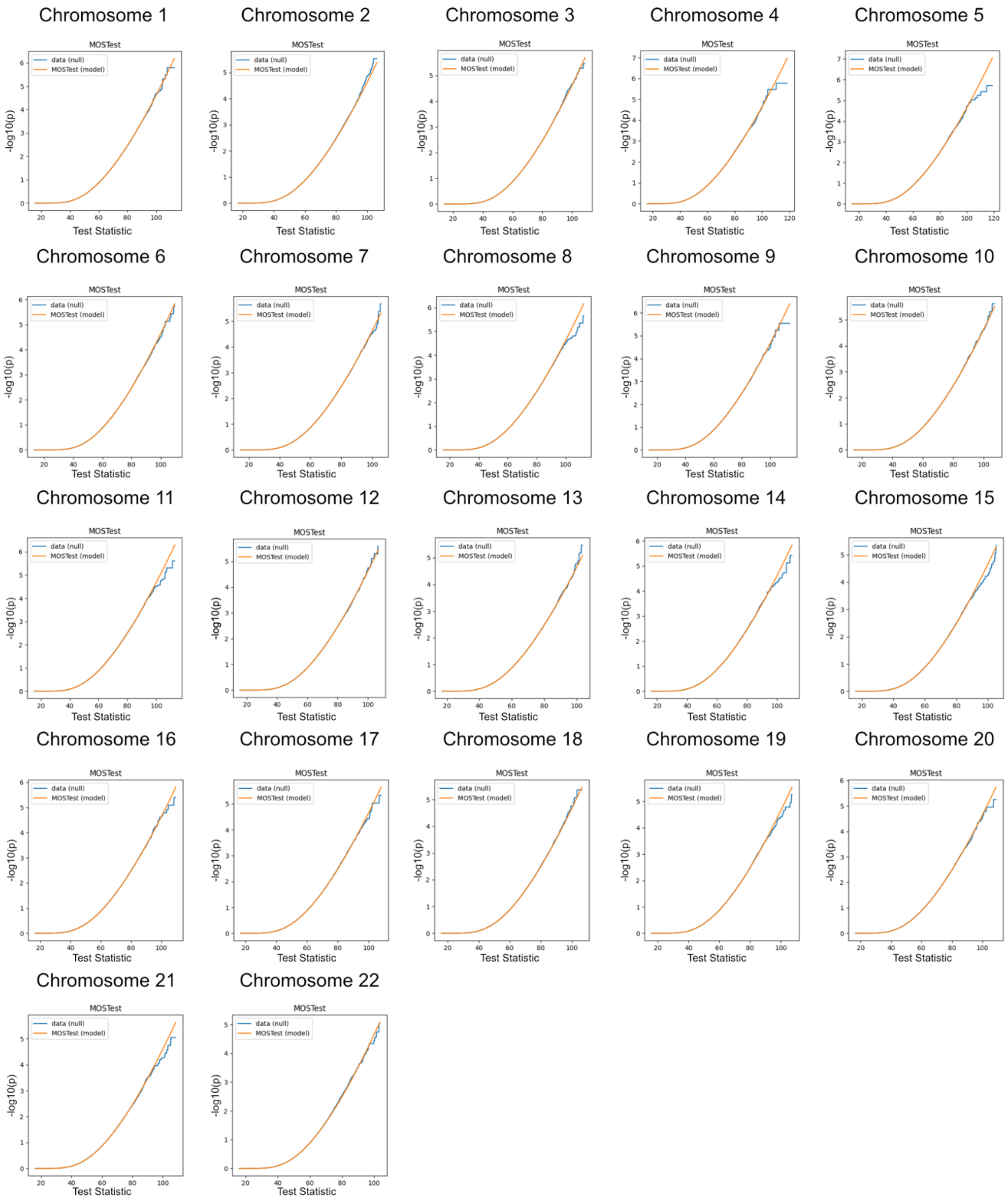

Fig. S35. Chromosome-wise Comparison of Empirical and Analytical MOSTest Results Under the Null Hypothesis for left Thalamus Proper. See description of Fig. S18.

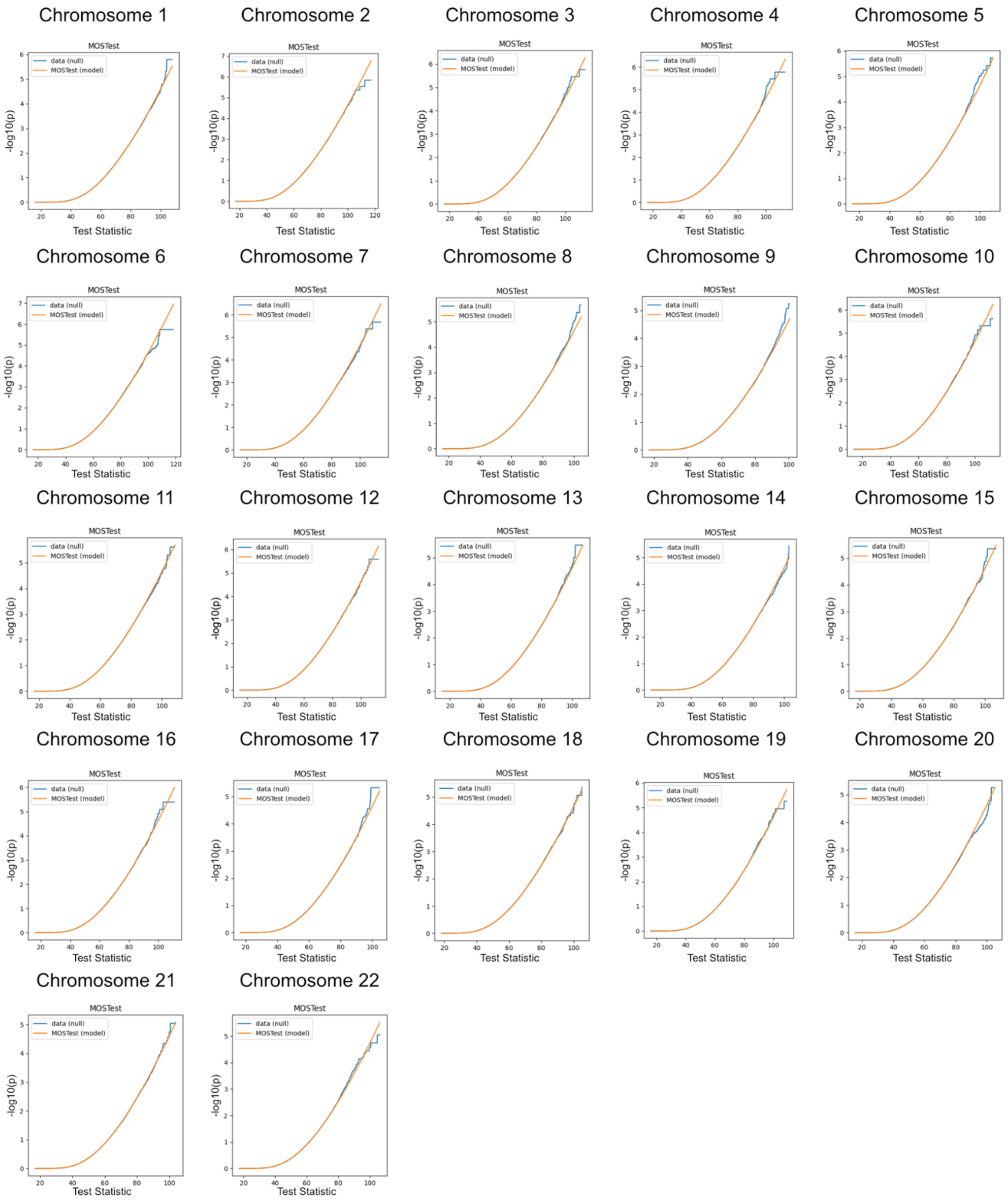

Fig. S36. Chromosome-wise Comparison of Empirical and Analytical MOSTest Results Under the Null Hypothesis for right Thalamus Proper. See description of Fig. S18.

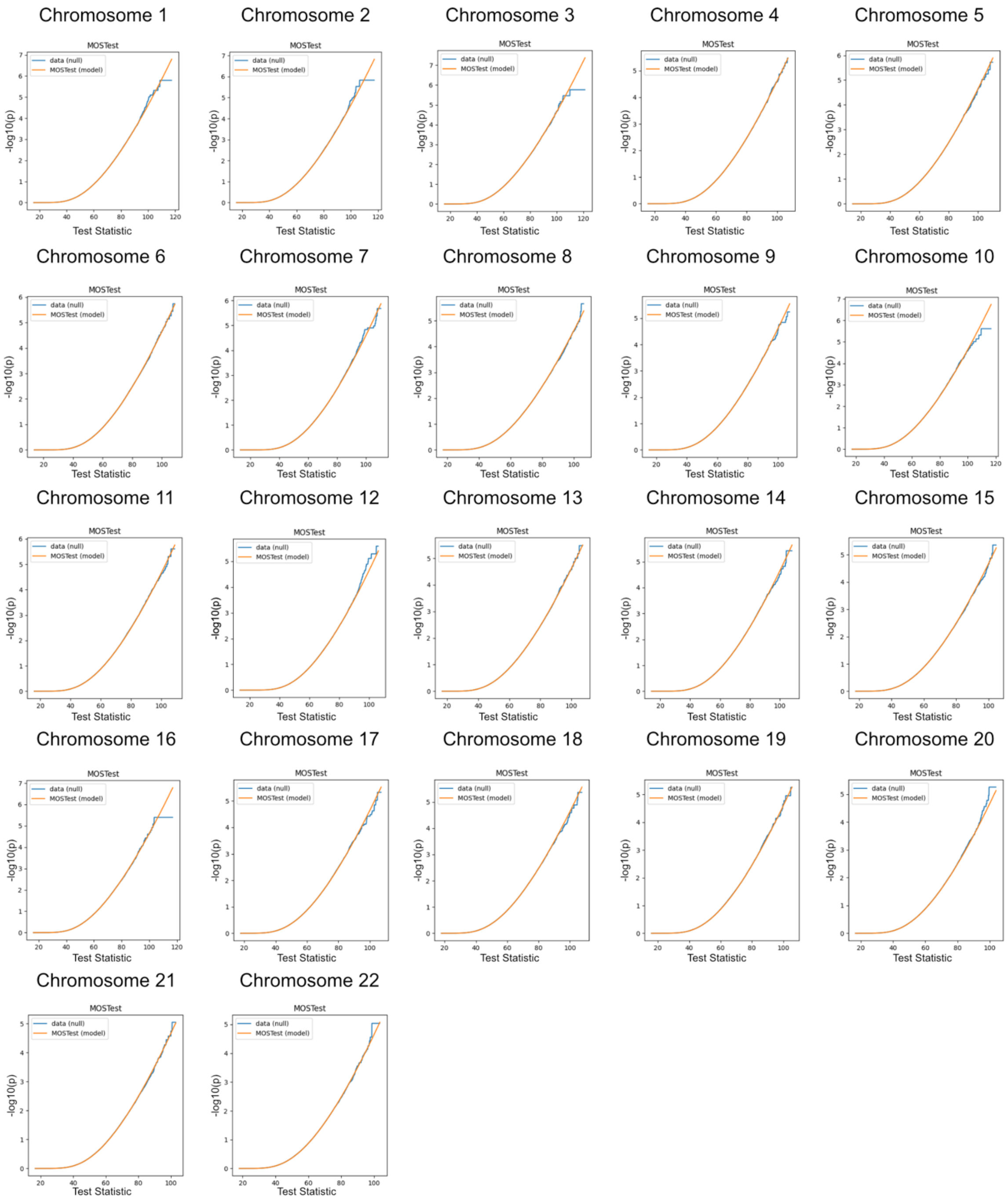

Fig. S37. Chromosome-wise Comparison of Empirical and Analytical MOSTest Results Under the Null Hypothesis for left Ventral DC. See description of Fig. S18.

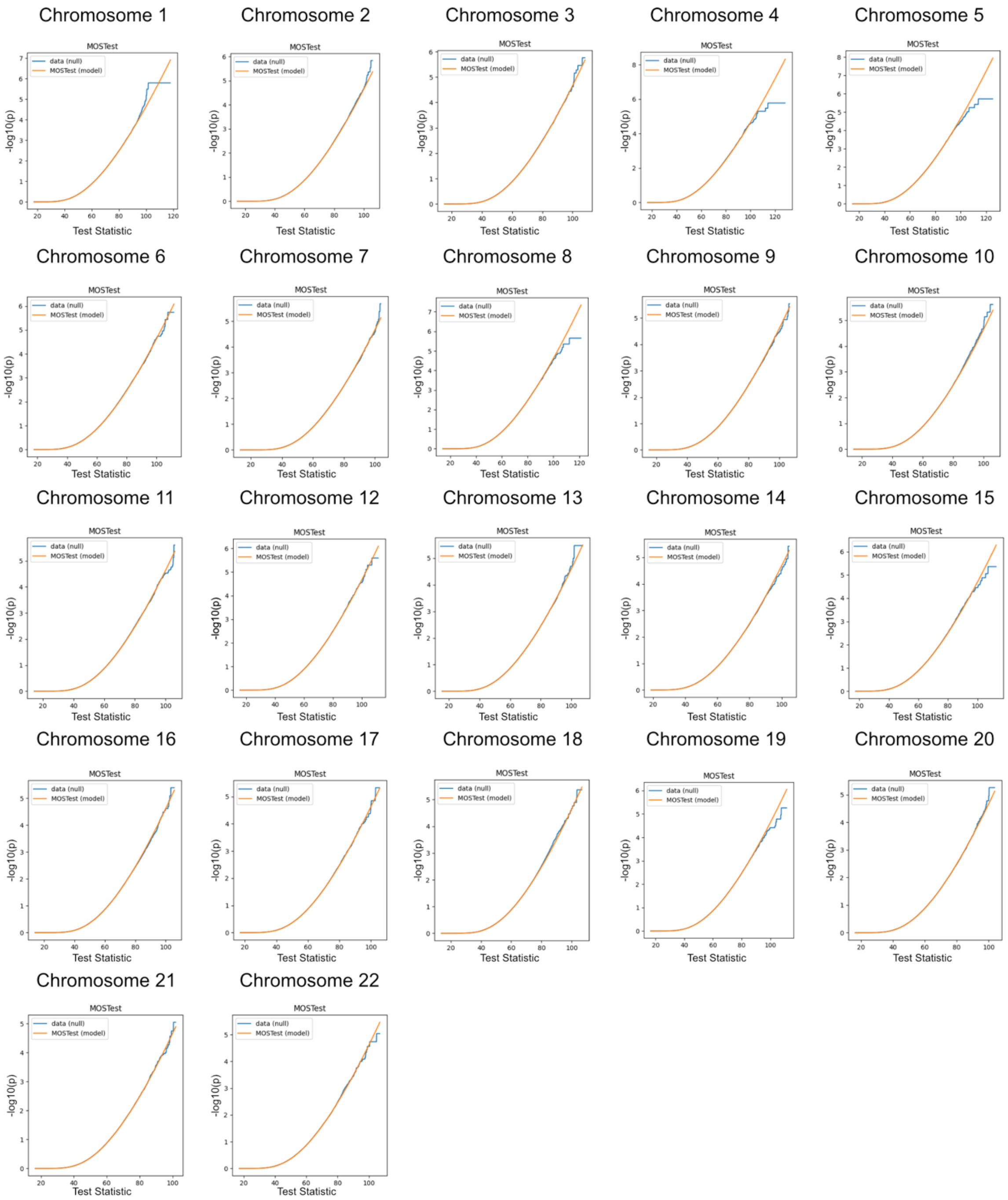

Fig. S38. Chromosome-wise Comparison of Empirical and Analytical MOSTest Results Under the Null Hypothesis for right Ventral DC. See description of Fig. S18.

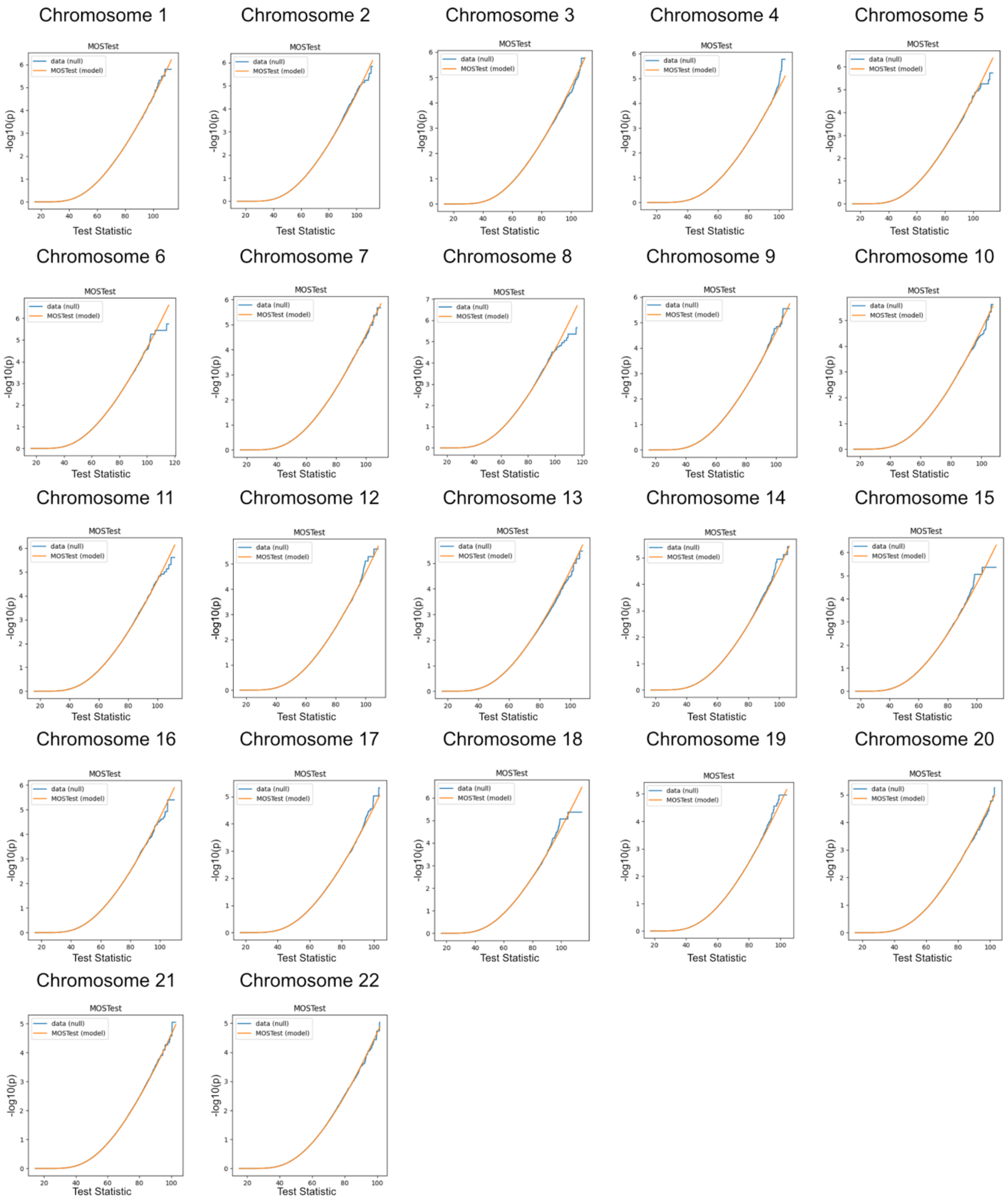

Fig. S39. Chromosome-wise Comparison of Empirical and Analytical MOSTest Results Under the Null Hypothesis for 4th Ventricle. See description of Fig. S18.

Supplementary Tables

An external Excel file contains all supplementary tables as follows:

Table S1. *Association of Independent Significant SNPs with GWAS Catalog Traits.* The individual columns show the following: *SNP*, 80 independent significant SNPs of the present study that were indicated by the analysis (MOSTest on LBS) of at least one brain structure; *LD_SNP1* and *LD_SNP2*, SNPs selected as independent significant by FUMA in at least one brain structure but belonging to the same locus ($r2 \geq 0.6$) as the variant in the SNP column; *GWAS catalog category*, whether GWAS catalog brain shape traits ("brain") defined as in Table S2, only other GWAS catalog traits ("onlyOtherTraits") or no GWAS catalog traits at all have been significantly associated with SNPs in the first three columns or proxies of them ($r2 \geq 0.6$). The subsequent columns relate the information in the fourth column to the specific brain structure(s) that were analyzed in the present study. *NA* indicates that there was no significant association after Bonferroni correction.

Table S2. *Reported Brain Shape Related Traits from GWAS Catalog*. Table lists all traits for brain volume measurements, cortical surface area measurements and cortical thickness as extracted from GWAS catalog. The last column lists additional brain shape traits from GWAS catalog, which are significantly associated with SNPs (or their proxies) that have also been found to be significant in the present study. All these traits were used to categorize SNPs into the "brain" category in Table S1.

Table S3. *Functional Annotation of the 80 Independent Significant SNPs.* Results from FUMA with non-effect allele (NEA), effect allele (EA), effect allele frequency (EAF) based on the reference panel (see Methods), distance to nearest gene (dist), and functional annotation (func) according to ANNOVAR.

Table S4. *Exonic Nonsynonymous SNPs in LD with the 80 Independent SNPs - extension of Table 1.* Results from FUMA with non-effect allele (NEA), effect allele (EA), effect allele frequency (EAF), minimum p-value in all GWASs in the present study (min_gwasP), linkage disequilibrium (r2) to the independent SNP (IndSigSNP) in the respective brain structure GWAS (brainstructure), and exon number (exon) of the gene's transcript as chosen by ANNOVAR.

Table S5. *EQTL Associations of rs1687225 that is significantly associated with Brain Stem LBS.* Results from FUMA analysis of brain-related databases (db) with p-values (p) and statistics from the eQTL association.

Table S6. *ExNS eQTLs of Protein Coding Genes in Genomic Locus 12 of Brain Stem LBS GWAS with rs568589031 as Lead SNP.* Results from FUMA as in Table S5, eQTL gene (eqtlGene) and the positional gene (posGene) as obtained by ANNOVAR.

Table S7. *P-values of the 80 Independent Significant SNPs Before and After Outlier Removal.* Table contains SNPs (rsID) with effect allele (EA), non-effect allele (NEA), and their p-values in all brain structure GWASs with the number of significant structures under different significance thresholds. P-values are colored green ($p < 2.27E-9$), blue ($2.27E-9 < p < 0.05/(80*22)$), light pink ($0.05 > p > 0.05/(80*22)$ and p of contralateral structure $< 2.27E-9$), dark pink ($p > 0.05$ and contralateral $p < 2.27E-9$) or grey ($p > 0.05$). Columns AE-AR correspond to reanalyzed results

after outlier removal. Red framed cells contain p-values of associations which have been genome-wide significant at the Bonferroni corrected threshold for 22 brain structures (2.27E-9) in the main analysis but remained above this threshold after outlier removal.

Table S8. *Coordinates of Principal Components 1-3 of -log10-scaled P-values of 80 Independent Significant SNPs.*

Table S9. *Results from GWAS Catalog for rs6658111 and rs12146713 Using LDTrait.*

Table S10. *Prioritized Genes Across all Brain Structures.* Binary coding if the gene was prioritized for the respective structures (1: True, 0: False).

Table S11. *Pearson Correlations Between Polygenic Risk Scores – Correlation coefficients.* Table contains polygenic risk scores for Alzheimer's disease (AD), bipolar disorder (BD), ischemic stroke (ISS), multiple sclerosis (MS), Parkinson's disease (PD), schizophrenia (SCZ), and alcohol use disorder (ALC).

Table S12. *Pearson Correlations Between Polygenic Risk Scores - Unadjusted P-values.* See description for Table S11.

Table S13. *Pearson Correlations Between Polygenic Risk Scores – Test Statistics.* See description for Table S11.

Table S14. *Results from Canonical Correlation Analysis.* Table contains canonical correlations (*_CCA) between each polygenic risk score (abbreviations see description of Table S11) and each brain structure and the corresponding unadjusted p-value of the correlation (*_pval).

Table S15. *Mean Loadings of CCA Results.* The table shows the mean of all loadings for each canonical correlation between polygenic risk score and brain structure eigenvalue spectrum. In parentheses, the number of negative loadings is stated. NA indicates zero negative loadings.

Table S16. *Extended Heritability Results of Single Brain Structures.* Table extends Table 2 with data on heritability variance (h^2_var) and results from the heritability Wald test (waldStats, waldP, waldP_log10).

Table S17. *Extended Heritability Results of Combined Brain Structures.* Table extends Table 2 with results from the heritability Wald test of each combined structure (waldStats, waldP, waldP_log10) and from the Wald test on difference in heritability between our results and those of (*6*) including absolute difference of heritability estimates (her_diff) and p-values of the test (waldP_diff).

Table S18. *Results of the 80 Independent Significant SNPs in Replication Data Set.* Results of all Bonferroni significant associations of the 80 independent significant SNPs (148 occurrences in total) are listed. Table shows the results with base pair (BP) and chromosome (CHR) position, effect allele (A1), non-effect allele (A2), p-value in discovery data set (PVAL_disc), in replication set (PVAL_rep), and in replication set with FDR correction for all associations in the respective brain structure (PVAL_rep_fdr). SNP is marked with a "*" for nominal significance

(PVAL_rep < 0.05) or with "**" for FDR-corrected significance (PVAL_rep_fdr < 0.05).

Table S19. *Results of Random Split Replication Analysis.* Table shows for each split and each brain structure all significant independent SNPs ($p < 2.27E-9$, $r2 < 0.6$ within 250kb to all other SNPs in the table) from the GWAS of the 80% dataset in the respective split including their chromosome (CHR) and base pair position (BP), effect allele (A1) and non-effect allele (A2). P-values of the GWAS in the 80% sample (PVAL_disc) as well as in the 20% replication sample (PVAL_rep) are shown. Replication p-values have been FDR-corrected for the number of significant independent SNPs in each brain structure and split (PVAL_rep_fdr).

Table S20. *Replication of 37 Independent Significant SNPs of Brain Stem GWAS with Height as Additional Covariate.* P-values of GWAS with height as additional covariate (PVAL_rep) are listed. SNPs are marked additionally with "!" for genome-wide significance (2.27E-9 < PVAL_rep < 5E-8) and with "!!" for Bonferroni-corrected genome-wide significance (PVAL_rep < 2.27E-9). Further column description as in Table S18.

Table S21. *Comparison of Amygdala Variants.* Table shows 12 loci from (*98*) with effect allele (A1) and non-effect allele (A2). Next, p-values and FDR-adjusted p-values are listed for left and right amygdala normalized and regressed as in the main study (*_left_vol_surf, *_right_vol_surf) and for left and right amygdala not normalized and without surface as covariate (*_left_no_norm, *_right_no_norm).

Table S22. *Results of the 80 Independent Significant SNPs in GWAS with Volume as Additional Covariate.* P-values of GWAS with volume as additional covariate (PVAL_rep) are listed. SNPs are marked additionally with "***" for suggestive significance (Bonferroni correction for 80 SNPs and 22 brain structures: 5E-8 < PVAL_rep < 0.05/(80*22)), with "!" for genome-wide significance (2.27E-9 < PVAL_rep < 5E-8) and with "!!" for Bonferroni-corrected genome-wide significance (PVAL_rep < 2.27E-9). The genomic loci were defined in the FUMA analysis of the discovery analysis. Further column description as in Table S18.

REFERENCES AND NOTES

1. P. R. Jansen, M. Nagel, K. Watanabe, Y. Wei, J. E. Savage, C. A. De Leeuw, M. P. Van Den Heuvel, S. Van Der Sluis, D. Posthuma, Genome-wide meta-analysis of brain volume identifies genomic loci and genes shared with intelligence. *Nat. Commun.* **11**, 5606 (2020).

2. D. van Der Meer, O. Frei, T. Kaufmann, A. A. Shadrin, A. Devor, O. B. Smeland, W. K. Thompson, C. C. Fan, D. Holland, L. T. Westlye, O. A. Andreassen, A. M. Dale, Understanding the genetic determinants of the brain with MOSTest. *Nat. Commun.* **11**, 3512 (2020).

3. D. P. Hibar, J. L. Stein, M. E. Renteria, A. Arias-Vasquez, S. Desrivières, N. Jahanshad, R. Toro, K. Wittfeld, L. Abramovic, M. Andersson, B. S. Aribisala, N. J. Armstrong, M. Bernard, M. M. Bohlken, M. P. Boks, J. Bralten, A. A. Brown, M. M. Chakravarty, Q. Chen, C. R. K. Ching, G. Cuellar-Partida, A. den Braber, S. Giddaluru, A. L. Goldman, O. Grimm, T. Guadalupe, J. Hass, G. Woldehawariat, A. J. Holmes, M. Hoogman, D. Janowitz, T. Jia, S. Kim, M. Klein, B. Kraemer, P. H. Lee, L. M. Olde Loohuis, M. Luciano, C. Macare, K. A. Mather, M. Mattheisen, Y. Milaneschi, K. Nho, M. Papmeyer, A. Ramasamy, S. L. Risacher, R. Roiz-Santiañez, E. J. Rose, A. Salami, P. G. Sämann, L. Schmaal, A. J. Schork, J. Shin, L. T. Strike, A. Teumer, M. M. J. van Donkelaar, K. R. van Eijk, R. K. Walters, L. T. Westlye, C. D. Whelan, A. M. Winkler, M. P. Zwiers, S. Alhusaini, L. Athanasiu, S. Ehrlich, M. M. H. Hakobjan, C. B. Hartberg, U. K. Haukvik, A. J. G. A. M. Heister, D. Hoehn, D. Kasperaviciute, D. C. M. Liewald, L. M. Lopez, R. R. R. Makkinje, M. Matarin, M. A. M. Naber, D. R. McKay, M. Needham, A. C. Nugent, B. Pütz, N. A. Royle, L. Shen, E. Sprooten, D. Trabzuni, S. S. L. van der Marel, K. J. E. van Hulzen, E. Walton, C. Wolf, L. Almasy, D. Ames, S. Arepalli, A. A. Assareh, M. E. Bastin, H. Brodaty, K. B. Bulayeva, M. A. Carless, S. Cichon, A. Corvin, J. E. Curran, M. Czisch, G. I. de Zubicaray, A. Dillman, R. Duggirala, T. D. Dyer, S. Erk, I. O. Fedko, L. Ferrucci, T. M. Foroud, P. T. Fox, M. Fukunaga, J. R. Gibbs, H. H. H. Göring, R. C. Green, S. Guelfi, N. K. Hansell, C. A. Hartman, K. Hegenscheid, A. Heinz, D. G. Hernandez, D. J. Heslenfeld, P. J. Hoekstra, F. Holsboer, G. Homuth, J.-J. Hottenga, M. Ikeda, C. R. Jack, M. Jenkinson, R. Johnson, R. Kanai, M. Keil, J. W. Kent, P. Kochunov, J. B. Kwok, S. M. Lawrie, X. Liu, D. L. Longo, K. L. McMahon, E. Meisenzahl, I. Melle, S. Mohnke, G. W. Montgomery, J. C. Mostert, T. W. Mühleisen, M. A. Nalls, T. E. Nichols, L. G. Nilsson, M. M. Nöthen, K. Ohi, R. L. Olvera, R. Perez-Iglesias, G. B. Pike, S.

G. Potkin, I. Reinvang, S. Reppermund, M. Rietschel, N. Romanczuk-Seiferth, G. D. Rosen, D. Rujescu, K. Schnell, P. R. Schofield, C. Smith, V. M. Steen, J. E. Sussmann, A. Thalamuthu, A. W. Toga, B. J. Traynor, J. Troncoso, J. A. Turner, M. C. Valdés Hernández, D. van't Ent, M. van der Brug, N. J. A. van der Wee, M.-J. van Tol, D. J. Veltman, T. H. Wassink, E. Westman, R. H. Zielke, A. B. Zonderman, D. G. Ashbrook, R. Hager, L. Lu, F. J. McMahon, D. W. Morris, R. W. Williams, H. G. Brunner, R. L. Buckner, J. K. Buitelaar, W. Cahn, V. D. Calhoun, G. L. Cavalleri, B. Crespo-Facorro, A. M. Dale, G. E. Davies, N. Delanty, C. Depondt, S. Djurovic, W. C. Drevets, T. Espeseth, R. L. Gollub, B.-C. Ho, W. Hoffmann, N. Hosten, R. S. Kahn, S. Le Hellard, A. Meyer-Lindenberg, B. Müller-Myhsok, M. Nauck, L. Nyberg, M. Pandolfo, B. W. J. H. Penninx, J. L. Roffman, S. M. Sisodiya, J. W. Smoller, H. van Bokhoven, N. E. M. van Haren, H. Völzke, H. Walter, M. W. Weiner, W. Wen, T. White, I. Agartz, O. A. Andreassen, J. Blangero, D. I. Boomsma, R. M. Brouwer, D. M. Cannon, M. R. Cookson, E. J. C. de Geus, I. J. Deary, G. Donohoe, G. Fernández, S. E. Fisher, C. Francks, D. C. Glahn, H. J. Grabe, O. Gruber, J. Hardy, R. Hashimoto, H. E. Hulshoff Pol, E. G. Jönsson, I. Kloszewska, S. Lovestone, V. S. Mattay, P. Mecocci, C. McDonald, A. M. McIntosh, R. A. Ophoff, T. Paus, Z. Pausova, M. Ryten, P. S. Sachdev, A. J. Saykin, A. Simmons, A. Singleton, H. Soininen, J. M. Wardlaw, M. E. Weale, D. R. Weinberger, H. H. H. Adams, L. J. Launer, S. Seiler, R. Schmidt, G. Chauhan, C. L. Satizabal, J. T. Becker, L. Yanek, S. J. van der Lee, M. Ebling, B. Fischl, W. T. Longstreth, D. Greve, H. Schmidt, P. Nyquist, L. N. Vinke, C. M. van Duijn, L. Xue, B. Mazoyer, J. C. Bis, V. Gudnason, S. Seshadri, M. A. Ikram; Alzheimer's Disease Neuroimaging Initiative; CHARGE Consortium; EPIGEN; IMAGEN; SYS; N. G. Martin, M. J. Wright, G. Schumann, B. Franke, P. M. Thompson, S. E. Medland, Common genetic variants influence human subcortical brain structures. *Nature* **520**, 224–229 (2015).

4. C. L. Satizabal, H. H. H. Adams, D. P. Hibar, C. C. White, M. J. Knol, J. L. Stein, M. Scholz, M. Sargurupremraj, N. Jahanshad, G. V. Roshchupkin, A. V. Smith, J. C. Bis, X. Jian, M. Luciano, E. Hofer, A. Teumer, S. J. Van Der Lee, J. Yang, L. R. Yanek, T. V. Lee, S. Li, Y. Hu, J. Y. Koh, J. D. Eicher, S. Desrivières, A. Arias-Vasquez, G. Chauhan, L. Athanasiu, M. E. Rentería, S. Kim, D. Hoehn, N. J. Armstrong, Q. Chen, A. J. Holmes, A. Den Braber, I. Kloszewska, M. Andersson, T. Espeseth, O. Grimm, L. Abramovic, S. Alhusaini, Y. Milaneschi, M. Papmeyer, T. Axelsson, S. Ehrlich, R. Roiz-Santiañez, B. Kraemer, A. K. Håberg, H. J. Jones, G. B. Pike, D. J. Stein, A. Stevens, J. Bralten, M. W. Vernooij, T. B.

Harris, I. Filippi, A. V. Witte, T. Guadalupe, K. Wittfeld, T. H. Mosley, J. T. Becker, N. T. Doan, S. P. Hagenaars, Y. Saba, G. Cuellar-Partida, N. Amin, S. Hilal, K. Nho, N. Mirza-Schreiber, K. Arfanakis, D. M. Becker, D. Ames, A. L. Goldman, P. H. Lee, D. I. Boomsma, S. Lovestone, S. Giddaluru, S. Le Hellard, M. Mattheisen, M. M. Bohlken, D. Kasperaviciute, L. Schmaal, S. M. Lawrie, I. Agartz, E. Walton, D. Tordesillas-Gutierrez, G. E. Davies, J. Shin, J. C. Ipser, L. N. Vinke, M. Hoogman, T. Jia, R. Burkhardt, M. Klein, F. Crivello, D. Janowitz, O. Carmichael, U. K. Haukvik, B. S. Aribisala, H. Schmidt, L. T. Strike, C.-Y. Cheng, S. L. Risacher, B. Pütz, D. A. Fleischman, A. A. Assareh, V. S. Mattay, R. L. Buckner, P. Mecocci, A. M. Dale, S. Cichon, M. P. Boks, M. Matarin, B. W. J. H. Penninx, V. D. Calhoun, M. M. Chakravarty, A. F. Marquand, C. Macare, S. K. Masouleh, J. Oosterlaan, P. Amouyel, K. Hegenscheid, J. I. Rotter, A. J. Schork, D. C. M. Liewald, G. I. De Zubicaray, T. Y. Wong, L. Shen, P. G. Sämann, H. Brodaty, J. L. Roffman, E. J. C. De Geus, M. Tsolaki, S. Erk, K. R. Van Eijk, G. L. Cavalleri, N. J. A. Van Der Wee, A. M. McIntosh, R. L. Gollub, K. B. Bulayeva, M. Bernard, J. S. Richards, J. J. Himali, M. Loeffler, N. Rommelse, W. Hoffmann, L. T. Westlye, M. C. Valdés Hernández, N. K. Hansell, T. G. M. Van Erp, C. Wolf, J. B. J. Kwok, B. Vellas, A. Heinz, L. M. Olde Loohuis, N. Delanty, B.-C. Ho, C. R. K. Ching, E. Shumskaya, B. Singh, A. Hofman, D. Van Der Meer, G. Homuth, B. M. Psaty, M. E. Bastin, G. W. Montgomery, T. M. Foroud, S. Reppermund, J.-J. Hottenga, A. Simmons, A. Meyer-Lindenberg, W. Cahn, C. D. Whelan, M. M. J. Van Donkelaar, Q. Yang, N. Hosten, R. C. Green, A. Thalamuthu, S. Mohnke, H. E. Hulshoff Pol, H. Lin, C. R. Jack, P. R. Schofield, T. W. Mühleisen, P. Maillard, S. G. Potkin, W. Wen, E. Fletcher, A. W. Toga, O. Gruber, M. Huentelman, G. Davey Smith, L. J. Launer, L. Nyberg, E. G. Jönsson, B. Crespo-Facorro, N. Koen, D. N. Greve, A. G. Uitterlinden, D. R. Weinberger, V. M. Steen, I. O. Fedko, N. A. Groenewold, W. J. Niessen, R. Toro, C. Tzourio, W. T. Longstreth, M. K. Ikram, J. W. Smoller, M.-J. Van Tol, J. E. Sussmann, T. Paus, H. Lemaître, M. L. Schroeter, B. Mazoyer, O. A. Andreassen, F. Holsboer, C. Depondt, D. J. Veltman, J. A. Turner, Z. Pausova, G. Schumann, D. Van Rooij, S. Djurovic, I. J. Deary, K. L. McMahon, B. Müller-Myhsok, R. M. Brouwer, H. Soininen, M. Pandolfo, T. H. Wassink, J. W. Cheung, T. Wolfers, J.-L. Martinot, M. P. Zwiers, M. Nauck, I. Melle, N. G. Martin, R. Kanai, E. Westman, R. S. Kahn, S. M. Sisodiya, T. White, A. Saremi, H. Van Bokhoven, H. G. Brunner, H. Völzke, M. J. Wright, D. Van'T Ent, M. M. Nöthen, R. A. Ophoff, J. K. Buitelaar, G. Fernández, P. S. Sachdev, M. Rietschel, N. E. M. Van Haren, S. E. Fisher, A. S. Beiser, C. Francks, A. J. Saykin, K. A. Mather, N.

Romanczuk-Seiferth, C. A. Hartman, A. L. DeStefano, D. J. Heslenfeld, M. W. Weiner, H. Walter, P. J. Hoekstra, P. A. Nyquist, B. Franke, D. A. Bennett, H. J. Grabe, A. D. Johnson, C. Chen, C. M. Van Duijn, O. L. Lopez, M. Fornage, J. M. Wardlaw, R. Schmidt, C. DeCarli, P. L. De Jager, A. Villringer, S. Debette, V. Gudnason, S. E. Medland, J. M. Shulman, P. M. Thompson, S. Seshadri, M. A. Ikram, Genetic architecture of subcortical brain structures in 38,851 individuals. *Nat. Genet.* **51**, 1624–1636 (2019).

5. T. Elvsåshagen, S. Bahrami, D. Van Der Meer, I. Agartz, D. Alnæs, D. M. Barch, R. Baur-Streubel, A. Bertolino, M. K. Beyer, G. Blasi, S. Borgwardt, B. Boye, J. Buitelaar, E. Bøen, E. G. Celius, S. Cervenka, A. Conzelmann, D. Coynel, P. Di Carlo, S. Djurovic, S. Eisenacher, T. Espeseth, H. Fatouros-Bergman, L. Flyckt, B. Franke, O. Frei, B. Gelao, H. F. Harbo, C. A. Hartman, A. Håberg, D. Heslenfeld, P. J. Hoekstra, E. A. Høgestøl, R. Jonassen, E. G. Jönsson, Karolinska Schizophrenia Project (KaSP) consortium, L. Farde, L. Flyckt, G. Engberg, H. Fatouros-Bergman, S. Cervenka, L. Schwieler, F. Piehl, I. Agartz, K. Collste, P. Victorsson, A. Malmqvist, M. Hedberg, F. Orhan, C. M. Sellgren, P. Kirsch, I. Kłoszewska, T. V. Lagerberg, N. I. Landrø, S. Le Hellard, K.-P. Lesch, L. A. Maglanoc, U. F. Malt, P. Mecocci, I. Melle, A. Meyer-Lindenberg, T. Moberget, J. E. Nordvik, L. Nyberg, K. S. O. Connell, J. Oosterlaan, M. Papalino, A. Papassotiropoulos, P. Pauli, G. Pergola, K. Persson, D. De Quervain, A. Reif, J. Rokicki, D. Van Rooij, A. A. Shadrin, A. Schmidt, E. Schwarz, G. Selbæk, H. Soininen, P. Sowa, V. M. Steen, M. Tsolaki, B. Vellas, L. Wang, E. Westman, G. C. Ziegler, M. Zink, O. A. Andreassen, L. T. Westlye, T. Kaufmann, The genetic architecture of human brainstem structures and their involvement in common brain disorders. *Nat. Commun.* **11**, 4016 (2020).

6. T. Ge, M. Reuter, A. M. Winkler, A. J. Holmes, P. H. Lee, L. S. Tirrell, J. L. Roffman, R. L. Buckner, J. W. Smoller, M. R. Sabuncu, Multidimensional heritability analysis of neuroanatomical shape. *Nat. Commun.* **7**, 13291 (2016).

7. G. V. Roshchupkin, B. A. Gutman, M. W. Vernooij, N. Jahanshad, N. G. Martin, A. Hofman, K. L. McMahon, S. J. Van Der Lee, C. M. Van Duijn, G. I. De Zubicaray, A. G. Uitterlinden, M. J. Wright, W. J. Niessen, P. M. Thompson, M. A. Ikram, H. H. H. Adams, Heritability of the shape of subcortical brain structures in the general population. *Nat. Commun.* **7**, 13738 (2016).

8. B. A. Gutman, T. G. M. Van Erp, K. Alpert, C. R. K. Ching, D. Isaev, A. Ragothaman, N. Jahanshad, A. Saremi, A. Zavaliangos-Petropulu, D. C. Glahn, L. Shen, S. Cong, D. Alnæs, O. A. Andreassen, N. T. Doan, L. T. Westlye, P. Kochunov, T. D. Satterthwaite, D. H. Wolf, A. J. Huang, C. Kessler, A. Weideman, D. Nguyen, B. A. Mueller, L. Faziola, S. G. Potkin, A. Preda, D. H. Mathalon, J. Bustillo, V. Calhoun, J. M. Ford, E. Walton, S. Ehrlich, G. Ducci, N. Banaj, F. Piras, F. Piras, G. Spalletta, E. J. Canales-Rodríguez, P. Fuentes-Claramonte, E. Pomarol-Clotet, J. Radua, R. Salvador, S. Sarró, E. W. Dickie, A. Voineskos, D. Tordesillas-Gutiérrez, B. Crespo-Facorro, E. Setién-Suero, J. M. Van Son, S. Borgwardt, F. Schönborn-Harrisberger, D. Morris, G. Donohoe, L. Holleran, D. Cannon, C. McDonald, A. Corvin, M. Gill, G. B. Filho, P. G. P. Rosa, M. H. Serpa, M. V. Zanetti, I. Lebedeva, V. Kaleda, A. Tomyshev, T. Crow, A. James, S. Cervenka, C. M. Sellgren, H. Fatouros-Bergman, I. Agartz, F. Howells, D. J. Stein, H. Temmingh, A. Uhlmann, G. I. De Zubicaray, K. L. McMahon, M. Wright, D. Cobia, J. G. Csernansky, P. M. Thompson, J. A. Turner, L. Wang, A meta-analysis of deep brain structural shape and asymmetry abnormalities in 2,833 individuals with schizophrenia compared with 3,929 healthy volunteers via the ENIGMA Consortium. *Hum. Brain Mapp.* **43**, 352–372 (2022).

9. L. T. Elliott, K. Sharp, F. Alfaro-Almagro, S. Shi, K. L. Miller, G. Douaud, J. Marchini, S. M. Smith, Genome-wide association studies of brain imaging phenotypes in UK Biobank. *Nature* **562**, 210–216 (2018).

10. C. C. Fan, R. Loughnan, C. Makowski, D. Pecheva, C.-H. Chen, D. J. Hagler Jr., W. K. Thompson, N. Parker, D. van der Meer, O. Frei, O. A. Andreassen, A. M. Dale, Multivariate genome-wide association study on tissue-sensitive diffusion metrics highlights pathways that shape the human brain. *Nat. Commun.* **13**, 2423 (2022).

11. S. Goovaerts, H. Hoskens, R. J. Eller, N. Herrick, A. M. Musolf, C. M. Justice, M. Yuan, S. Naqvi, M. K. Lee, D. Vandermeulen, H. L. Szabo-Rogers, P. A. Romitti, S. A. Boyadjiev, M. L. Marazita, J. R. Shaffer, M. D. Shriver, J. Wysocka, S. Walsh, S. M. Weinberg, P. Claes, Joint multi-ancestry and admixed GWAS reveals the complex genetics behind human cranial vault shape. *Nat. Commun.* **14**, 7436 (2023).

12. S. Naqvi, Y. Sleyp, H. Hoskens, K. Indencleef, J. P. Spence, R. Bruffaerts, A. Radwan, R. J. Eller, S. Richmond, M. D. Shriver, J. R. Shaffer, S. M. Weinberg, S. Walsh, J. Thompson, J. K. Pritchard, S. Sunaert, H. Peeters, J. Wysocka, P. Claes, Shared heritability of human face and brain shape. *Nat. Genet.* **53**, 830–839 (2021).

13. M. Reuter, F.-E. Wolter, N. Peinecke, Laplace–Beltrami spectra as 'Shape-DNA' of surfaces and solids. *Computer-Aided Design* **38**, 342–366 (2006).

14. M. Reuter, F.-E. Wolter, N. Peinecke, "Laplace-spectra as fingerprints for shape matching," in *Proceedings of the 2005 ACM Symposium on Solid and Physical Modeling* (ACM, 2005), pp. 101–106.

15. M. Reuter, F.-E. Wolter, M. Shenton, M. Niethammer, Laplace–Beltrami eigenvalues and topological features of eigenfunctions for statistical shape analysis. *Comput.-Aided Des.* **41**, 739–755 (2009).

16. E. F. F. Chladni, *Entdeckungen über die Theorie des Klanges* (bey Weidmanns Erben und Reich, 1787).

17. M. Kac, Can one hear the shape of a drum? *Am. Math. Monthly* **73**, 1–23 (1966).

18. C. Gordon, D. L. Webb, S. Wolpert, One cannot hear the shape of a drum. *Bull. Amer. Math. Soc.* **27**, 134–138 (1992).

19. S. Zelditch, Inverse spectral problem for analytic domains, II: $\mathbb{Z}_2$-symmetric domains. *Ann. Math.* **170**, 205–269 (2009).

20. H. P. McKean Jr., I. M. Singer, Curvature and the eigenvalues of the Laplacian. *J. Differential Geom.* **1**, 43–69 (1967).

21. C. Wachinger, P. Golland, W. Kremen, B. Fischl, M. Reuter, Alzheimer's Disease Neuroimaging Initiative, BrainPrint: A discriminative characterization of brain morphology. *Neuroimage* **109**, 232–248 (2015).

22. J. C. Pang, K. M. Aquino, M. Oldehinkel, P. A. Robinson, B. D. Fulcher, M. Breakspear, A. Fornito, Geometric constraints on human brain function. *Nature* **618**, 566–574 (2023).

23. D. Van Der Meer, A. A. Shadrin, K. O'Connell, F. Bettella, S. Djurovic, T. Wolfers, D. Alnæs, I. Agartz, O. B. Smeland, I. Melle, J. M. Sánchez, D. E. J. Linden, A. M. Dale, L. T. Westlye, O. A. Andreassen, O. Frei, T. Kaufmann, Boosting schizophrenia genetics by utilizing genetic overlap with brain morphology. *Biol. Psychiatry* **92**, 291–298 (2022).

24. S. Bahrami, K. Nordengen, A. A. Shadrin, O. Frei, D. Van Der Meer, A. M. Dale, L. T. Westlye, O. A. Andreassen, T. Kaufmann, Distributed genetic architecture across the hippocampal formation implies common neuropathology across brain disorders. *Nat. Commun.* **13**, 3436 (2022).

25. T. Elvsåshagen, A. Shadrin, O. Frei, D. Van Der Meer, S. Bahrami, V. J. Kumar, O. Smeland, L. T. Westlye, O. A. Andreassen, T. Kaufmann, The genetic architecture of the human thalamus and its overlap with ten common brain disorders. *Nat. Commun.* **12**, 2909 (2021).

26. H. H. Yin, B. J. Knowlton, The role of the basal ganglia in habit formation. *Nat. Rev. Neurosci.* **7**, 464–476 (2006).

27. L. T. Grinberg, U. Rueb, H. Heinsen, Brainstem: Neglected locus in neurodegenerative diseases. *Front. Neurol.* **2**, 42 (2011).

28. K. Watanabe, E. Taskesen, A. van Bochoven, D. Posthuma, Functional mapping and annotation of genetic associations with FUMA. *Nat. Commun.* **8**, 1826 (2017).

29. K. Wang, M. Li, H. Hakonarson, ANNOVAR: Functional annotation of genetic variants from high-throughput sequencing data. *Nucleic Acids Res.* **38**, e164 (2010).

30. M. Kircher, D. M. Witten, P. Jain, B. J. O'Roak, G. M. Cooper, J. Shendure, A general framework for estimating the relative pathogenicity of human genetic variants. *Nat. Genet.* **46**, 310–315 (2014).

31. M. J. Landrum, S. Chitipiralla, G. R. Brown, C. Chen, B. Gu, J. Hart, D. Hoffman, W. Jang, K. Kaur, C. Liu, V. Lyoshin, Z. Maddipatla, R. Maiti, J. Mitchell, N. O'Leary, G. R. Riley, W.

Shi, G. Zhou, V. Schneider, D. Maglott, J. B. Holmes, B. L. Kattman, ClinVar: Improvements to accessing data. *Nucleic Acids Res.* **48**, D835–D844 (2020).

32. J. Cheng, G. Novati, J. Pan, C. Bycroft, A. Žemgulytė, T. Applebaum, A. Pritzel, L. H. Wong, M. Zielinski, T. Sargeant, R. G. Schneider, A. W. Senior, J. Jumper, D. Hassabis, P. Kohli, Ž. Avsec, Accurate proteome-wide missense variant effect prediction with AlphaMissense. *Science* **381**, eadg7492 (2023).

33. Schizophrenia Working Group of the Psychiatric Genomics Consortium, Biological insights from 108 schizophrenia-associated genetic loci. *Nature* **511**, 421–427 (2014).

34. N. Carrera, M. Arrojo, J. Sanjuán, R. Ramos-Ríos, E. Paz, J. J. Suárez-Rama, M. Páramo, S. Agra, J. Brenlla, S. Martínez, O. Rivero, D. A. Collier, A. Palotie, S. Cichon, M. M. Nöthen, M. Rietschel, D. Rujescu, H. Stefansson, S. Steinberg, E. Sigurdsson, D. St. Clair, S. Tosato, T. Werge, K. Stefansson, J. C. González, J. Valero, A. Gutiérrez-Zotes, A. Labad, L. Martorell, E. Vilella, Á. Carracedo, J. Costas, Association study of nonsynonymous single nucleotide polymorphisms in schizophrenia. *Biol. Psychiatry* **71**, 169–177 (2012).

35. A. F. Pardiñas, P. Holmans, A. J. Pocklington, V. Escott-Price, S. Ripke, N. Carrera, S. E. Legge, S. Bishop, D. Cameron, M. L. Hamshere, J. Han, L. Hubbard, A. Lynham, K. Mantripragada, E. Rees, J. H. M. Cabe, S. A. Mc Carroll, B. T. Baune, G. Breen, E. M. Byrne, U. Dannlowski, T. C. Eley, C. Hayward, N. G. Martin, A. M. Mc Intosh, R. Plomin, D. J. Porteous, N. R. Wray, A. Caballero, D. H. Geschwind, L. M. Huckins, D. M. Ruderfer, E. Santiago, P. Sklar, E. A. Stahl, H. Won, E. Agerbo, T. D. Als, O. A. Andreassen, M. Bækvad-Hansen, P. B. Mortensen, C. B. Pedersen, A. D. Børglum, J. Bybjerg-Grauholm, S. Djurovic, N. Durmishi, M. G. Pedersen, V. Golimbet, J. Grove, D. M. Hougaard, M. Mattheisen, E. Molden, O. Mors, M. Nordentoft, M. Pejovic-Milovancevic, E. Sigurdsson, T. Silagadze, C. S. Hansen, K. Stefansson, H. Stefansson, S. Steinberg, S. Tosato, T. Werge, GERAD Consortium, CRESTAR Consortium, D. A. Collier, D. Rujescu, G. Kirov, M. J. Owen, M. C. O'Donovan, J. T. R. Walters, Common schizophrenia alleles are enriched in mutation-intolerant genes and in regions under strong background selection. *Nat. Genet.* **50**, 381–389 (2018).

36. D. Li, J.-P. Achkar, T. Haritunians, J. P. Jacobs, K. Y. Hui, M. D'Amato, S. Brand, G. Radford-Smith, J. Halfvarson, J.-H. Niess, S. Kugathasan, C. Büning, L. P. Schumm, L. Klei, A. Ananthakrishnan, G. Aumais, L. Baidoo, M. Dubinsky, C. Fiocchi, J. Glas, R. Milgrom, D. D. Proctor, M. Regueiro, L. A. Simms, J. M. Stempak, S. R. Targan, L. Törkvist, Y. Sharma, B. Devlin, J. Borneman, H. Hakonarson, R. J. Xavier, M. Daly, S. R. Brant, J. D. Rioux, M. S. Silverberg, J. H. Cho, J. Braun, D. P. McGovern, R. H. Duerr, A pleiotropic missense variant in SLC39A8 is associated with Crohn's disease and human gut microbiome composition. *Gastroenterology* **151**, 724–732 (2016).

37. The International Consortium for Blood Pressure Genome-Wide Association Studies, Genetic variants in novel pathways influence blood pressure and cardiovascular disease risk. *Nature* **478**, 103–109 (2011).

38. T. M. Caffrey, R. Wade-Martins, Functional MAPT haplotypes: Bridging the gap between genotype and neuropathology. *Neurobiol. Dis.* **27**, 1–10 (2007).

39. S.-H. Lin, D. W. Brown, M. J. Machiela, LDtrait: An online tool for identifying published phenotype associations in linkage disequilibrium. *Cancer Res.* **80**, 3443–3446 (2020).

40. M. Wainberg, N. J. Forde, S. Mansour, I. Kerrebijn, S. E. Medland, C. Hawco, S. J. Tripathy, Genetic architecture of the structural connectome. *Nat. Commun.* **15**, 1962 (2024).

41. C. A. de Leeuw, J. M. Mooij, T. Heskes, D. Posthuma, MAGMA: Generalized gene-set analysis of GWAS data. *PLOS Comput. Biol.* **11**, e1004219 (2015).

42. T. Rittman, M. Rubinov, P. E. Vértes, A. X. Patel, C. E. Ginestet, B. C. P. Ghosh, R. A. Barker, M. G. Spillantini, E. T. Bullmore, J. B. Rowe, Regional expression of the MAPT gene is associated with loss of hubs in brain networks and cognitive impairment in Parkinson disease and progressive supranuclear palsy. *Neurobiol. Aging* **48**, 153–160 (2016).

43. R. S. Desikan, A. J. Schork, Y. Wang, A. Witoelar, M. Sharma, L. K. McEvoy, D. Holland, J. B. Brewer, C.-H. Chen, W. K. Thompson, D. Harold, J. Williams, M. J. Owen, M. C. O'Donovan, M. A. Pericak-Vance, R. Mayeux, J. L. Haines, L. A. Farrer, G. D. Schellenberg, P. Heutink, A. B. Singleton, A. Brice, N. W. Wood, J. Hardy, M. Martinez, S. H. Choi, A.

DeStefano, M. A. Ikram, J. C. Bis, A. Smith, A. L. Fitzpatrick, L. Launer, C. Van Duijn, S. Seshadri, I. D. Ulstein, D. Aarsland, T. Fladby, S. Djurovic, B. T. Hyman, J. Snaedal, H. Stefansson, K. Stefansson, T. Gasser, O. A. Andreassen, A. M. Dale, ADNI ADGC, GERAD, CHARGE and IPDGC Investigators, Genetic overlap between Alzheimer's disease and Parkinson's disease at the MAPT locus. *Mol. Psychiatry* **20**, 1588–1595 (2015).

44. E. M. Weeks, J. C. Ulirsch, N. Y. Cheng, B. L. Trippe, R. S. Fine, J. Miao, T. A. Patwardhan, M. Kanai, J. Nasser, C. P. Fulco, K. C. Tashman, F. Aguet, T. Li, J. Ordovas-Montanes, C. S. Smillie, M. Biton, A. K. Shalek, A. N. Ananthakrishnan, R. J. Xavier, A. Regev, R. M. Gupta, K. Lage, K. G. Ardlie, J. N. Hirschhorn, E. S. Lander, J. M. Engreitz, H. K. Finucane, Leveraging polygenic enrichments of gene features to predict genes underlying complex traits and diseases. *Nat. Genet.* **55**, 1267–1276 (2023).

45. Z. Sha, D. Schijven, S. E. Fisher, C. Francks, Genetic architecture of the white matter connectome of the human brain. *Sci. Adv.* **9**, eadd2870 (2023).

46. Y. Benjamini, Y. Hochberg, Controlling the false discovery rate: A practical and powerful approach to multiple testing. *J. R. Stat. Soc. B. Methodol.* **57**, 289–300 (1995).

47. Y. Wu, K. S. Burch, A. Ganna, P. Pajukanta, B. Pasaniuc, S. Sankararaman, Fast estimation of genetic correlation for biobank-scale data. *Am. J. Human Genet.* **109**, 24–32 (2022).

48. C. Palmer, I. Pe'er, Statistical correction of the Winner's Curse explains replication variability in quantitative trait genome-wide association studies. *PLOS Genet.* **13**, e1006916 (2017).

49. D. van der Meer, J. Rokicki, T. Kaufmann, A. Córdova-Palomera, T. Moberget, D. Alnæs, F. Bettella, O. Frei, N. T. Doan, I. E. Sønderby, O. B. Smeland, I. Agartz, A. Bertolino, J. Bralten, C. L. Brandt, J. K. Buitelaar, S. Djurovic, M. van Donkelaar, E. S. Dørum, T. Espeseth, S. V. Faraone, G. Fernández, S. E. Fisher, B. Franke, B. Haatveit, C. A. Hartman, P. J. Hoekstra, A. K. Håberg, E. G. Jönsson, K. K. Kolskår, S. Le Hellard, M. J. Lund, A. J. Lundervold, A. Lundervold, I. Melle, J. Monereo Sánchez, L. C. Norbom, J. E. Nordvik, L. Nyberg, J. Oosterlaan, M. Papalino, A. Papassotiropoulos, G. Pergola, D. J. F. de Quervain, G. Richard, A.-M. Sanders, P. Selvaggi, E. Shumskaya, V. M. Steen, S. Tønnesen, K. M.

Ulrichsen, M. P. Zwiers, O. A. Andreassen, L. T. Westlye; Alzheimer's Disease Neuroimaging Initiative; Pediatric Imaging, Neurocognition and Genetics Study, Brain scans from 21,297 individuals reveal the genetic architecture of hippocampal subfield volumes. *Mol. Psychiatry* **25**, 3053–3065 (2020).

50. M. Lanfranchi, S. Yandiev, G. Meyer-Dilhet, S. Ellouze, M. Kerkhofs, R. Dos Reis, A. Garcia, C. Blondet, A. Amar, A. Kneppers, H. Polvèche, D. Plassard, M. Foretz, B. Viollet, K. Sakamoto, R. Mounier, C. F. Bourgeois, O. Raineteau, E. Goillot, J. Courchet, The AMPK-related kinase NUAK1 controls cortical axons branching by locally modulating mitochondrial metabolic functions. *Nat. Commun.* **15**, 2487 (2024).

51. C. Makowski, H. Wang, A. Srinivasan, A. Qi, Y. Qiu, D. van der Meer, O. Frei, J. Zou, P. M. Visscher, J. Yang, C.-H. Chen, Larger cerebral cortex is genetically correlated with greater frontal area and dorsal thickness. *Proc. Natl. Acad. Sci. U.S.A.* **120**, e2214834120 (2023).

52. A. Okbay, Y. Wu, N. Wang, H. Jayashankar, M. Bennett, S. M. Nehzati, J. Sidorenko, H. Kweon, G. Goldman, T. Gjorgjieva, Y. Jiang, B. Hicks, C. Tian, D. A. Hinds, R. Ahlskog, P. K. E. Magnusson, S. Oskarsson, C. Hayward, A. Campbell, D. J. Porteous, J. Freese, P. Herd, C. Watson, J. Jala, D. Conley, P. D. Koellinger, M. Johannesson, D. Laibson, M. N. Meyer, J. J. Lee, A. Kong, L. Yengo, D. Cesarini, P. Turley, P. M. Visscher, J. P. Beauchamp, D. J. Benjamin, A. I. Young, Polygenic prediction of educational attainment within and between families from genome-wide association analyses in 3 million individuals. *Nat. Genet.* **54**, 437–449 (2022).

53. M. P. M. Soutar, D. Melandri, B. O'Callaghan, E. Annuario, A. E. Monaghan, N. J. Welsh, K. D'Sa, S. Guelfi, D. Zhang, A. Pittman, D. Trabzuni, A. H. A. Verboven, K. S. Pan, D. A. Kia, M. Bictash, S. Gandhi, H. Houlden, M. R. Cookson, N. N. Kasri, N. W. Wood, A. B. Singleton, J. Hardy, P. J. Whiting, C. Blauwendraat, A. J. Whitworth, C. Manzoni, M. Ryten, P. A. Lewis, H. Plun-Favreau, Regulation of mitophagy by the NSL complex underlies genetic risk for Parkinson's disease at 16q11.2 and MAPT H1 loci. *Brain* **145**, 4349–4367 (2022).

54. M. Baker, I. Litvan, H. Houlden, J. Adamson, D. Dickson, J. Perez-Tur, J. Hardy, T. Lynch, E. Bigio, M. Hutton, Association of an extended haplotype in the tau gene with progressive supranuclear palsy. *Hum. Mol. Genet.* **8**, 711–715 (1999).

55. A. M. Pittman, A. J. Myers, J. Duckworth, L. Bryden, M. Hanson, P. Abou-Sleiman, N. W. Wood, J. Hardy, A. Lees, R. de Silva, The structure of the tau haplotype in controls and in progressive supranuclear palsy. *Hum. Mol. Genet.* **13**, 1267–1274 (2004).

56. A. M. Pittman, A. J. Myers, P. Abou-Sleiman, H. C. Fung, M. Kaleem, L. Marlowe, J. Duckworth, D. Leung, D. Williams, L. Kilford, N. Thomas, C. M. Morris, D. Dickson, N. W. Wood, J. Hardy, A. J. Lees, R. de Silva, Linkage disequilibrium fine mapping and haplotype association analysis of the tau gene in progressive supranuclear palsy and corticobasal degeneration. *J. Med. Genet.* **42**, 837–846 (2005).

57. P. Pastor, M. Ezquerra, J. C. Perez, S. Chakraverty, J. Norton, B. A. Racette, D. McKeel, J. S. Perlmutter, E. Tolosa, A. M. Goate, Novel haplotypes in 17q21 are associated with progressive supranuclear palsy. *Ann. Neurol.* **56**, 249–258 (2004).

58. M. G. Spillantini, J. R. Murrell, M. Goedert, M. R. Farlow, A. Klug, B. Ghetti, Mutation in the tau gene in familial multiple system tauopathy with presenile dementia. *Proc. Natl. Acad. Sci. U.S.A.* **95**, 7737–7741 (1998).

59. J. Simón-Sánchez, C. Schulte, J. M. Bras, M. Sharma, J. R. Gibbs, D. Berg, C. Paisan-Ruiz, P. Lichtner, S. W. Scholz, D. G. Hernandez, R. Krüger, M. Federoff, C. Klein, A. Goate, J. Perlmutter, M. Bonin, M. A. Nalls, T. Illig, C. Gieger, H. Houlden, M. Steffens, M. S. Okun, B. A. Racette, M. R. Cookson, K. D. Foote, H. H. Fernandez, B. J. Traynor, S. Schreiber, S. Arepalli, R. Zonozi, K. Gwinn, M. van der Brug, G. Lopez, S. J. Chanock, A. Schatzkin, Y. Park, A. Hollenbeck, J. Gao, X. Huang, N. W. Wood, D. Lorenz, G. Deuschl, H. Chen, O. Riess, J. A. Hardy, A. B. Singleton, T. Gasser, Genome-wide association study reveals genetic risk underlying Parkinson's disease. *Nat. Genet.* **41**, 1308–1312 (2009).

60. P. Sánchez-Juan, S. Moreno, I. de Rojas, I. Hernández, S. Valero, M. Alegret, L. Montrreal, P. García González, C. Lage, S. López-García, E. Rodríiguez-Rodríguez, A. Orellana, L.

Tárraga, M. Boada, A. Ruiz, The MAPT H1 haplotype is a risk factor for Alzheimer's disease in APOE ε4 non-carriers. *Front. Aging Neurosci.* **11**, 327 (2019).

61. N. Kouri, O. A. Ross, B. Dombroski, C. S. Younkin, D. J. Serie, A. Soto-Ortolaza, M. Baker, N. C. A. Finch, H. Yoon, J. Kim, S. Fujioka, C. A. McLean, B. Ghetti, S. Spina, L. B. Cantwell, M. R. Farlow, J. Grafman, E. D. Huey, M. Ryung Han, S. Beecher, E. T. Geller, H. A. Kretzschmar, S. Roeber, M. Gearing, J. L. Juncos, J. P. G. Vonsattel, V. M. Van Deerlin, M. Grossman, H. I. Hurtig, R. G. Gross, S. E. Arnold, J. Q. Trojanowski, V. M. Lee, G. K. Wenning, C. L. White, G. U. Höglinger, U. Müller, B. Devlin, L. I. Golbe, J. Crook, J. E. Parisi, B. F. Boeve, K. A. Josephs, Z. K. Wszolek, R. J. Uitti, N. R. Graff-Radford, I. Litvan, S. G. Younkin, L.-S. Wang, N. Ertekin-Taner, R. Rademakers, H. Hakonarsen, G. D. Schellenberg, D. W. Dickson, Genome-wide association study of corticobasal degeneration identifies risk variants shared with progressive supranuclear palsy. *Nat. Commun.* **6**, 7247 (2015).

62. C. Bellenguez, F. Küçükali, I. E. Jansen, L. Kleineidam, S. Moreno-Grau, N. Amin, A. C. Naj, R. Campos-Martin, B. Grenier-Boley, V. Andrade, P. A. Holmans, A. Boland, V. Damotte, S. J. van der Lee, M. R. Costa, T. Kuulasmaa, Q. Yang, I. de Rojas, J. C. Bis, A. Yaqub, I. Prokic, J. Chapuis, S. Ahmad, V. Giedraitis, D. Aarsland, P. Garcia-Gonzalez, C. Abdelnour, E. Alarcón-Martín, D. Alcolea, M. Alegret, I. Alvarez, V. Álvarez, N. J. Armstrong, A. Tsolaki, C. Antúnez, I. Appollonio, M. Arcaro, S. Archetti, A. A. Pastor, B. Arosio, L. Athanasiu, H. Bailly, N. Banaj, M. Baquero, S. Barral, A. Beiser, A. B. Pastor, J. E. Below, P. Benchek, L. Benussi, C. Berr, C. Besse, V. Bessi, G. Binetti, A. Bizarro, R. Blesa, M. Boada, E. Boerwinkle, B. Borroni, S. Boschi, P. Bossù, G. Bråthen, J. Bressler, C. Bresner, H. Brodaty, K. J. Brookes, L. I. Brusco, D. Buiza-Rueda, K. Bûrger, V. Burholt, W. S. Bush, M. Calero, L. B. Cantwell, G. Chene, J. Chung, M. L. Cuccaro, Á. Carracedo, R. Cecchetti, L. Cervera-Carles, C. Charbonnier, H.-H. Chen, C. Chillotti, S. Ciccone, J. A. H. R. Claassen, C. Clark, E. Conti, A. Corma-Gómez, E. Costantini, C. Custodero, D. Daian, M. C. Dalmasso, A. Daniele, E. Dardiotis, J.-F. Dartigues, P. P. de Deyn, K. de Paiva Lopes, L. D. de Witte, S. Debette, J. Deckert, T. del Ser, N. Denning, A. DeStefano, M. Dichgans, J. Diehl-Schmid, M. Diez-Fairen, P. D. Rossi, S. Djurovic, E. Duron, E. Düzel, C. Dufouil, G. Eiriksdottir, S. Engelborghs, V. Escott-Price, A. Espinosa, M. Ewers, K. M. Faber, T. Fabrizio, S. F. Nielsen, D. W. Fardo, L. Farotti, C. Fenoglio, M. Fernández-Fuertes, R.

Ferrari, C. B. Ferreira, E. Ferri, B. Fin, P. Fischer, T. Fladby, K. Fließbach, B. Fongang, M. Fornage, J. Fortea, T. M. Foroud, S. Fostinelli, N. C. Fox, E. Franco-Macías, M. J. Bullido, A. Frank-García, L. Froelich, B. Fulton-Howard, D. Galimberti, J. M. García-Alberca, P. García-González, S. Garcia-Madrona, G. Garcia-Ribas, R. Ghidoni, I. Giegling, G. Giorgio, A. M. Goate, O. Goldhardt, D. Gomez-Fonseca, A. González-Pérez, C. Graff, G. Grande, E. Green, T. Grimmer, E. Grünblatt, M. Grunin, V. Gudnason, T. Guetta-Baranes, A. Haapasalo, G. Hadjigeorgiou, J. L. Haines, K. L. Hamilton-Nelson, H. Hampel, O. Hanon, J. Hardy, A. M. Hartmann, L. Hausner, J. Harwood, S. Heilmann-Heimbach, S. Helisalmi, M. T. Heneka, I. Hernández, M. J. Herrmann, P. Hoffmann, C. Holmes, H. Holstege, R. H. Vilas, M. Hulsman, J. Humphrey, G. J. Biessels, X. Jian, C. Johansson, G. R. Jun, Y. Kastumata, J. Kauwe, P. G. Kehoe, L. Kilander, A. K. Ståhlbom, M. Kivipelto, A. Koivisto, J. Kornhuber, M. H. Kosmidis, W. A. Kukull, P. P. Kuksa, B. W. Kunkle, A. B. Kuzma, C. Lage, E. J. Laukka, L. Launer, A. Lauria, C.-Y. Lee, J. Lehtisalo, O. Lerch, A. Lleó, W. Longstreth, O. Lopez, A. L. de Munain, S. Love, M. Löwemark, L. Luckcuck, K. L. Lunetta, Y. Ma, J. Macías, C. A. MacLeod, W. Maier, F. Mangialasche, M. Spallazzi, M. Marquié, R. Marshall, E. R. Martin, A. M. Montes, C. M. Rodríguez, C. Masullo, R. Mayeux, S. Mead, P. Mecocci, M. Medina, A. Meggy, S. Mehrabian, S. Mendoza, M. Menéndez-González, P. Mir, S. Moebus, M. Mol, L. Molina-Porcel, L. Montrreal, L. Morelli, F. Moreno, K. Morgan, T. Mosley, M. M. Nöthen, C. Muchnik, S. Mukherjee, B. Nacmias, T. Ngandu, G. Nicolas, B. G. Nordestgaard, R. Olaso, A. Orellana, M. Orsini, G. Ortega, A. Padovani, C. Paolo, G. Papenberg, L. Parnetti, F. Pasquier, P. Pastor, G. Peloso, A. Pérez-Cordón, J. Pérez-Tur, P. Pericard, O. Peters, Y. A. L. Pijnenburg, J. A. Pineda, G. Piñol-Ripoll, C. Pisanu, T. Polak, J. Popp, D. Posthuma, J. Priller, R. Puerta, O. Quenez, I. Quintela, J. Q. Thomassen, A. Rábano, I. Rainero, F. Rajabli, I. Ramakers, L. M. Real, M. J. T. Reinders, C. Reitz, D. Reyes-Dumeyer, P. Ridge, S. Riedel-Heller, P. Riederer, N. Roberto, E. Rodriguez-Rodriguez, A. Rongve, I. R. Allende, M. Rosende-Roca, J. L. Royo, E. Rubino, D. Rujescu, M. E. Sáez, P. Sakka, I. Saltvedt, Á. Sanabria, M. B. Sánchez-Arjona, F. Sanchez-Garcia, P. S. Juan, R. Sánchez-Valle, S. B. Sando, C. Sarnowski, C. L. Satizabal, M. Scamosci, N. Scarmeas, E. Scarpini, P. Scheltens, N. Scherbaum, M. Scherer, M. Schmid, A. Schneider, J. M. Schott, G. Selbæk, D. Seripa, M. Serrano, J. Sha, A. A. Shadrin, O. Skrobot, S. Slifer, G. J. L. Snijders, H. Soininen, V. Solfrizzi, A. Solomon, Y. Song, S. Sorbi, O. Sotolongo-Grau, G. Spalletta, A. Spottke, A. Squassina, E. Stordal, J. P. Tartan, L. Tárraga, N. Tesí, A. Thalamuthu, T.

Thomas, G. Tosto, L. Traykov, L. Tremolizzo, A. Tybjærg-Hansen, A. Uitterlinden, A. Ullgren, I. Ulstein, S. Valero, O. Valladares, C. V. Broeckhoven, J. Vance, B. N. Vardarajan, A. van der Lugt, J. V. Dongen, J. van Rooij, J. van Swieten, R. Vandenberghe, F. Verhey, J.-S. Vidal, J. Vogelgsang, M. Vyhnalek, M. Wagner, D. Wallon, L.-S. Wang, R. Wang, L. Weinhold, J. Wiltfang, G. Windle, B. Woods, M. Yannakoulia, H. Zare, Y. Zhao, X. Zhang, C. Zhu, M. Zulaica, EADB, GR@ACE, DEGESCO, EADI, GERAD, Demgene, FinnGen, ADGC, CHARGE, L. A. Farrer, B. M. Psaty, M. Ghanbari, T. Raj, P. Sachdev, K. Mather, F. Jessen, M. A. Ikram, A. de Mendonça, J. Hort, M. Tsolaki, M. A. Pericak-Vance, P. Amouyel, J. Williams, R. Frikke-Schmidt, J. Clarimon, J.-F. Deleuze, G. Rossi, S. Seshadri, O. A. Andreassen, M. Ingelsson, M. Hiltunen, K. Sleegers, G. D. Schellenberg, C. M. van Duijn, R. Sims, W. M. van der Flier, A. Ruiz, A. Ramirez, J.-C. Lambert, New insights into the genetic etiology of Alzheimer's disease and related dementias. *Nat. Genet.* **54**, 412–436 (2022).

63. W. Huang, J. Zeng, L. Jia, D. Zhu, J. O'Brien, C. Ritchie, N. Shu, L. Su, Genetic risks of Alzheimer's by APOE and MAPT on cortical morphology in young healthy adults. *Brain Commun.* **5**, fcad234 (2023).

64. J. H. Lee, J. Ryan, C. Andreescu, H. Aizenstein, H. K. Lim, Brainstem morphological changes in Alzheimer's disease. *Neuroreport* **26**, 411–415 (2015).

65. P. Theofilas, A. J. Ehrenberg, S. Dunlop, A. T. Alho, A. Nguy, R. E. P. Leite, R. D. Rodriguez, M. B. Mejia, C. K. Suemoto, R. E. de Lucena Ferretti-Rebustini, L. Polichiso, C. F. Nascimento, W. W. Seeley, R. Nitrini, C. A. Pasqualucci, W. J. Filho, U. Rueb, J. Neuhaus, H. Heinsen, L. T. Grinberg, Locus coeruleus volume and cell population changes during Alzheimer's disease progression: A stereological study in human postmortem brains with potential implication for early-stage biomarker discovery. *Alzheimers Dement.* **13**, 236–246 (2017).

66. A. J. Ehrenberg, C. K. Suemoto, E. de Paula França Resende, C. Petersen, R. E. P. Leite, R. D. Rodriguez, R. E. de Lucena Ferretti-Rebustini, M. You, J. Oh, R. Nitrini, C. A. Pasqualucci, W. Jacob-Filho, J. H. Kramer, J. R. Gatchel, L. T. Grinberg, Neuropathologic correlates of psychiatric symptoms in Alzheimer's disease. *J. Alzheimers Dis.* **66**, 115–126 (2018).

67. D. A. Koolen, R. Pfundt, K. Linda, G. Beunders, H. E. Veenstra-Knol, J. H. Conta, A. M. Fortuna, G. Gillessen-Kaesbach, S. Dugan, S. Halbach, O. A. Abdul-Rahman, H. M. Winesett, W. K. Chung, M. Dalton, P. S. Dimova, T. Mattina, K. Prescott, H. Z. Zhang, H. M. Saal, J. Y. Hehir-Kwa, M. H. Willemsen, C. W. Ockeloen, M. C. Jongmans, N. Van der Aa, P. Failla, C. Barone, E. Avola, A. S. Brooks, S. G. Kant, E. H. Gerkes, H. V. Firth, K. Õunap, L. M. Bird, D. Masser-Frye, J. R. Friedman, M. A. Sokunbi, A. Dixit, M. Splitt, M. K. Kukolich, J. McGaughran, B. P. Coe, J. Flórez, N. Nadif Kasri, H. G. Brunner, E. M. Thompson, J. Gecz, C. Romano, E. E. Eichler, B. B. de Vries, The Koolen-de Vries syndrome: A phenotypic comparison of patients with a 17q21.31 microdeletion versus a KANSL1 sequence variant. *Eur. J. Hum. Genet.* **24**, 652–659 (2016).

68. J. Xu, N. Liu, E. Polemiti, L. Garcia-Mondragon, J. Tang, X. Liu, T. Lett, L. Yu, M. M. Nöthen, J. Feng, C. Yu, A. Marquand, G. Schumann, the environMENTAL Consortium, Effects of urban living environments on mental health in adults. *Nat. Med.* **29**, 1456–1467 (2023).

69. A. C. H. Chen, N. Manz, Y. Tang, M. Rangaswamy, L. Almasy, S. Kuperman, J. Nurnberger Jr., S. J. O'Connor, H. J. Edenberg, M. A. Schuckit, J. Tischfield, T. Foroud, L. J. Bierut, J. Rohrbaugh, J. P. Rice, A. Goate, V. Hesselbrock, B. Porjesz, Single-nucleotide polymorphisms in corticotropin releasing hormone receptor 1 gene (CRHR1) are associated with quantitative trait of event-related potential and alcohol dependence. *Alcohol. Clin. Exp. Res.* **34**, 988–996 (2010).

70. D. Blomeyer, J. Treutlein, G. Esser, M. H. Schmidt, G. Schumann, M. Laucht, Interaction between CRHR1 gene and stressful life events predicts adolescent heavy alcohol use. *Biol. Psychiatry* **63**, 146–151 (2008).

71. R. Daviet, G. Aydogan, K. Jagannathan, N. Spilka, P. D. Koellinger, H. R. Kranzler, G. Nave, R. R. Wetherill, Associations between alcohol consumption and gray and white matter volumes in the UK Biobank. *Nat. Commun.* **13**, 1175 (2022).

72. A. Thompson, J. Cook, H. Choquet, E. Jorgenson, J. Yin, T. Kinnunen, J. Barclay, A. P. Morris, M. Pirmohamed, Functional validity, role, and implications of heavy alcohol consumption genetic loci. *Sci. Adv.* **6**, eaay5034 (2020).

73. T. Chambers, V. Escott-Price, S. Legge, E. Baker, K. D. Singh, J. T. R. Walters, X. Caseras, R. J. L. Anney, Genetic common variants associated with cerebellar volume and their overlap with mental disorders: a study on 33,265 individuals from the UK-Biobank. *Mol. Psychiatry* **27**, 2282–2290 (2022).

74. N. C. Andreasen, R. Pierson, The role of the cerebellum in schizophrenia. *Biol. Psychiatry* **64**, 81–88 (2008).

75. T. G. M. van Erp, D. P. Hibar, J. M. Rasmussen, D. C. Glahn, G. D. Pearlson, O. A. Andreassen, I. Agartz, L. T. Westlye, U. K. Haukvik, A. M. Dale, I. Melle, C. B. Hartberg, O. Gruber, B. Kraemer, D. Zilles, G. Donohoe, S. Kelly, C. McDonald, D. W. Morris, D. M. Cannon, A. Corvin, M. W. J. Machielsen, L. Koenders, L. de Haan, D. J. Veltman, T. D. Satterthwaite, D. H. Wolf, R. C. Gur, R. E. Gur, S. G. Potkin, D. H. Mathalon, B. A. Mueller, A. Preda, F. Macciardi, S. Ehrlich, E. Walton, J. Hass, V. D. Calhoun, H. J. Bockholt, S. R. Sponheim, J. M. Shoemaker, N. E. M. van Haren, H. E. H. Pol, R. A. Ophoff, R. S. Kahn, R. Roiz-Santiañez, B. Crespo-Facorro, L. Wang, K. I. Alpert, E. G. Jönsson, R. Dimitrova, C. Bois, H. C. Whalley, A. M. McIntosh, S. M. Lawrie, R. Hashimoto, P. M. Thompson, J. A. Turner, Subcortical brain volume abnormalities in 2028 individuals with schizophrenia and 2540 healthy controls via the ENIGMA consortium. *Mol. Psychiatry* **21**, 547–553 (2016).

76. N. Okada, M. Fukunaga, K. Miura, K. Nemoto, J. Matsumoto, N. Hashimoto, M. Kiyota, K. Morita, D. Koshiyama, K. Ohi, T. Takahashi, M. Koeda, H. Yamamori, M. Fujimoto, Y. Yasuda, N. Hasegawa, H. Narita, S. Yokoyama, R. Mishima, T. Kawashima, Y. Kobayashi, D. Sasabayashi, K. Harada, M. Yamamoto, Y. Hirano, T. Itahashi, M. Nakataki, R. Hashimoto, K. K. Tha, S. Koike, T. Matsubara, G. Okada, T. G. M. van Erp, N. Jahanshad, R. Yoshimura, O. Abe, T. Onitsuka, Y. Watanabe, K. Matsuo, H. Yamasue, Y. Okamoto, M. Suzuki, J. A. Turner, P. M. Thompson, N. Ozaki, K. Kasai, R. Hashimoto, Subcortical volumetric alterations in four major psychiatric disorders: a mega-analysis study of 5604 subjects and a volumetric data-driven approach for classification. *Mol. Psychiatry* **28**, 5206–5216 (2023).

77. Y. Jiang, C. Luo, J. Wang, L. Palaniyappan, X. Chang, S. Xiang, J. Zhang, M. Duan, H. Huang, C. Gaser, K. Nemoto, K. Miura, R. Hashimoto, L. T. Westlye, G. Richard, S.

Fernandez-Cabello, N. Parker, O. A. Andreassen, T. Kircher, I. Nenadić, F. Stein, F. Thomas-Odenthal, L. Teutenberg, P. Usemann, U. Dannlowski, T. Hahn, D. Grotegerd, S. Meinert, R. Lencer, Y. Tang, T. Zhang, C. Li, W. Yue, Y. Zhang, X. Yu, E. Zhou, C.-P. Lin, S.-J. Tsai, A. L. Rodrigue, D. Glahn, G. Pearlson, J. Blangero, A. Karuk, E. Pomarol-Clotet, R. Salvador, P. Fuentes-Claramonte, M. Á. Garcia-León, G. Spalletta, F. Piras, D. Vecchio, N. Banaj, J. Cheng, Z. Liu, J. Yang, A. S. Gonul, O. Uslu, B. B. Burhanoglu, A. Uyar Demir, K. Rootes-Murdy, V. D. Calhoun, K. Sim, M. Green, Y. Quidé, Y. C. Chung, W.-S. Kim, S. R. Sponheim, C. Demro, I. S. Ramsay, F. Iasevoli, A. de Bartolomeis, A. Barone, M. Ciccarelli, A. Brunetti, S. Cocozza, G. Pontillo, M. Tranfa, M. T. M. Park, M. Kirschner, F. Georgiadis, S. Kaiser, T. E. Van Rheenen, S. L. Rossell, M. Hughes, W. Woods, S. P. Carruthers, P. Sumner, E. Ringin, F. Spaniel, A. Skoch, D. Tomecek, P. Homan, S. Homan, W. Omlor, G. Cecere, D. D. Nguyen, A. Preda, S. I. Thomopoulos, N. Jahanshad, L.-B. Cui, D. Yao, P. M. Thompson, J. A. Turner, T. G. M. van Erp, W. Cheng, J. Feng, ZIB Consortium, Neurostructural subgroup in 4291 individuals with schizophrenia identified using the subtype and stage inference algorithm. *Nat. Commun.* **15**, 5996 (2024).

78. J. R. Glausier, D. A. Lewis, Dendritic spine pathology in schizophrenia. *Neuroscience* **251**, 90–107 (2013).

79. S. Li, C. Ma, Y. Li, R. Chen, Y. Liu, L. P. Wan, Q. Xiong, C. Wang, Y. Huo, X. Dang, Y. Yang, L. Lv, X. Chen, N. Sheng, W. Li, X.-J. Luo, The schizophrenia-associated missense variant rs13107325 regulates dendritic spine density. *Transl. Psychiatry* **12**, 1–12 (2022).

80. E.-M. Stauffer, R. A. I. Bethlehem, L. Dorfschmidt, H. Won, V. Warrier, E. T. Bullmore, The genetic relationships between brain structure and schizophrenia. *Nat. Commun.* **14**, 7820 (2023).

81. Cross-Disorder Group of the Psychiatric Genomics Consortium, Genetic relationship between five psychiatric disorders estimated from genome-wide SNPs. *Nat. Genet.* **45**, 984–994 (2013).

82. M. Madre, E. J. Canales-Rodríguez, P. Fuentes-Claramonte, S. Alonso-Lana, P. Salgado-Pineda, A. Guerrero-Pedraza, N. Moro, C. Bosque, J. J. Gomar, J. Ortíz-Gil, J. M. Goikolea, C. M. Bonnin, E. Vieta, S. Sarró, T. Maristany, P. J. McKenna, R. Salvador, E. Pomarol-Clotet, Structural abnormality in schizophrenia versus bipolar disorder: A whole brain

cortical thickness, surface area, volume and gyrification analyses. *Neuroimage Clin.* **25**, 102131 (2020).

83. M. Canavan, M. J. O'Donnell, Hypertension and cognitive impairment: A review of mechanisms and key concepts. *Front. Neurol.* **13**, 821135 (2022).

84. L. M. Jenkins, C. R. Garner, S. Kurian, J. P. Higgins, T. B. Parrish, S. Sedaghat, A. J. Nemeth, D. M. Lloyd-Jones, L. J. Launer, J. M. Hausdorff, L. Wang, F. A. Sorond, Cumulative blood pressure exposure, basal ganglia, and thalamic morphology in midlife. *Hypertension* **75**, 1289–1295 (2020).

85. Y. Halchenko, K. Meyer, B. Poldrack, D. Solanky, A. Wagner, J. Gors, D. MacFarlane, D. Pustina, V. Sochat, S. Ghosh, C. Mönch, C. Markiewicz, L. Waite, I. Shlyakhter, A. De La Vega, S. Hayashi, C. Häusler, J.-B. Poline, T. Kadelka, K. Skytén, D. Jarecka, D. Kennedy, T. Strauss, M. Cieslak, P. Vavra, H.-I. Ioanas, R. Schneider, M. Pflüger, J. Haxby, S. Eickhoff, M. Hanke, DataLad: Distributed system for joint management of code, data, and their relationship. *J. Open Source Softw.* **6**, 3262 (2021).

86. C. Bycroft, C. Freeman, D. Petkova, G. Band, L. T. Elliott, K. Sharp, A. Motyer, D. Vukcevic, O. Delaneau, J. O'Connell, A. Cortes, S. Welsh, A. Young, M. Effingham, G. McVean, S. Leslie, N. Allen, P. Donnelly, J. Marchini, The UK Biobank resource with deep phenotyping and genomic data. *Nature* **562**, 203–209 (2018).

87. M. Mills, N. Barban, F. C. Tropf, *An Introduction to Statistical Genetic Data Analysis* (The MIT Press, 2020).

88. J. Graffelman, V. Moreno, The mid p-value in exact tests for Hardy-Weinberg equilibrium. *Stat. Appl. Genet. Mol. Biol.* **12**, 433–448 (2013).

89. A. M. Dale, M. I. Sereno, Improved Localizadon of Cortical Activity by Combining EEG and MEG with MRI Cortical Surface Reconstruction: A Linear Approach. *J. Cogn. Neurosci.* **5**, 162–176 (1993).

90. A. M. Dale, B. Fischl, M. I. Sereno, Cortical Surface-Based Analysis: I Segmentation and Surface Reconstruction. *NeuroImage* **9**, 179–194 (1999).

91. B. Fischl, M. I. Sereno, A. M. Dale, Cortical Surface-Based Analysis: II: Inflation, Flattening, and a Surface-Based Coordinate System. *Neuroimage* **9**, 195–207 (1999).

92. B. Fischl, M. I. Sereno, R. B. H. Tootell, A. M. Dale, High-resolution intersubject averaging and a coordinate system for the cortical surface. *Hum. Brain Mapp.* **8**, 272–284 (1999).

93. B. Fischl, D. H. Salat, E. Busa, M. Albert, M. Dieterich, C. Haselgrove, A. van der Kouwe, R. Killiany, D. Kennedy, S. Klaveness, A. Montillo, N. Makris, B. Rosen, A. M. Dale, Whole Brain Segmentation: Automated Labeling of Neuroanatomical Structures in the Human Brain. *Neuron* **33**, 341–355 (2002).

94. The 1000 Genomes Project Consortium, A global reference for human genetic variation. *Nature* **526**, 68–74 (2015).

95. S. A. Lambert, L. Gil, S. Jupp, S. C. Ritchie, Y. Xu, A. Buniello, A. McMahon, G. Abraham, M. Chapman, H. Parkinson, J. Danesh, J. A. L. MacArthur, M. Inouye, The Polygenic Score Catalog as an open database for reproducibility and systematic evaluation. *Nat. Genet.* **53**, 420–425 (2021).

96. D. Lai, E. C. Johnson, S. Colbert, G. Pandey, G. Chan, L. Bauer, M. W. Francis, V. Hesselbrock, C. Kamarajan, J. Kramer, W. Kuang, S. Kuo, S. Kuperman, Y. Liu, V. McCutcheon, Z. Pang, M. H. Plawecki, M. Schuckit, J. Tischfield, L. Wetherill, Y. Zang, H. J. Edenberg, B. Porjesz, A. Agrawal, T. Foroud, Evaluating risk for alcohol use disorder: Polygenic risk scores and family history. *Alcohol. Clin. Exp. Res.* **46**, 374–383 (2022).

97. G. Molenberghs, G. Verbeke, Likelihood ratio, score, and Wald tests in a constrained parameter space. *Am. Stat.* **61**, 22–27 (2007).

98. M. S. Mufford, D. van der Meer, T. Kaufmann, O. Frei, R. Ramesar, P. M. Thompson, N. Jahanshad, R. A. Morey, O. A. Andreassen, D. J. Stein, S. Dalvie, The genetic architecture of amygdala nuclei. *Biol. Psychiatry* **95**, 72–84 (2024).
